# Supplementary material for: Investigating the safety and activity of the use of BTT1023 (Timolumab), in the treatment of patients with primary sclerosing cholangitis (BUTEO): A single-arm, two-stage, open-label, multi-centre, phase II clinical trial protocol
Source: BMJ Open. 2017 Jul 3;7(6):e015081. doi: 10.1136/bmjopen-2016-015081 (PMC5734279; doi:10.1136/bmjopen-2016-015081)
Supplement: Author's manuscript [file bmjopen-2016-015081.draft_revisions.pdf]

BMJ Open

Investigating the safety and activity of the use of BTT1023 (Timolumab), a human monoclonal antibody targeting vascular adhesion protein-1, in the treatment of patients with primary sclerosing cholangitis (BUTEO): A single arm, two-stage, open-label, multi-centre, phase II clinical trial.

Journal:	*BMJ Open*
Manuscript ID	bmjopen-2016-015081
Article Type:	Protocol
Date Submitted by the Author:	08-Nov-2016
Complete List of Authors:	Arndtz, Katherine; University of Birmingham, Institute of Immunology & Immunotherapy<br>Corrigan, Margaret; Centre for Liver Research, University of Birmingham and NIHR Birmingham Liver Biomedical Research Unit<br>Rowe, Anna; NIHR Birmingham Liver Biomedical Research Unit Clinical Trials Group (D3B team), CRUK Clinical Trials Unit, University of Birmingham, Birmingham, UK<br>Hirschfield, Gideon; University of Birmingham, School of Immunity and Infection<br>Kirkham, Amanda; Statistics, CRUK Clinical Trials Unit, University of Birmingham, Birmingham, UK<br>Barton, Darren; University of Birmingham, NIHR Liver BRU Clinical trials group (EDD), CRUK clinical trials unit<br>Fox, Richard; Cancer Research Clinical Trials Unit, Biostatistics<br>Llewellyn, Laura; Early Drug Development Team, CRUK Clinical Trials Unit, University of Birmingham, Birmingham, UK<br>Athwal, Amrita; NIHR Birmingham Liver Biomedical Research Unit Clinical Trials Group (D3B team), CRUK Clinical Trials Unit, University of Birmingham, Birmingham, UK<br>Wilkhu, Manpreet; NIHR Birmingham Liver Biomedical Research Unit Clinical Trials Group (D3B team), CRUK Clinical Trials Unit, University of Birmingham, Birmingham, UK<br>Chen, Yung-Yi; NIHR Liver Biomedical Research Unit, Centre for Liver Research, University of Birmingham, Birmingham, UK<br>Weston, Chris; NIHR Liver Biomedical Research Unit, Centre for Liver Research, University of Birmingham, Birmingham, UK<br>Desai, Amisha<br>Adams, David; NIHR Liver Biomedical Research Unit, Centre for Liver Research, University of Birmingham, Birmingham, UK
**Primary Subject Heading**:	Gastroenterology and hepatology
Secondary Subject Heading:	Immunology (including allergy)
Keywords:	Immunology < BASIC SCIENCES, Hepatobiliary disease < GASTROENTEROLOGY, IMMUNOLOGY, Hepatology < INTERNAL MEDICINE, Clinical trials < THERAPEUTICS

SCHOLARONE™
Manuscripts

For peer review only

Investigating the safety and activity of the use of BTT1023 (Timolumab), a human monoclonal antibody targeting vascular adhesion protein-1, in the treatment of patients with primary sclerosing cholangitis (BUTEO): A single arm, two-stage, open-label, multi-centre, phase II clinical trial.

Katherine Arndtz[1], Margaret Corrigan[1], Anna Rowe[2], Amanda Kirkham[3], Darren Barton[2], Richard Fox[3], Laura Llewellyn[4], Amrita Athwal[2], Manpreet Wilkhu[2], Yung Yi Chen[1], Chris Weston[1], Amisha Desai[5], David H Adams[1], Gideon M Hirschfield[1], on behalf of the BUTEO trial team.

[1]NIHR Liver Biomedical Research Unit, Centre for Liver Research, University of Birmingham, Birmingham, and Birmingham Health Partners, Institute of Translational Medicine, University Hospitals Birmingham, Birmingham, UK

[2]NIHR Birmingham Liver Biomedical Research Unit Clinical Trials Group (D[3]B team), CRUK Clinical Trials Unit, University of Birmingham, Birmingham, UK

[3]Statistics, CRUK Clinical Trials Unit, University of Birmingham, Birmingham, UK

[4]Early Drug Development Team, CRUK Clinical Trials Unit, University of Birmingham, Birmingham, UK

[5]Pharmacy, University Hospitals Birmingham NHS Foundation Trust, Queen Elizabeth Hospital Birmingham

Corresponding author: Professor Gideon Hirschfield, Centre for Liver Research, Institute of Immunology & Immunotherapy, University of Birmingham, Edgbaston, Birmingham, UK, B15 2TT

Email: g.hirschfield@bham.ac.uk

For peer review only

Keywords (5 max): Autoimmune liver disease; Early phase clinical trials; Anti-fibrotic; Serum amine oxidase

Word count excluding abstract/references: 4952

For peer review only

Abstract

Introduction:

Primary sclerosing cholangitis (PSC) is a progressive inflammatory liver disease characterised by relentless liver fibrosis and a high unmet need for new therapies. Preventing fibrosis represents an important area of interest in the development of vital new drugs. Vascular adhesion protein-1 (VAP-1) drives inflammation in liver disease, and provision of an antibody against VAP-1 blunts fibrosis in murine models of liver injury.

Methods & Analysis:

BUTEO is a single arm, two-stage, open-label, multi-centre, phase II clinical trial. Up to 59 patients will receive treatment with anti-VAP monoclonal antibody, BTT1023, over a 78 day treatment period. Adults with PSC and a serum alkaline phosphatase (ALP) of at least 1.5 times the upper limit of normal will be included. Our primary outcome measure is a reduction in ALP by >25% from baseline to Day 99. Secondary outcome measures include safety and tolerability, changes pre/post therapy in circulating serum VAP-1 as well as imaging findings. The first patient participant was recruited on 08/09/2015.

Ethics & Dissemination:

This protocol has been approved by the Research Ethics Committee (REC, reference 14/EM/1272). The first REC approval date was 06/01/2015 with three subsequent approved amendments. This article refers to protocol version 3.0, dated 16/03/2016. Results will be disseminated via peer reviewed publication and presentation at international conferences.

Trial registration:

The trial is registered with the European Medicines agency (EudraCT: 2014-002393-37), the NIHR (PortfolioID:18051) and ISRCTN: 11233255. The clinicaltrials.gov identifier is NCT02239211.

For peer review only

Strengths:-

- Unique, tailor-made clinical trial design incorporating a dose confirmatory and safety stage (based on the traditional 3+3 design), then followed by a Phase II Simon's two-stage design.
- Brings the translation of laboratory research into a proof of activity clinical trial.
- An early phase experimental medicine study of a novel first-in-class drug, in a chronic disease cohort with a large unmet need for new therapies.
- Aims to address not just the need for new therapies but also the need for reliable clinical trial endpoints as well as biomarkers for staging and predicting clinical outcomes.

Limitations:-

- Small cohort due to PSC being a rare orphan disease as well as unpredictability of the disease making stability for clinical trial inclusion difficult.
- Short duration of the treatment period in which goal is to demonstrate collective markers of efficacy to justify longer and placebo-controlled trials.
- Limited evidence base for the primary endpoint of a reduction in ALP in the context of anti-fibrotic agents, however accepting there is no alternative surrogate currently available.
- Translational study from mice into human subject and the unknown differences this may entail.

For peer review only

INTRODUCTION

End-stage liver disease, regardless of aetiology, is characterised by progressive hepatic fibrosis culminating in liver cirrhosis and accompanying increased risks of liver cancer, liver failure, portal hypertension and death. Preventing progressive liver fibrosis represents an important area of interest in the development of new drugs suitable for all patients with liver disease. Primary sclerosing cholangitis (PSC) is a prime example of a progressive inflammatory liver disease which is characterised by persistent liver fibrosis and a high unmet need for new therapies. PSC has a population incidence of 1.3/100,000 annually, with a prevalence of 16.2 per 100,000[1,2,3]. It affects both men and women, with a median age of 41[4] and in 80% of cases is associated with Inflammatory Bowel Disease (IBD)[5]. More than 50% of patients require liver transplantation within 10-15 years of symptomatic presentation[6,7], reflecting the failure of medical therapies to have any impact on the clinical outcome: in the UK for example, PSC is now the leading autoimmune liver disease indication for transplant, despite being the rarest of the autoimmune liver diseases. One barrier to the development of efficacious new medical therapies is the lack of clinically relevant end-points and there is an urgent need to develop appropriate non-invasive surrogate endpoints to improve clinical trial design[8].

Vascular Adhesion Protein-1 (VAP-1)

The progression of PSC to scarring, cirrhosis and hepatobiliary cancer is driven by a chronic inflammatory response and immune cell mediated destruction of bile ducts[9]. Our research implicates VAP-1 in the inflammation that drives fibrogenesis in liver disease[10]. VAP-1 also acts as an adhesion receptor to support leucocyte recruitment in liver inflammation, a

For peer review only

function that is critical in animal models in the formation of fibrosis[11, 12]. Thus, inhibition of VAP-1 is expected to impact both inflammation and fibrosis, indeed treatment with an antibody against VAP-1 prevents fibrosis in murine models of liver injury[11]. Data also shows particularly high levels of circulating serum VAP-1 (sVAP-1) in patients with PSC as well as a strong correlation between sVAP-1/semicarbazide-sensitive amine oxidase (SSAO) activity in serum and histological fibrosis scores in patients with fatty liver disease[11]. Based on the strong up-regulation of hepatic VAP-1 reported in PSC patients[11], we hypothesise that levels of sVAP-1/SSAO will correlate with the severity of fibrosis in PSC and predict patients at risk of progressive disease.

These observations underpin our proposal that VAP-1 has an important role in the progression of liver fibrosis. We now plan to test the hypothesis that inhibiting VAP-1 with a neutralising antibody (BTT1023) will reverse or delay fibrogenesis in patients with PSC. Additionally, reliable biomarkers that correlate with fibrosis stage and progression of liver disease are in demand in order to predict outcome and to stage disease, without the need for invasive liver biopsy. This research will allow us to translate laboratory research into a proof of activity clinical trial that will elucidate the role of VAP-1 in liver fibrosis and its potential as a therapeutic target and biomarker.

BTT1023 (now known as Timolumab)

BTT1023 is a fully human, monoclonal, anti-VAP-1 antibody which blocks the adhesion function of VAP-1, thereby diminishing leukocyte entry into sites of tissue inflammation. Going forward BTT1023 will be known as Timolumab, however the original numeric name is used in this manuscript in order to remain in keeping with the trial protocol. In vivo,

For peer review only

blocking VAP-1 function with an anti-mouse anti-VAP-1 antibody significantly alleviates inflammation in mouse models of arthritis and liver fibrosis[11,13,]. BTT1023 appears to be safe and well tolerated in humans: BTT1023 has been given in doses up to 8 mg/kg in patients with rheumatoid arthritis and psoriasis after oral premedication (cetirizine & ibuprofen) and also appears safe and well-tolerated in repeated intravenous dosing[14]. No cytokine release syndrome was seen. This dose therefore appears reasonable as a starting point and is considered to provide appropriate bridging into existing clinical data.

In the well tolerated arthritis study, patients received 5 doses at fortnightly intervals with potentially efficacious trough levels being achieved by Day 42, after 3 doses. The psoriasis study used a more rapid loading of 3 doses at days 1, 8 and 22, which was also well tolerated. Therefore, we have decided to deploy the accelerated dosing scheme (i.e. dosing on days 1, 8, and biweekly thereafter) in the current study. [14]

BTT1023 is produced in a Chinese hamster ovary cell culture and purified with appropriate methods including specific viral inactivation and removal procedures. BTT1023 is contained in single-use 100 mg/10 mL glass vials and requires dilution with BTT1023 IV Infusion Diluent (0.9% sodium chloride and 0.02% polysorbate 80 in water for injection) prior to administration by intravenous infusion. The amount of diluent to be added to the BTT1023 concentrate is calculated to consistently provide a total diluted drug product infusion volume of 50 mL. BTT1023 and its diluent are stored at 2-8°C and the maximum shelf life for the diluted infusion solution is 24 hrs when stored at this temperature.

For peer review only - http://bmjopen.bmj.com/site/about/guidelines.xhtml

Objectives

The trial primary objective is to determine the activity of the anti-VAP-1 antibody BTT1023 in patients with PSC as measured by a decrease in alkaline phosphatase levels (ALP) (primary endpoint) with secondary endpoints to include various measures of liver injury and fibrosis and evaluation of the safety, effective dose, and tolerability of BTT1023 in patients with PSC. Our secondary objectives include; determining the mechanisms of action of BTT1023 through in vitro assessment of sVAP-1 concentration, SSAO enzyme activity and immune cell function; evaluating the potential of a novel MRI based assessment of liver fibrosis and biliary strictures for assessing therapeutic response in PSC; and assessment of the use of sVAP-1/SSAO as a biomarker to monitor disease progression in PSC.

For peer review only

METHODS

Study design overview

BUTEO is a two-stage single-arm, open-label, multi-centre hybrid trial of treatment with monoclonal anti-VAP-1 antibody, BTT1023 in adult patients with PSC. The sample size will be a maximum of 59 patients, who will each have up to 7 intravenous infusions of BTT1023 over a 78 day treatment period. All patients will be followed up until Day 120 (42 days after the last administration of treatment). At specified time points during each visit, serum will be taken for circulating levels of BTT1023 as well as for anti-drug antibodies, VAP-1 activity, and additional exploratory research samples. This includes pre, during and post-dose, with some patients also attending 24 hours later for further blood testing.

The trial is comprised of two components. The run-in component of the trial incorporates a conventional 3+3 cohort design to confirm the therapeutic dose, with decisions regarding continuation based on toxicity and pharmacokinetic (PK) data, Figure 1. The trial begins with the recruitment of 6 patients all receiving the starting dose of 8 mg/kg. Recruitment will be paused while awaiting the results of trough blood serum levels of circulating BTT1023 at Visit 7 (Day 50) from all 6 patients and until the Dose Limiting Toxicity (DLT) reporting period is completed for each patient (Visit 10 [Day 99]).

If results from the first cohorts show an acceptable DLT rate (see later) and trough levels of BTT1023 meet the stipulated success criteria, the trial will continue into the expansion component of the trial. Acceptable trough levels have been set a trough concentration of 3 µg/mL free circulating BTT1023 at 8 weeks from first infusion, which is approximately 100 fold the dissociation constant (Kd) of BTT1023 from VAP-1 and will result in target

For peer review only

occupancy of approximately 90%. In the event that DLT rate is acceptable but the PK levels do not meet the success criteria then the trial moves into a conventional 3+3 cohort design, using escalating doses of BTT1023. In this event, the original 6 patient cohort will no longer be evaluated, but a new cohort of 3 patients will be recruited to receive the newly identified test dose of 12 mg/kg. If there are no DLTs at Visit 10 [Day 99], an additional cohort of 3 patients will be recruited at the new test dose. If the DLT rate remains acceptable but the PK levels still do not meet the success criteria, a further 3+3 patients will be recruited at the highest dose of 16mg/kg. If this is found not to result in sufficient blood levels of BTT1023 then the trial will be stopped. If the PK values are found to be too high (such as resulting in trough levels consistently exceeding 100 µg/mL) then there is potential to de-escalate the dose in agreement with the DMC and after regulatory approvals. The trial will be stopped at any stage where patient safety is compromised. Individual patients will only receive one dose level. Once a confirmed dose has been established, the trial will expand until a total of 37 patients have received treatment with the confirmed dose, including those patients who have previously received this dose during the confirmatory period. Those patients not receiving the confirmed dose will not be included in the final evaluation.

Patient selection

Patients with particularly elevated ALP levels are predicted to be more at risk of progressive disease; thus these patients will be selected. Currently four UK academic hospital centres are involved (based in Birmingham, Nottingham, Oxford and Newcastle) with further centres potentially coming on board shortly. Informed consent will be obtained by appropriately trained members of the research team at each site.

For peer review only

The main inclusion criteria are patients aged 18 to 75 with a clinical diagnosis of PSC, as evidenced by chronic cholestasis of more than six months duration with either an MRI or liver biopsy consistent with PSC and in the absence of a documented alternative aetiology. Patients must have an ALP of at least 1.5 times the upper limit of normal. Those with concomitant inflammatory bowel disease (IBD) must have evidence to show clinically and colonoscopically stable disease within the past 12 months, without findings of high grade dysplasia and without the need for biological therapies. In those on treatment with ursodeoxycholic acid (UDCA), therapy must be stable for at least 8 weeks prior to screening and at a dose not greater than 20 mg/kg/day. In those not on treatment with UDCA at the time of screening, a minimum of 8 weeks since the last dose of UDCA should be recorded. As an IgG molecule, BTT1023 is not anticipated to interfere with chromosomal material however teratogenicity has not been studied and so rigorous steps will be taken to exclude pregnancy pre-treatment and with regards to need for effective contraception throughout the trial and for 99 days after. Some of the main exclusion criteria can be seen in Box 1.

TREATMENT

A single arm, rather than placebo controlled design, was chosen to allow efficient enrolment of patients into the study due to its intensive nature, in which a significant chance of being allocated into the placebo group may act as a substantial barrier for enrolment. The proposed primary endpoint (ALP) is a biochemical measurement and thus not open to subjective bias as a clinical assessment would be, rendering the necessity of a concurrent control group less vital.

For peer review only

TRIAL SCHEMA

Given the unpredictable nature of PSC and natural variation of ALP levels, we have built into the trial a two stage screening process over 4-7 weeks, whereby the ALP levels must not vary by more than 25% in order to continue to enrolment. During screening, patients have routine blood screens and other non-invasive markers of liver fibrosis including Mayo PSC Risk Score, MELD, Enhanced Liver Fibrosis (ELF), Fibroscan and MRCP with additional LiverMultiscan® imaging. These will then be repeated during treatment and in the follow-up period to assess for any change.

During all 7 treatment visits, patients will receive pre-medication with cetirizine 10 mg + ibuprofen 400 mg orally (in the absence of any contra-indications) plus intravenous hydrocortisone 100 mg, 1-2 hrs pre infusion (the latter for the first 3 doses only). The first infusion will be given over 2 hours, with a 4 hour monitoring period post-infusion. Provided no adverse reactions are seen, the infusion time will drop to 1 hour with initially a 3 hour observation period (for the second dose) and then down to 2 hours monitoring post-infusion (for all subsequent doses). Safety investigations completed pre and post-infusion include haematology/biochemistry sampling, along with ECG, clinical assessment and physical examination. The full trial schema can be seen as Figure 2.

A 0.5-1.0 mL aliquot of the BTT1023 infusion solution will be taken at the end of every infusion and refrigerated. These samples may be used for analysis of BTT1023 concentration if anomalies in PK data are observed that could be due to errors in IMP preparation.

For peer review only

Data analysis plan:

Power calculations & Sample size justification

The dose confirmatory stage requires a maximum of 18 patients. The sample size has been based on the classic 3+3 design, investigating three fixed dose increments (8, 12 and 16 mg/kg), with no dose skipping.

The expansion phase follows a Simon's 2-stage minimax design with lower and upper acceptability bounds of 15 and 30% respectively and error rates α=0.10 and β=0.20. Thirty-seven patients are required in this stage of the trial, however, to account for patient dropout, estimated at approximately 10%, the sample size is extended by a further 4 patients. As such the target recruitment is up to 41 patients for the expansion phase, and up to 59 participants in total. An interim assessment will examine the primary outcome once 18 evaluable patients have been recruited at the confirmed dose. If 3 or more responses are observed in stage 1, then the trial will continue into stage 2. Recruitment will not be halted while stage 1 is assessed. If the stage 1 criterion is not met then the trial will cease. However if the criterion is met, then further patient recruitment continues until 37 evaluable patients are recruited. If overall there are 9 or more responses from 37 evaluable patients then we conclude that the treatment warrants further investigation.

In this setting the interpretation of alpha (α) is the probability satisfying stage-1 and observing 9 or more responses in 37 patients overall when the true response rate is 15%; a false positive result (Type I error). Beta (β) is the probability of failing to acknowledge activity when the true response rate is 30% (Type II error). As such the power, 1- β, is the probability of taking an effective treatment forward.

For peer review only

OUTCOME MEASURES & ANALYSIS

This is an early phase trial of BTT1023 in immune mediated liver disease, with the rationale to identify biochemical efficacy (reduction in ALP) and safety, in an orphan disease that presently lacks any other effective medical therapy. The trial design therefore focuses on identifying early biochemical efficacy signals to justify larger scale, randomised controlled trials of a longer duration.

Our primary outcome measure is patient response to treatment at Day 99, and is a reduction in serum ALP by 25% or more from baseline to Day 99. Our data on stability of ALP in PSC suggests that such responses occur very seldom during the natural course of the disease, and we can therefore reliably assess changes from baseline and response rates for this proof of concept study to evaluate the therapeutic potential of BTT1023. In addition we are excluding patients in whom the levels change significantly naturally by >25% in the 5-7 week screening period.

Secondary outcome measures include safety and tolerability (via treatment compliance, patient withdrawal, frequency of SAE/AEs, change in quality of life questionnaires: EQ-5D, Fatigue Severity Scale, Pruritus Visual Analogue Score, inflammatory bowel disease diaries), and of BTT1023 efficacy (via tests of liver fibrosis including ELF, Fibroscan and liver biochemistry). Additionally liver MRI is an emerging method for monitoring liver disease and its treatment. We will evaluate changes in MRI imaging pre and post therapy using the LiverMultiscan® protocol (or equivalent methodology, at sites where this is possible). Finally we will evaluate changes in sVAP-1/SSAO as a biomarker of liver disease activity across the study period.

For peer review only

INTERIM ANALYSIS

An interim analysis will be completed once 18 patients have been evaluated for the primary outcome (ALP response). If 3 or more responses are observed then the trial will continue. If this is not met then the trial will cease. If adequate response is seen, a further 19 patients will be recruited in order to obtain the required sample size of 37 patients – allowing for 10% patient drop out during trial duration, this number could reach a total of 41 patients recruited. The final success criterion chosen maintains power, and in doing so can incur an increased type-1 error rates (note that the stage-1 criterion is fixed since the final sample size is unknown at stage-1). These have been calculated by the trial Biostatistician and independently verified by a CRCTU Biostatistician.

Patients that cannot be evaluated for the primary outcome (e.g. due to withdrawal or lost to follow-up) will be treated as non-responders.

Final analyses

Final analyses of the primary outcome will be performed when all patients have been followed to Day 120 and once the database has been locked. If overall there are nine or more responses from 37 evaluable patients then we conclude that the treatment warrants further investigation. Only patients treated at the confirmed dose will contribute to the total patient requirement.

For peer review only

CONDUCT OF THE TRIAL

Ethical & regulatory approvals

The trial is being performed in accordance with the recommendations guiding physicians in biomedical research involving human subjects, adopted by the 48th World Medical Association General Assembly[15] as well as the Research Governance Framework for Health and Social Care, the applicable UK Statutory Instruments, (which include the Medicines for Human Use Clinical Trials 2004 and subsequent amendments and the Data Protection Act 1998) and Guidelines for Good Clinical Practice. The protocol has been approved by the Research Ethics Committee (REC) with the reference of; 14/EM/1272. The first REC approval date was 06/01/2015 with subsequent amendments on 18/3/2015 (non-substantial), 27/11/2015 (substantial) and 16/3/2016 (substantial) also now approved. All active sites have obtained local Research & Development department approval and are up-to-date with the latest protocol amendment.

Standard regulations for data handling and patient confidentiality are being followed, in accordance with the 1998 UK Data Protection Act. During the trial, patients will be identified using only their unique trial number, initials and date of birth on the Case Report Form (CRF) and in correspondence between the Trials Office and the participating site. Data quality will be maintained according to recognised guidance and will be consistent to the source data. All essential trial documentation and source records will be securely retained for at least 15 years after the end of the trial or following the processing of all biological material collected for research, whichever is the latter.

For peer review only

Sponsorship & Indemnity

This is a clinician-initiated and clinician-led trial funded by the National Institute for Health Research (NIHR) which receives funds directly from the Department of Health (DoH). Biotie Therapies will be supplying BTT1023 free of charge to all individual NHS trusts that will be directly treating patients as part of this clinical trial. There are payments to individual NHS trusts on a per-patient basis, to cover trial running costs. The University of Birmingham are acting as trial sponsor and as such remains responsible for the study conduct. University of Birmingham employees are indemnified by the University insurers for negligent harm caused by the design or co-ordination of the clinical trials they undertake whilst in the University's employment.

ADVERSE EVENT REPORTING

Dose Limiting Toxicity

Dose Limiting Toxicity (DLT) is defined as an Adverse Event (AE) that meets the criteria of grade 3 cytokine release syndrome or grade 4/5 for any criteria, as defined in the Common Terminology Criteria for Adverse Events (CTCAE V4.0). Although previous studies have shown no DLTs with BTT1023, toxicity monitoring will be ongoing throughout the trial and any concerns with be reported to the Trials office within 24 hours of the investigator becoming aware of the event. The DLT reporting period is from Visit 3 (first infusion) to Visit 10 (21 days after last infusion). An acceptable DLT rate has been established for the trial as a maximum of 1 incidence in 6 patients (~17%). If the DLT rate rises to 2 or more, at any stage during the DLT reporting period, (Visit 3 [Day 1] to Visit 10 [Day 99]) the trial will be halted in consultation with the DMC.

For peer review only

AEs/SAEs

The collection and reporting of Adverse Events (AEs) will be in accordance with the Medicines for Human Use Clinical Trials Regulations 2004 and its subsequent amendments. The CTCAE version 4.0 criteria will be used to grade each AE. Any pre-existing conditions will be reported in the medical history, and will not be reported as an AE unless the condition worsens by at least one CTC grade during the trial. The reporting period for AEs will commence from the date of consent (Visit 1) and will continue until the final follow-up visit (Visit 11: Day 120), or alternatively up to 45 days post last infusion if the patient withdraws from the study prior to completion of all 7 study drug infusions. All trial patients will continue to receive standard concomitant clinical care throughout the study.

The Sponsor, appropriate regulatory authority (e.g. Medicines and Healthcare Products Regulatory Agency (MHRA)) and the Research Ethics Committee (REC) will be informed of all SAEs as required by current regulations. All SAEs will be reported within 24 hours of the investigator becoming aware of the event and will be judged independently by a Clinical Coordinator in terms of seriousness and causality. An SAE judging to have a reasonable causal relationship to the drug will be recorded as a Serious Adverse Reaction (SAR) or Suspected Unexpected Serious Adverse Reaction (SUSAR), as appropriate. The independent Data Monitoring Committee (DMC) will also review all SAEs.

All individual events categorised as a fatal or life threatening SUSAR will be reported by the BUTEO Trials Office to the MHRA and REC within 7 days and all events categorised as Unexpected and Related SAEs to the REC within 15 days. All such events will also be

For peer review only - http://bmjopen.bmj.com/site/about/guidelines.xhtml

reported concurrently to Biotie Therapies' nominated safety representative. In the event that a patient or their partner becomes pregnant during the SAE reporting period this will be recorded and reported and followed up, subject to required patient/partner approvals.

MONITORING

Independent onsite monitoring will be carried out as required following an initial site-specific risk assessment. Additional on-site monitoring visits will be triggered for example by poor CRF return, poor data quality, low SAE reporting rates, excessive number of patient withdrawals or deviations. Central Trials Office staff will be in regular contact with the site research team to check on progress and address any queries that they may have. Trials staff will check incoming CRFs for compliance with the protocol, data consistency, missing data and timing. Sites may be suspended from further recruitment in the event of serious and persistent non-compliance with the protocol and/or GCP, and/or poor recruitment. Any major problems identified during monitoring may be reported to the Trial Management Group and the Trial Steering Committee and the relevant regulatory bodies.

For peer review only

DISCUSSION

PSC is a prime example of a progressive inflammatory liver disease characterised by relentless liver fibrosis. There is a high unmet need for new therapies as no currently licensed therapy has been shown to alter the natural course of the disease. The progression of PSC to scarring, cirrhosis and hepatobiliary cancer is driven by a chronic inflammatory response and immune cell mediated destruction of bile ducts. Our research suggests that VAP-1 is heavily implicated as a key driver for fibrogenesis and as such, provides a target for the possible slowing or even reversal of the liver damage seen in PSC.

The unpredictability of PSC along with its designation as a rare orphan disease pose particular challenges in trial design. ALP fluctuates during the natural course of the disease which limits its usefulness as a primary endpoint, however it is commonly used as a standard marker of PSC disease activity, in the absence of a viable alternative. Thus, reliable biomarkers that correlate with fibrosis stage and progression of liver disease are in demand in order to predict outcome and to stage disease without the need for invasive liver biopsy, and this trial will aid in investigating the role of VAP-1 in liver fibrosis and its potential as a therapeutic target and biomarker.

This unique trial design, incorporating a dose confirmatory and safety stage (based on the traditional 3+3 design), then followed by a Phase II Simon's two-stage design is aimed to determine not only a safe and well tolerated dose of BTT1023, but also the efficacy of this treatment in a new disease group.

For peer review only

Screening for this study commenced on 01/02/2015 with the first patient registered on 10/09/2015. Recruitment is ongoing.

For peer review only

Acknowledgements:

This project is funded by the Efficacy and Mechanism Evaluation (EME) Programme*, an MRC and NIHR partnership. The authors would like to thank the NIHR, the NIHR Liver BRU in Birmingham, Biotie Therapies Corp, the Queen Elizabeth Hospital Liver PPI group and the patients involved. *The EME Programme is funded by the MRC and NIHR, with contributions from the CSO in Scotland and NISCHR in Wales and the HSC R&D Division, Public Health Agency in Northern Ireland.

Authors' contributions: All authors have read and approved the final manuscript. KA is lead sub-investigator at the Birmingham BUTEO site and wrote this manuscript in conjunction with GMH. CW, MC, AR, MW, RF, AK, DHA, DB and GMH were involved in the protocol development. RF & AK also contribute statistical analysis. DHA was responsible for the original concept.

Funding & NIHR disclaimer: This work is supported by the NIHR Efficacy and Mechanism Evaluation (EME) Programme, grant number 12/165/31. The full title is: "Targeting vascular adhesion protein-1 (VAP-1) for the treatment of liver fibrosis: a study of efficacy and mechanisms in patients with primary sclerosing cholangitis (PSC)". This article represents independent research funded by the National Institute for Health Research (NIHR) Birmingham Liver Biomedical Research Unit (BRU). The views expressed in this publication are those of the author(s) and not necessarily those of the MRC, NHS, NIHR or the Department of Health.

For peer review only

Competing interests: CW and DHA report grants from NIHR MRC, during the conduct of the study; grants and non-financial support from Pharmaxis Inc, Australia, outside the submitted work; and have a patent "The use of VAP-1 inhibitors for treating fibrotic conditions (WO 2011029996 A1)" issued. Other authors have no competing interests declared.

For peer review only

**Box 1: Main Exclusion criteria (not a complete list)**
Subjects must **NOT** meet any of the following:-

- AST and ALT >10 x ULN or bilirubin >3xULN or INR >1.3 in the absence of anti-coagulants.
- Serum creatinine >130 µmol/L or platelet count <50 x 10^9/L.
- Any evidence of hepatic decompensation past or present, including ascites, hepatic encephalopathy or variceal bleeding.
- Recent cholangitis within last 90 days or ongoing need for prophylactic antibiotics.
- Pregnancy or breast feeding.
- Flare in colitis activity within last 90 days requiring intensification of therapy beyond baseline maintenance treatment; use of oral prednisolone >10 mg/day, biologics (i.e. monoclonal antibodies) and or hospitalisation for colitis within 90 days. Prior use of biologics is not a contraindication to screening.
- Diagnosed cholangiocarcinoma or high clinical suspicion of cholangiocarcinoma.
- Concurrent malignancies or invasive cancers diagnosed within past 3 years except for adequately treated basal cell and squamous cell carcinoma of the skin and in situ carcinoma of the uterine cervix.
- Presence of a percutaneous drain, bile duct stent or prior organ transplantation.
- Participation in an investigational trial of a drug or device within 60 days of screening or 5 half-lives of the last dose of investigational drug.
- Positive screening test for tuberculosis (TB) (including T-SPOT.TB TB test), unless respiratory review confirms false positive test results.
- Receipt of live vaccination within 6 weeks prior to baseline visit.

For peer review only

References:

[1] Lindkvist B, Benito de Valle M, Gullberg B, Bjornsson E. Incidence and prevalence of primary sclerosing cholangitis in a defined adult population in Sweden. Hepatology. 2010 Aug;52(2):571–7.

[2] Bambha K, Kim WR, Talwalkar J, Torgerson H et al. Incidence, clinical spectrum, and outcomes of primary sclerosing cholangitis in a United States community. Gastroenterology. 2003 Nov;125(5):1364–9.

[3] Molodecky NA, Kareemi H, Parab R, Barkema HW et al. Incidence of primary sclerosing cholangitis: a systematic review and meta-analysis. Hepatology. 2011 May;53(5):1590–9. doi: 10.1002/hep.24247.

[4]Bergquist A, Said K, Broomé U. Changes over a 20-year period in the clinical presentation of primary sclerosing cholangitis in Sweden. Scand J Gastroenterol. 2007 Jan;42(1):88-93.

[5] Loftus EV, Sandborn WJ, Lindor KD, LaRusso NF. Interactions between chronic liver disease and inflammatory bowel disease. Inflamm Bowel Dis 1997;3:288–302.

[6] Boonstra K, Weersma RK, van Erpecum KJ, Rauws EA et al. Population-based epidemiology, malignancy risk, and outcome of primary sclerosing cholangitis. Hepatology 2013 Dec;58(6):2045-2055.

[7] Broome U, Olsson R, Loof L, Bodemar G et al. Natural history and prognostic factors in 305 Swedish patients with primary sclerosing cholangitis. Gut 1996 Apr;38(4):610-615.

[8] Ponsioen CY, Chapman RW, Chazouillères O, Hirschfield GM et al. Surrogate endpoints for clinical trials in primary sclerosing cholangitis: Review and results from an International PSC Study Group consensus process. Hepatology. 2016 Apr;63(4):1357-67.

For peer review only

[9] Hirschfield GM, Karlsen TH, Lindor KD, Adams DH. Primary sclerosing cholangitis. Lancet 2013 Nov 9 382(9904): 1587-1599.

[10] Lalor PF, Edwards S, McNab G, Salmi M et al. Vascular Adhesion Protein-1 mediates adhesion and transmigration of lymphocytes on human hepatic endothelial cells. J Immunol. 2002 Jul 15;169(2): 983-992.

[11] Weston CJ, Shepherd EK, Claridge LC, Rantakari P et al. Vascular adhesion protein-1 promotes liver inflammation and drives hepatic fibrosis. J Clin Invest. 2015 Feb 2; 125(2): 501–520.

[12] Hsia LT, Ashley N, Ouaret D, Wang LM et al. Myofibroblasts are distinguished from activated skin fibroblasts by the expression of AOC3 and other associated markers. Proc Natl Acad Sci U S A. 2016 Apr 12;113(15):E2162-71.

[13] Marttila-Ichihara F, Smith DJ, Stolen C, Yegutkin GG et al. Vascular amine oxidases are needed for leukocyte extravasation into inflamed joints in vivo. Arthritis Rheum. 2006 Sep;54(9):2852-62.

[14] Biotie Therapies Corp, (2010). BTT12-CD015: A multiple ascending dose study to assess the safety and pharmacokinetics of repeated intravenous doses of BTT1023 in patients with rheumatoid arthritis - a double-blind randomized placebo-controlled sequential group trial

[15] WMA Declaration of Helsinki - Ethical Principles for Medical Research Involving Human Subjects. Available from http://www.wma.net/en/30publications/10policies/b3/index.html (accessed 01/07/2016).

For peer review only

Figure 1. Flow diagram showing the trial decision guidelines for the run-in period of the trial design incorporating a conventional 3+3 cohort design if dose escalation is required

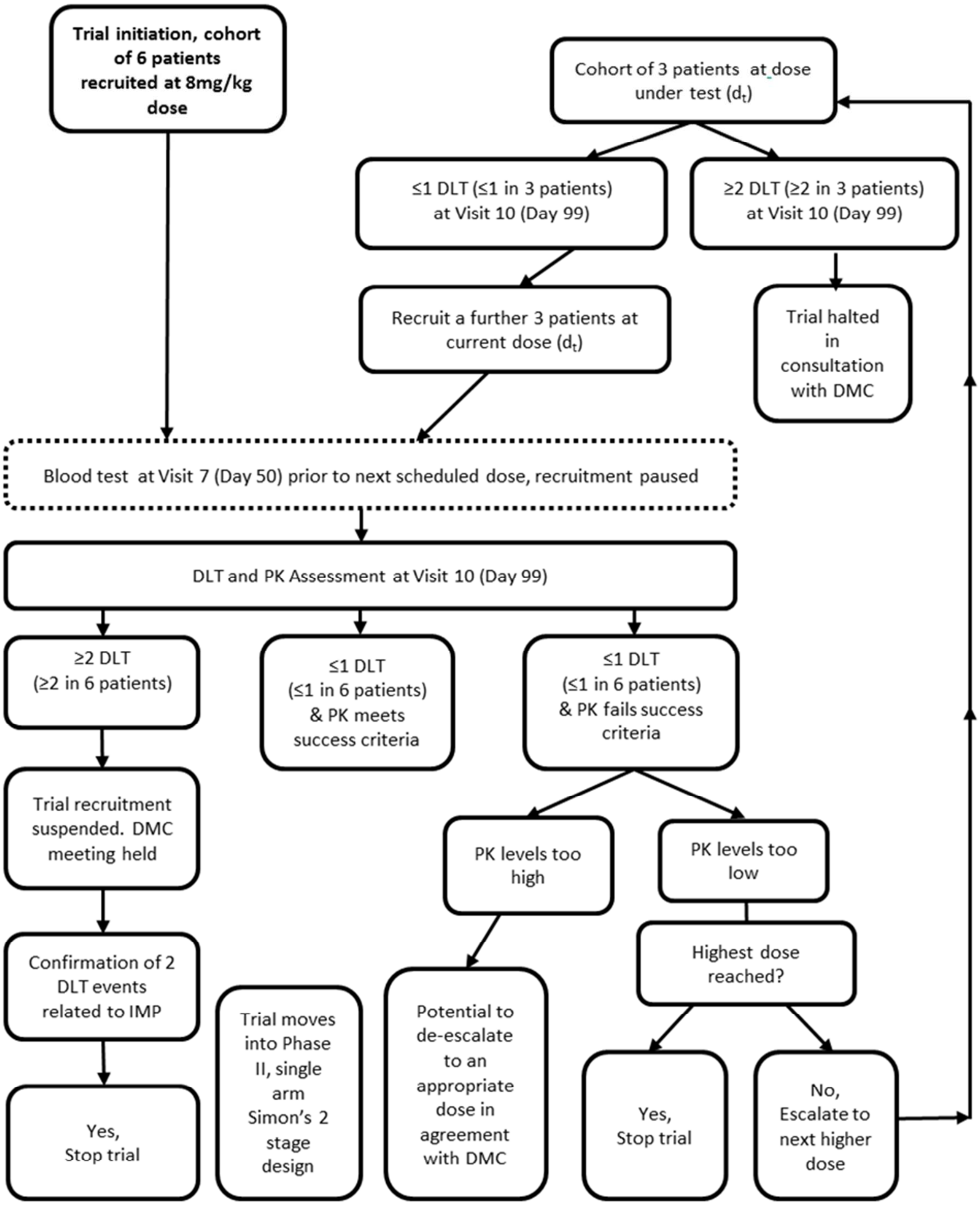

Figure 2. BUTEO Trial Schema

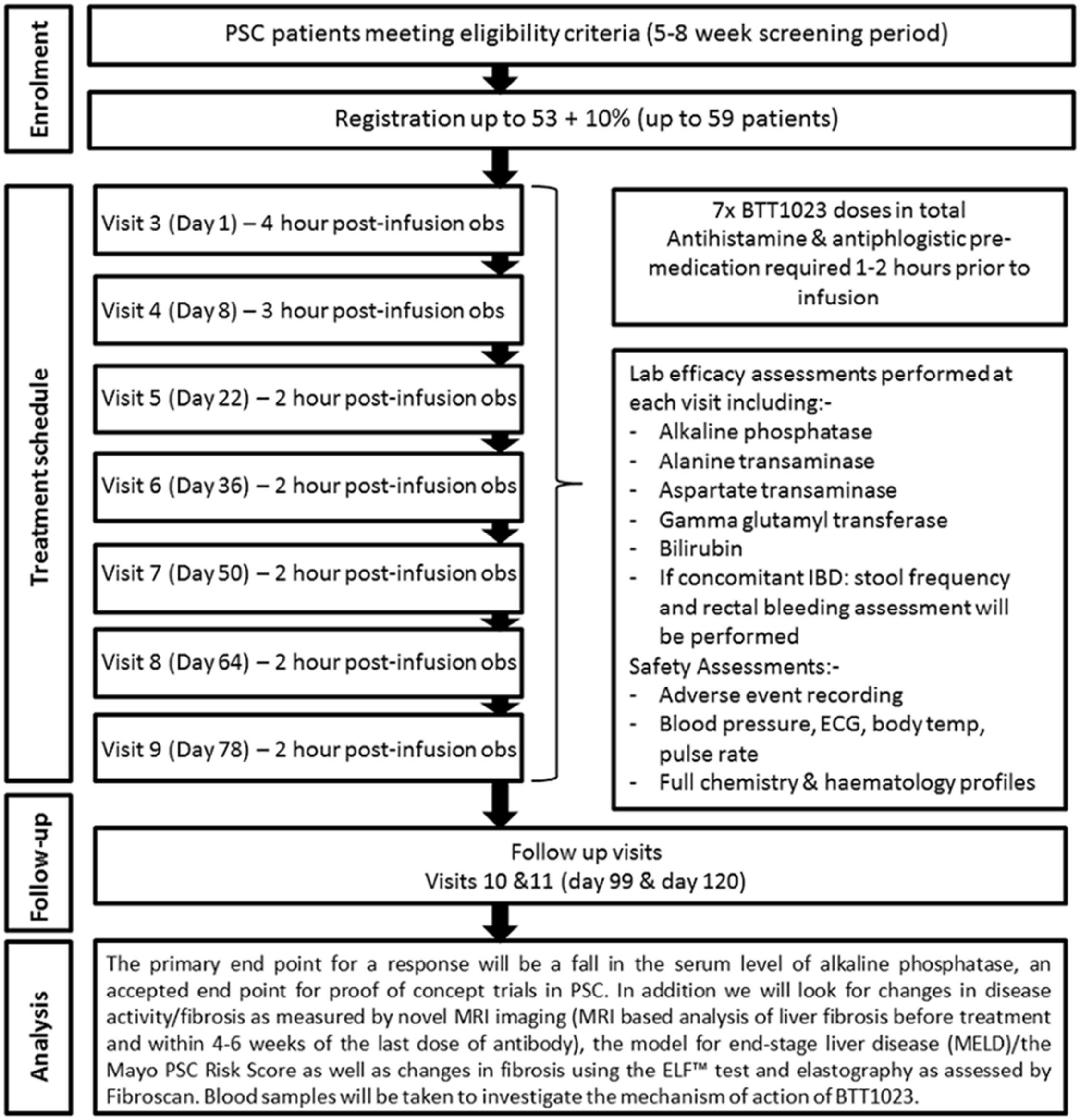

BUTEO

SPIRIT

STANDARD PROTOCOL ITEMS: RECOMMENDATIONS FOR INTERVENTIONAL TRIALS

SPIRIT 2013 Checklist: Recommended items to address in a clinical trial protocol and related documents*

Section/item	Item No	Description	PAGE
**Administrative information**			
Title	1	Descriptive title identifying the study design, population, interventions, and, if applicable, trial acronym	2
Trial registration	2a	Trial identifier and registry name. If not yet registered, name of intended registry	4
	2b	All items from the World Health Organization Trial Registration Data Set	2,4,9-12, 18,22-25
Protocol version	3	Date and version identifier	4
Funding	4	Sources and types of financial, material, and other support	23
Roles and responsibilities	5a	Names, affiliations, and roles of protocol contributors	2,23
	5b	Name and contact information for the trial sponsor	2
	5c	Role of study sponsor and funders, if any, in study design; collection, management, analysis, and interpretation of data; writing of the report; and the decision to submit the report for publication, including whether they will have ultimate authority over any of these activities	23
	5d	Composition, roles, and responsibilities of the coordinating centre, steering committee, endpoint adjudication committee, data management team, and other individuals or groups overseeing the trial, if applicable (see Item 21a for data monitoring committee)	20
**Introduction**			
Background and rationale	6a	Description of research question and justification for undertaking the trial, including summary of relevant studies (published and unpublished) examining benefits and harms for each intervention	6-8
	6b	Explanation for choice of comparators	12
Objectives	7	Specific objectives or hypotheses	9
Trial design	8	Description of trial design including type of trial (eg, parallel group, crossover, factorial, single group), allocation ratio, and framework (eg, superiority, equivalence, noninferiority, exploratory)	10

			PAGE
**Methods: Participants, interventions, and outcomes**			
Study setting	9	Description of study settings (eg, community clinic, academic hospital) and list of countries where data will be collected. Reference to where list of study sites can be obtained	11
Eligibility criteria	10	Inclusion and exclusion criteria for participants. If applicable, eligibility criteria for study centres and individuals who will perform the interventions (eg, surgeons, psychotherapists)	12, 25
Interventions	11a	Interventions for each group with sufficient detail to allow replication, including how and when they will be administered	10, 13
	11b	Criteria for discontinuing or modifying allocated interventions for a given trial participant (eg, drug dose change in response to harms, participant request, or improving/worsening disease)	10, 15, 18-19, 28
	11c	Strategies to improve adherence to intervention protocols, and any procedures for monitoring adherence (eg, drug tablet return, laboratory tests)	20
	11d	Relevant concomitant care and interventions that are permitted or prohibited during the trial	19
Outcomes	12	Primary, secondary, and other outcomes, including the specific measurement variable (eg, systolic blood pressure), analysis metric (eg, change from baseline, final value, time to event), method of aggregation (eg, median, proportion), and time point for each outcome. Explanation of the clinical relevance of chosen efficacy and harm outcomes is strongly recommended	15
Participant timeline	13	Time schedule of enrolment, interventions (including any run-ins and washouts), assessments, and visits for participants. A schematic diagram is highly recommended (see Figure)	10, 13, 29
Sample size	14	Estimated number of participants needed to achieve study objectives and how it was determined, including clinical and statistical assumptions supporting any sample size calculations	14, 16
Recruitment	15	Strategies for achieving adequate participant enrolment to reach target sample size	11
**Methods: Assignment of interventions (for controlled trials)**			
Allocation:			
Sequence generation	16a	Method of generating the allocation sequence (eg, computer-generated random numbers), and list of any factors for stratification. To reduce predictability of a random sequence, details of any planned restriction (eg, blocking) should be provided in a separate document that is unavailable to those who enrol participants or assign interventions	12

			PAGE
Allocation concealment mechanism	16b	Mechanism of implementing the allocation sequence (eg, central telephone; sequentially numbered, opaque, sealed envelopes), describing any steps to conceal the sequence until interventions are assigned	12
Implementation	16c	Who will generate the allocation sequence, who will enrol participants, and who will assign participants to interventions	12
Blinding (masking)	17a	Who will be blinded after assignment to interventions (eg, trial participants, care providers, outcome assessors, data analysts), and how	12
	17b	If blinded, circumstances under which unblinding is permissible, and procedure for revealing a participant's allocated intervention during the trial	12
**Methods: Data collection, management, and analysis**			
Data collection methods	18a	Plans for assessment and collection of outcome, baseline, and other trial data, including any related processes to promote data quality (eg, duplicate measurements, training of assessors) and a description of study instruments (eg, questionnaires, laboratory tests) along with their reliability and validity, if known. Reference to where data collection forms can be found, if not in the protocol	17, 20
	18b	Plans to promote participant retention and complete follow-up, including list of any outcome data to be collected for participants who discontinue or deviate from intervention protocols	16, 19
Data management	19	Plans for data entry, coding, security, and storage, including any related processes to promote data quality (eg, double data entry; range checks for data values). Reference to where details of data management procedures can be found, if not in the protocol	17
Statistical methods	20a	Statistical methods for analysing primary and secondary outcomes. Reference to where other details of the statistical analysis plan can be found, if not in the protocol	14
	20b	Methods for any additional analyses (eg, subgroup and adjusted analyses)	16
	20c	Definition of analysis population relating to protocol non-adherence (eg, as randomised analysis), and any statistical methods to handle missing data (eg, multiple imputation)	16, 19
**Methods: Monitoring**			
Data monitoring	21a	Composition of data monitoring committee (DMC); summary of its role and reporting structure; statement of whether it is independent from the sponsor and competing interests; and reference to where further details about its charter can be found, if not in the protocol. Alternatively, an explanation of why a DMC is not needed	19

			PAGE
	21b	Description of any interim analyses and stopping guidelines, including who will have access to these interim results and make the final decision to terminate the trial	16
Harms	22	Plans for collecting, assessing, reporting, and managing solicited and spontaneously reported adverse events and other unintended effects of trial interventions or trial conduct	19
Auditing	23	Frequency and procedures for auditing trial conduct, if any, and whether the process will be independent from investigators and the sponsor	20
**Ethics and dissemination**			
Research ethics approval	24	Plans for seeking research ethics committee/institutional review board (REC/IRB) approval	4,17
Protocol amendments	25	Plans for communicating important protocol modifications (eg, changes to eligibility criteria, outcomes, analyses) to relevant parties (eg, investigators, REC/IRBs, trial participants, trial registries, journals, regulators)	17
Consent or assent	26a	Who will obtain informed consent or assent from potential trial participants or authorised surrogates, and how (see Item 32)	11
	26b	Additional consent provisions for collection and use of participant data and biological specimens in ancillary studies, if applicable	11
Confidentiality	27	How personal information about potential and enrolled participants will be collected, shared, and maintained in order to protect confidentiality before, during, and after the trial	17
Declaration of interests	28	Financial and other competing interests for principal investigators for the overall trial and each study site	24
Access to data	29	Statement of who will have access to the final trial dataset, and disclosure of contractual agreements that limit such access for investigators	16
Ancillary and post-trial care	30	Provisions, if any, for ancillary and post-trial care, and for compensation to those who suffer harm from trial participation	18
Dissemination policy	31a	Plans for investigators and sponsor to communicate trial results to participants, healthcare professionals, the public, and other relevant groups (eg, via publication, reporting in results databases, or other data sharing arrangements), including any publication restrictions	4
	31b	Authorship eligibility guidelines and any intended use of professional writers	24
	31c	Plans, if any, for granting public access to the full protocol, participant-level dataset, and statistical code	24

A single arm, two-stage, multi-centre, phase II clinical trial investigating the safety and activity of the use of BTT1023, a human monoclonal antibody targeting vascular adhesion protein (VAP-1), in the treatment of patients with primary sclerosing cholangitis (PSC)

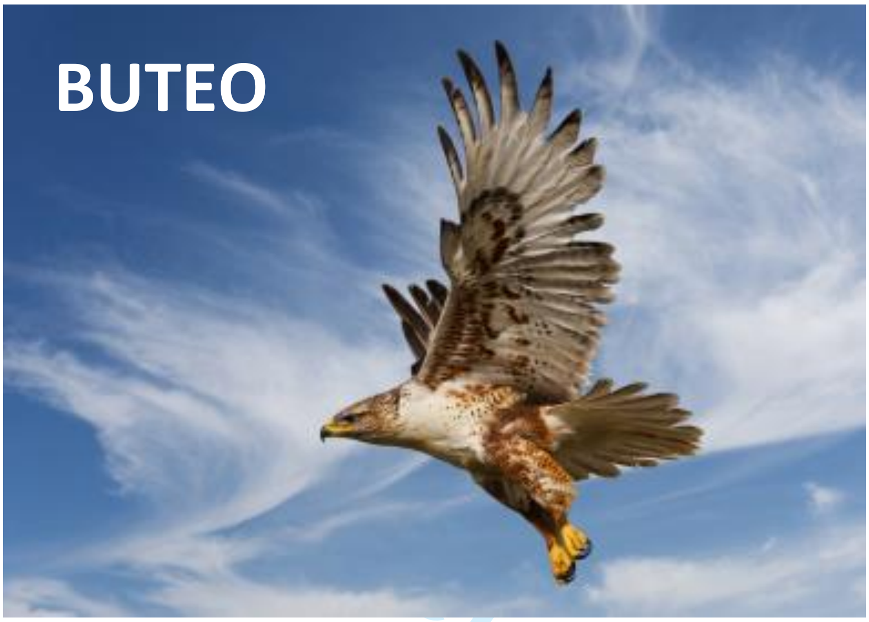

EudraCT Number: 2014-002393-37

Protocol Version: 3.0 16-Mar- 2016

Sponsor Number: RG_13-027

Clinicaltrials.gov Identifier: NCT02239211

ISRCTN Number: 11233255

CRCTU Number: HE2022

Cancer Research UK Clinical Trials Unit
@CRCTU

UNIVERSITY OF
BIRMINGHAM

For peer review only - http://bmjopen.bmj.com/site/about/guidelines.xhtml

TRIAL CONTACTS

**Sponsor:**	**University of Birmingham**
**Chief-Investigator:**	*Dr Gideon Hirschfield
**Co-Investigator:**	*Professor David Adams

* Clinical Coordinators

***Professor Philip Newsome,**

Centre of Liver Research, Institute of Biomedical Research, University of Birmingham, B15 2TT

Cancer Research UK Clinical Trials Unit

**Senior Trial Coordinator:**	Dr Anna Rowe
**Trial Coordinator:**	Amrita Athwal
**Lead Trial Statistician:**	Richard Fox
**Trial Statistician:**	Amanda Kirkham
**Pharmacy Representative:**	Amisha Desai

BUTEO Trial Office

Cancer Research UK Clinical Trials Unit (CRCTU), Institute of Cancer and Genomic Sciences, University of Birmingham, Edgbaston, Birmingham, B15 2TT

Enquiries

☎ 0121 414 4032 or 0121 414 8284

🖷 0121 414 8286

✉BUTEO@trials.bham.ac.uk

Registration – Expansion Phase Only

www.cancertrials.bham.ac.uk

Emergency registration – Expansion Phase only

☎0121 414 3366

DLT & Serious Adverse Event Reporting

🖷 0121 414 8286 (primary number)

🖷0121 414 3529 (secondary number)

☎0121 414 4032 or 0121 414 8284

For peer review only

CHIEF INVESTIGATOR SIGNATURE PAGE

BUTEO Trial Protocol Version 3.0 16th March 2016

This protocol has been approved by:

Name: Dr Gideon Hirschfield **Trial Role:** Chief Investigator

Signature: ____________________________ **Date:** DD / MON / YYYY

This protocol describes the BUTEO trial and provides information about procedures for patients taking part in the BUTEO trial. The protocol should not be used as a guide for treatment of patients not taking part in the BUTEO trial.

This protocol was written using CRCTU-PRT-QCD-001, version 1.0

For peer review only

AMENDMENTS

The following amendments and/or administrative changes have been made to this protocol since the implementation of the first approved version

Amendment number	Date of amendment	Protocol version number	Type of amendment	Summary of amendment
N/A	18th March 2015	1.0a	Non-substantial amendment	- Section 7.6.1 – wording updated to prevent additional samples being taken.<br>- Grammatical errors and line spacing errors corrected.
1	27th November 2015	2.0	Substantial	- Updated contact details throughout<br>- Formatting, typographical errors and clarifications throughout<br>- Updated and clarified Inclusion and Exclusion criteria<br>- Screening period reduced to 6-8 weeks<br>- Schedule of Events updated including:<br>▪ ELF test being included as a research sample<br>▪ central ALP for Visits 3-11<br>▪ ADA, PD and QoL not performed at Screening visit 1 & 2<br>▪ MRI scans clarified<br>- 5.2 updated to include Screening Number details<br>- 6.2 updated to clarify and include Screening Number<br>- 7.3 Visit tests corrected and updated<br>- 7.5 & 7.6 updated to account for amendment changes<br>- 7.7 Clarified<br>- 7.9 Updated regarding delay to infusion<br>- 7.11 Contraception and pregnancy section added<br>- 8.1 & 8.2 clarified<br>- 9.1 Details updated and CRF list updated<br>- References updated<br>- Appendices updated
2	16th March 2016	3.0	Substantial	- Inclusion criteria for patients ALP value has been reduced from >2 x ULN to >1.5 x ULN<br>- Pre-medications updated (7.9 and where

For peer review only - http://bmjopen.bmj.com/site/about/guidelines.xhtml

				applicable) to include hydrocortisone at Visit 3-5<br>- Section 8.1.2 "Hypersensitivity, Infusion Reactions and Infusion Related Reactions" added<br>- Grammatical and continuity errors corrected<br>- Additional telephone number for Trials Office added throughout

For peer review only

TRIAL SYNOPSIS

Title

A single arm, two-stage, multi-centre, phase II clinical trial investigating the safety and activity of the use of BTT1023, a human monoclonal antibody targeting vascular adhesion protein (VAP-1), in the treatment of patients with primary sclerosing cholangitis (PSC).

Trial Design

This is a two-stage, single-arm, open-label, multi-centre phase II trial.

Objectives

Primary:

1) To determine the activity of the anti-VAP-1 antibody BTT1023 in patients with PSC as measured by a decrease in alkaline phosphatase levels (ALP) (primary endpoint) with secondary endpoints to include various measures of liver injury and fibrosis.
2) To evaluate the safety, effective dose, and tolerability of BTT1023 in patients with PSC.

Secondary:

1) To determine the mechanisms of action of BTT1023 through in vivo assessment of VAP-1/semicarbazide-sensitive amine oxidase (SSAO) enzyme activity and immune cell function.
2) To evaluate the potential of a novel Magnetic Resonance Imaging (MRI) based assessment of liver fibrosis and biliary strictures for assessing therapeutic response in PSC.
3) Assess the use of sVAP-1/SSAO as a biomarker to monitor disease progression in PSC.

Outcome Measures

Primary:

1) Response at Day 99: a reduction in serum alkaline phosphatase (ALP) by 25% or more from baseline to Day 99.

Secondary:

1) Safety and tolerability
    - Treatment Compliance (including patient withdrawal) and Serious Adverse Event (SAE) and Adverse Event (AE) frequency.
2) Calculation of any change (*improvement or worsening*) from baseline to Day 99 in:
    - Quality of life questionnaires: EQ-5D, Fatigue Severity Scale, Pruritus Visual Analogue Score (VAS), Inflammatory Bowel Disease (IBD) Diaries (if applicable).
    - Tests of liver fibrosis: enhanced liver fibrosis (ELF) and Fibroscan.
    - Individual markers of liver biochemistry and function (aspartate transaminase [AST], alanine transaminase [ALT], ALP, gamma glutamyl transferase [GGT], bilirubin, Albumin, International Normalised Ratio [INR])and composite risk scores (Mayo PSC Risk score and model for end-stage liver disease [MELD] score).

For peer review only - http://bmjopen.bmj.com/site/about/guidelines.xhtml

- Change (*improvement or worsening* of) in LiverMultiscan® MRI imaging – Liver MRI is an emerging method for monitoring liver disease and its treatment. We will evaluate changes in MRI imaging pre and post therapy using the LiverMultiscan® protocol (or equivalent methodology, at sites where this is possible).

3) Evaluate changes in sVAP-1/SSAO as a biomarker of liver disease activity across the study period.

Patient Population

Patients who have been clinically diagnosed with PSC, as confirmed in one of the trial centres, and who also meet the inclusion and exclusion criteria.

Sample Size

The sample size is a maximum of 59 patients.

Inclusion and Exclusion Criteria

Inclusion criteria

Subjects must meet *all* of the following inclusion criteria to be eligible for participation in the clinical trial:

1. Males and females 18 - 75 years of age who are willing and able to provide informed, written consent and comply with all study requirements
2. Clinical diagnosis of PSC as evident by chronic cholestasis of more than six months duration with either a consistent MRI showing sclerosing cholangitis or a liver biopsy consistent with PSC in the absence of a documented alternative aetiology for sclerosing cholangitis
3. In those with concomitant Inflammatory Bowel Disease, clinical and colonoscopic evidence within the last year of stable disease, without findings of high grade dysplasia
4. In those on treatment with ursodeoxycholic acid (UDCA), therapy must be stable for at least 8 weeks and at a dose not greater than 20mg/kg/day. In those not on treatment with UDCA at the time of screening, a minimum of 8 weeks since the last dose of UDCA should be recorded
5. Serum ALP greater than 1.5 x upper limit of normal (ULN)
6. Stable serum ALP levels (levels must not change by more than 25% from Screening Visit 1 and Screening Visit 2)
7. Female subjects of childbearing potential must have a negative pregnancy test prior to starting study treatment. For the purposes of this study, a female subject of childbearing potential is a woman who has not had a hysterectomy, bilateral oophorectomy, or medically-documented ovarian failure. Women ≤ 50 years of age with amenorrhea of any duration will be considered to be of childbearing potential
8. All sexually active women of childbearing potential must agree to use two forms of highly effective method of contraception from the Screening Visit throughout the study period and

   for 99 days following the last dose of study drug. If using hormonal agents the same method must have been used for at least 1 month before study dosing and subjects must use a barrier method as the other form of contraception. Lactating women must agree to discontinue breast feeding before study investigational medicinal product administration

For peer review only

9. Men, if not vasectomised, must agree to use barrier contraception (condom plus spermicide) during heterosexual intercourse from screening through to study completion and for 99 days from the last dose of study investigational medicinal product

Exclusion criteria

Subjects who meet *any* of the following exclusion criteria are excluded from participating in the BUTEO trial.

1. Presence of documented secondary sclerosing cholangitis on prior clinical investigations.
2. Presence of alternative causes of liver disease, that are considered by the Investigator to be the predominant active liver injury at the time of screening, including viral hepatitis, alcoholic liver disease, non-alcoholic steatohepatitis, primary biliary cirrhosis. Patients with possible overlap syndrome with autoimmune hepatitis are excluded if the Investigator considers autoimmune hepatitis as the predominant liver injury.
3. AST and ALT >10 x ULN or bilirubin >3 x ULN or INR >1.3 in the absence of anti-coagulants
4. Serum creatinine >130µmol/L or platelet count <50 x 10^9/L
5. Any evidence of hepatic decompensation past or present, including ascites, episodes of hepatic encephalopathy or variceal bleeding
6. Recent cholangitis within last 90 days or ongoing need for prophylactic antibiotics
7. Pregnancy or breast feeding
8. Harmful alcohol consumption as evaluated by the Investigator
9. Flare in colitis activity within last 90 days requiring intensification of therapy beyond baseline maintenance treatment; use of oral prednisolone >10mg/day, biologics (i.e. monoclonal antibodies) and or hospitalisation for colitis within 90 days. Prior use of biologics is not a contraindication to screening
10. Diagnosed cholangiocarcinoma or high clinical suspicion of cholangiocarcinoma either clinically or by imaging
11. Concurrent malignancies or invasive cancers diagnosed within past 3 years except for adequately treated basal cell and squamous cell carcinoma of the skin and in situ carcinoma of the uterine cervix
12. Presence of a percutaneous drain or bile duct stent
13. Major surgical procedure within 30 days of screening
14. Prior organ transplantation
15. Known hypersensitivity to the investigational product or any of its formulation excipients
16. Unavailable for follow-up assessment or concern for subject's compliance
17. Participation in an investigational trial of a drug or device within 60 days of screening or 5 half-lives of the last dose of investigational drug, where the study drug half-life is greater than 12 days
18. Any other condition that in the opinion of the Investigator renders the subject a poor risk for inclusion into the study

For peer review only - http://bmjopen.bmj.com/site/about/guidelines.xhtml

19. Positive screening test for tuberculosis (TB) (including T-SPOT.TB TB test), unless respiratory review confirms false positive test results
20. Receipt of live vaccination within 6 weeks prior to baseline visit
21. Known HIV positive status

Trial Duration

It is anticipated that recruitment will take 2 years. The treatment period is 78 days and all patients will be followed up until Day 120, i.e. 42 days after the last administration of study treatment.

Trials Office Contact Details

BUTEO Trial Office
Cancer Research UK Clinical Trials Unit (CRCTU)
University of Birmingham
Birmingham
B15 2TT

Tel: 0121 414 4032 or 0121 414 8284

Fax 0121 414 8286

Email: BUTEO@trials.bham.ac.uk

For peer review only

Trial Schema

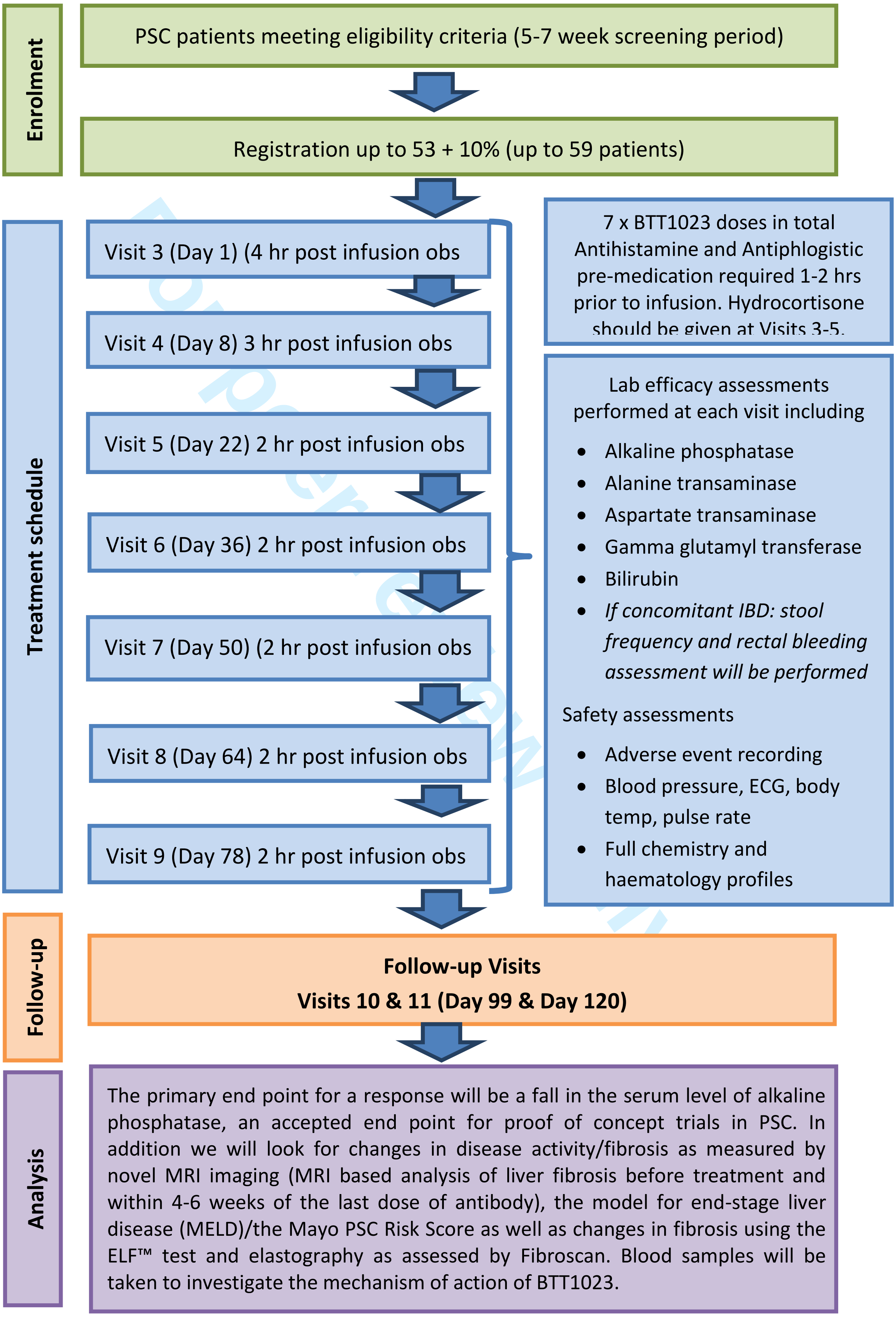

Schedule of Events

	Screening		Treatment							Follow-up	
	Screening Visit 1 within 5-7 weeks of Visit 3)	Screening Visit 2 (within 1 week of Visit 3)	Visit 3 (Infusion visit Day 1)	Visit 4 (Infusion visit Day 8) +/- 3 days	Visit 5 (Infusion visit Day 22) +/- 3 days	Visit 6 (Infusion visit Day 36) +/- 3 days	Visit 7 (Infusion visit Day 50) +/- 3 days	Visit 8 (Infusion visit Day 64) +/- 3 days	Visit 9 (Infusion visit Day 78) +/- 3 days	Visit 10 (Day 99) +/- 3 days	Visit 11 (Day 120) +/- 3 days
Informed consent	X										
Eligibility assessment	X	X									
Patient history	X	X									
Physical exam	X		X		X		X		X	X	X
Vital signs [1]	X		X	X	X	X	X	X	X	X	X
Vital signs during and post infusion [2]			X	X	X	X	X	X	X		
Electrocardiogram [3]	X		X	X	X	X	X	X	X	X	
BTT1023 infusion			X	X	X	X	X	X	X		
Standard blood tests[4]	X	X	X	X	X	X	X	X	X	X	X

For peer review only - http://bmjopen.bmj.com/site/about/guidelines.xhtml

	Screening		Treatment							Follow-up	
	Screening Visit 1 (within 5-7 weeks of Visit 3)	Screening Visit 2 (within 1 week of Visit 3)	Visit 3 (Infusion visit Day 1)	Visit 4 (Infusion visit Day 8) +/- 3 days	Visit 5 (Infusion visit Day 22) +/- 3 days	Visit 6 (Infusion visit Day 36) +/- 3 days	Visit 7 (Infusion visit Day 50) +/- 3 days	Visit 8 (Infusion visit Day 64) +/- 3 days	Visit 9 (Infusion visit Day 78) +/- 3 days	Visit 10 (Day 99) +/- 3 days	Visit 11 (Day 120) +/- 3 days
ALP sample for central analysis (pre infusion)			**X**	**X**	**X**	**X**	**X**	**X**	**X**	**X**	**X**
Standard blood tests 2hours post infusion			**X**	**X**	**X**	**X**	**X**	**X**	**X**		
Blood + serum sample for biomarker and research blood analysis (inc ELF)	**X**	**X**	**X**	**X**	**X**	**X**	**X**	**X**	**X**	**X**	**X**
Blood serum for PK[6]			**X**	**X**	**X**	**X**	**X**	**X**	**X**	**X**	**X**
Blood serum for Anti-Drug Antibodies (ADA)			**X**[8]				**X**[8]		**X**[8]	**X**	**X**
Blood serum for PD (sVAP-1)			**X**[9]	**X**[8]	**X**[8]	**X**[8]	**X**[8]	**X**[8]	**X**[10]	**X**	**X**
MRI scan [7]	**X**										**X**

For peer review only - http://bmjopen.bmj.com/site/about/guidelines.xhtml

	**Screening**		**Treatment**							**Follow-up**	
	Screening Visit 1 (within 5-7 weeks of Visit 3)	Screening Visit 2 (within 1 week of Visit 3)	Visit 3 (Infusion visit Day 1)	Visit 4 (Infusion visit Day 8) +/- 3 days	Visit 5 (Infusion visit Day 22) +/- 3 days	Visit 6 (Infusion visit Day 36) +/- 3 days	Visit 7 (Infusion visit Day 50) +/- 3 days	Visit 8 (Infusion visit Day 64) +/- 3 days	Visit 9 (Infusion visit Day 78) +/- 3 days	Visit 10 (Day 99) +/- 3 days	Visit 11 (Day 120) +/- 3 days
Pregnancy test (female patients of childbearing potential only)	**X**	**X**			**X**		**X**		**X**		**X**
Mayo PSC Risk Score		**X**								**X**	
MELD score		**X**								**X**	
Fibroscan®		**X**								**X**	
T-SPOT.TB test	**X**										
Quality of Life questionnaires[11]		**X (IBD diary only if applicable)**	**X**			**X**				**X**	
Adverse/ Clinical events	**X**	**X**	**X**	**X**	**X**	**X**	**X**	**X**	**X**	**X**	**X**
Concomitant medications	**X**	**X**	**X**	**X**	**X**	**X**	**X**	**X**	**X**	**X**	**X**

1 Vital signs: heart rate, blood pressure, temperature and respiratory rate to be performed pre-infusion Visits 3-11

For peer review only - http://bmjopen.bmj.com/site/about/guidelines.xhtml

2 Vital signs: heart rate, blood pressure, temperature and respiratory rate to be performed:

- **Day 1:** 30 and 60 minutes after the start of the infusion, 30, 60, 90, 120, 180 and 240 minutes post end of drug infusion.
- **Day 8:** 30 and 60 minutes after the start of the infusion, 30, 60, 90, 120 and 180 minutes post end of drug infusion.
- **Day 22, 36, 50, 64 and 78:** 30 and 60 minutes after the start of the infusion, 30, 90, and 120 minutes post end of drug infusion.

3 12 lead electrocardiogram (ECG) to be performed pre-infusion and 60 minutes after the start of infusion.

4 Full blood count (FBC), urea, electrolytes, liver function tests (LFTs), eGFR (calculated by Cockcroft-Gault equation or alternative as per institutional practice – Appendix 1) international normalised ratio (INR) to be performed pre-infusion **Day 1, 8, 22, 36, 50, 64, 78, 99 and 120**.

5 FBC, urea, electrolytes, LFTs, INR to be performed 120 minutes after the start of infusion.

6 Blood serum for pharmacokinetics. See Tables 6-8 for timings.

7 MRI to be performed during screening period and 4-6weeks of end of treatment (last dose of antibody). A Liver multiscan should be performed where possible (or equivalent methodology, at sites where this is possible). Where this is not possible please perform an MRI with MRCP.

8 Blood serum samples: Pre-infusion.

9 Blood serum samples: Pre-infusion, 180 and 360 minutes post infusion start and 24 hours post end of drug infusion.

10 Blood serum samples: Pre-infusion, 120 minutes post infusion start and 24 hours post end of drug infusion.

11 Quality of Life Questionnaires to be completed pre-infusion on Visit 3 and Visit 6. If patient has IBD, please give the IBD diary to patient at Screening Visit 2, to be completed prior to Visit 3.

For peer review only

Abbreviations

ABPI	Association of the British Pharmaceutical Industry
ADA	Anti-Drug Antibodies
AE	Adverse Event
ALP	Alkaline Phosphatase
ALT	Alanine Transaminase
APTT	Activated Partial Thromboplastin Time
AST	Aspartate Transaminase
CRCTU	Cancer Research UK Clinical Trials Unit
CRF	Case Report Form
CTCAE	Common Terminology Criteria for Adverse Events
DMC	Data Monitoring Committee
DoH	Department of Health
ECG	Electrocardiogram
eGFR	Estimated Glomerular Filtration Rate
ELF	Enhanced Liver Fibrosis
eRDC	Electronic Remote Data Capture
ESLD	End-stage Liver Disease
FBC	Full Blood Count
GCP	Good Clinical Practice
GGT	Gamma Glutamyl Transferase
HRS	Hours
IBD	Inflammatory Bowel Disease
IFN	Interferon

For peer review only

IgG	Immunoglobulin G
IL	Interleukin
INR	International Normalised Ratio
IMP	Investigational Medicinal Product
ISF	Investigator Site File
LFT	Liver Function Test
LPLV	Last Patient Last Visit
MELD	Model for End-stage Liver Disease
MHRA	Medicines and Healthcare Products Regulatory Agency
MINS	Minutes
MRI	Magnetic Resonance Imaging
NaCl	Sodium Chloride
NCI	National Cancer Institute
NIHR	National Institute of Health Research
NSAID	Non-steroidal Anti-Inflammatory Drug
OBS	Observations
PD	Pharmacodynamics
PK	Pharmacokinetics
PSC	Primary Sclerosing Cholangitis
QoL	Quality of Life
RA	Rheumatoid Arthritis
REC	Research Ethics Committee
SAE	Serious Adverse Event
SAR	Serious Adverse Reaction

For peer review only

SSAO	Semicarbazide-Sensitive Amine Oxidase
SUSAR	Suspected Unexpected Serious Adverse Reaction
sVAP-1	Serum Vascular Adhesion Protein-1
TMG	Trial Management Group
TNF	Tumour Necrosis Factor
UDCA	Ursodeoxycholic Acid
UE	Urea and Electrolytes
VAP-1	Vascular Adhesion Protein-1
ULN	Upper Limit of Normal

For peer review only

Table of contents

1. Background and Rationale 1
1.1 Background 1
1.1.1 Existing Research 1
1.1.2 Pharmacology and Toxicology of BTT1023 2
1.2 Trial Rationale 3
1.2.1 Justification for Patient Population 3
1.2.2 Justification for Design and Dose Levels 3
1.2.3 Choice of Treatment 3
2. Aims, Objectives and Outcome Measures 4
2.1 Aims and Objectives 4
2.1.1 Primary Objectives 5
2.1.2 Secondary Objectives 5
2.2 Outcome Measures 5
3. Trial Design 5
3.1 Dose Limiting Toxicity 6
3.2 Dose Limiting Reporting Procedure 8
3.3 Dose Escalation Range 8
4. Eligibility 11
4.1 Inclusion Criteria 11
4.2 Exclusion Criteria 12
5. Consent and Screening 13
5.1 Informed Consent 13
5.2 Screening 13
6. Trial Entry 14
6.1 Pre-registration 14
6.2 Patient Registration 15
6.2.1 Patient Registration During Dose Confirmation Phase 15
6.2.2 Patient Registration During Dose Expansion Phase (Via Electronic Remote Data Capture System) 15
6.3 Emergency Registration 16
7. Treatment Details 16
7.1 Investigational Medicinal Product Preparation 16
7.1.1 Dosage Form and Composition 16
7.1.2 Instructions for Storage 17
7.1.3 Instructions for Handling the Investigational Medicinal Product 17
7.2 Trial Treatment 17
7.3 Treatment Schedule (Visit 3 – Visit 9) 18
7.4 Trial Schema 22
7.5 Assessments 23
7.5.1 Blood Chemistry and Haematology 23

For peer review only

7.5.2 Pharmacokinetics Scheduling ... 24
7.5.3 Physical Examination/Symptom Assessment ... 25
7.5.4 Mayo Risk Assessment ... 25
7.6 Sample Collection ... 26
7.6.1 Blood Samples ... 26
7.6.2 Pharmacokinetic, Anti-Drug Antibodies and Pharmacodynamic Samples ... 26
7.7 Dose Modifications ... 26
7.8 Treatment Compliance ... 26
7.9 Pre-Medication ... 26
7.10 Concomitant Medication ... 26
7.11 Contraception and Pregnancy ... 27
7.12 Patient Follow Up ... 27
7.13 Patient Withdrawal ... 28
8. Adverse Event Reporting ... 29
8.1 Reporting Requirements ... 29
8.1.1 Adverse Events ... 29
8.1.2 Hypersensitivity, Infusion Reactions and Infusion Related Reactions ... 30
8.1.3 Serious Adverse Events ... 30
8.1.4 Reporting Period ... 30
8.2 Reporting Procedure ... 31
8.2.1 Site ... 31
8.2.2 Trials Office ... 32
8.2.3 Reporting to the Competent Authority and Research Ethics Committee ... 32
8.2.4 Investigators ... 33
8.2.5 Data Monitoring Committee ... 33
8.2.6 Provider of Investigational Medicinal Product ... 33
8.2.7 Reporting to the Research Ethics Committee ... 33
9. Data Handling and Record Keeping ... 33
9.1 Data Collection ... 33
9.2 Archiving ... 36
10. Quality Management ... 36
10.1 Site Set-up and Initiation ... 36
10.2 On-site Monitoring ... 36
10.3 Central Monitoring ... 36
10.4 Audit and Inspection ... 37
10.5 Notification of Serious Breaches ... 37
11. End of Trial Definition ... 37
12. Statistical Considerations ... 37
12.1 Definition of Outcome Measures ... 38
12.1.1 Primary Outcome Measure ... 38

For peer review only

12.1.2 Secondary Outcome Measures ... 38
12.2 Analysis of Outcome Measures ... 38
12.3 Planned Sub Group Analyses ... 39
12.4 Planned Interim Analysis ... 39
12.5 Planned Final Analyses ... 39
12.6 Power Calculations ... 39
13.Trial Organisational Structure ... 39
13.1 Sponsor ... 39
13.2 Coordinating Centre ... 39
13.3 Trial Management Group ... 39
13.4 Trial Steering Committee ... 40
13.5 Data Monitoring Committee ... 40
13.6 Finance ... 40
14.Ethical Considerations ... 41
15.Confidentiality and Data Protection ... 41
16.Insurance and Indemnity ... 42
17.Publication Policy ... 42
18.Reference List ... 43
Appendix 1 – Equation For Estimated Glomerular Filtration Rate ... 44
Appendix 2 - Common Toxicity Criteria Gradings ... 45
Appendix 3 – T-SPOT.TB ... 46
Appendix 4 - Definition of Adverse Events ... 48
Appendix 5 - WMA Declaration of Helsinki ... 50

LIST OF FIGURES

Figure 1: Flow diagram showing the trial decision guidelines for the run-in period of the trial design incorporating a conventional 3+3 cohort design if dose escalation is required. ... 10
Figure 2: Study/Patient Flow Diagram ... 22

LIST OF TABLES

Table 1: Suspected DLT Procedure ... 7
Table 2: Dose Level ... 9
Table 3: BTT1023 Drug Infusion Table ... 18
Table 4: Blood Tests ... 23
Table 5: Immunology Blood Tests ... 24
Table 6: Visit 3+9 Pharmacokinetics Scheduling ... 24
Table 7: Visit 4 Pharmacokinetics Scheduling ... 25
Table 8: Visit 5-8 Pharmacokinetic Scheduling ... 25
Table 9: Description of Case Report Forms ... 33
Table 10: Possible Stop/Go Guidelines and Associated Error Rates ... 38

For peer review only

1. BACKGROUND AND RATIONALE

1.1 Background

End-stage liver disease, regardless of aetiology, is characterised by hepatic fibrosis culminating in liver cirrhosis and accompanying increasing risks of liver cancer, liver failure, portal hypertension and death. Preventing progressive liver fibrosis represents an important area of interest in the development of new drugs suitable for all patients with liver disease. Primary sclerosing cholangitis (PSC) is a prime example of a progressive inflammatory liver disease which is characterised by relentless liver fibrosis and a high unmet need for new therapies. PSC has a population incidence of 1.3/100,000 annually, with a prevalence of 16.2 per 100,000. It affects men and women with a median age of 41 and in 80% of cases is associated with Inflammatory Bowel Disease (IBD). More than 50% of patients require liver transplantation within 10-15 years of symptomatic presentation, reflecting the failure of medical therapies to make any impact on the clinical outcome. The progression of PSC to scarring, cirrhosis and hepatobiliary cancer is driven by a chronic inflammatory response and immune cell mediated destruction of bile ducts (Hirschfield, Karlsen *et al*. 2013). Our research implicates vascular adhesion protein-1 (VAP-1) in the inflammation that drives fibrogenesis in liver disease (Lalor, Edwards *et al.* 2002) and we have shown levels of sVAP-1 in serum are elevated in chronic liver diseases and correlate with histological fibrosis (Kurkijärvi, Adams *et al*. 1998, Kurkijärvi, Yegutkin *et al.* 2000). These observations underpin our proposal that VAP-1 has an important role in the progression of liver fibrosis. We now plan to test the hypothesis that inhibiting VAP-1 with a neutralising antibody (BTT1023) will reverse or delay fibrogenesis in patients with, PSC and to investigate the potential of VAP-1 as a biomarker in liver disease.

1.1.1 Existing Research

The homodimeric transmembrane sialoglycoprotein,VAP-1,is expressed in the vasculature where it functions as an adhesion molecule that mediates the transmigration of leukocytes from blood through the endothelial lining into surrounding tissues (Jalkanen and Salmi 2001). It possesses amine oxidase activity, known as semicarbazide-sensitive amine oxidase (SSAO) and in its soluble form accounts for most amine oxidase activity in human serum (Kurkijärvi, Yegutkin *et al.* 2000). Our experimental data show that VAP-1 acts as an adhesion receptor to support leucocyte recruitment in liver inflammation. In animal models this function is critical during fibrogenesis, when it is secreted by stellate cells in response to inflammatory stimuli. It helps position inflammatory cells and activated myofibroblasts in areas of ongoing fibrogenesis (unpublished). Thus, inhibition of VAP-1 is expected to impact both inflammation and fibrosis: of note treatment with an antibody against VAP-1 prevents fibrosis in murine models of liver injury. Based on the strong up-regulation of hepatic VAP-1 in PSC patients, we hypothesise that levels of sVAP-1 will correlate with the severity of fibrosis in PSC and predict patients at risk of progressive disease. We propose that BTT1023 will work in PSC by inhibiting leucocyte recruitment into the inflamed liver. Additionally, our findings that hepatic expression of VAP-1 and its release from the liver into blood are markedly increased in chronic inflammatory liver disease suggest it may act as an organ-specific marker of liver inflammation. Our unpublished data show particularly high levels of VAP-1 in patients with PSC, with another study (Weston, Shepherd *et al.* 2015) showing a strong correlation between sVAP-1/SSAO activity in serum and histological fibrosis scores in patients with fatty liver disease. These findings suggest that circulating sVAP-1 may correlate with hepatic fibrosis stage. Our data further suggests stellate cells

For peer review only - http://bmjopen.bmj.com/site/about/guidelines.xhtml

secrete sVAP-1 in response to inflammatory stimuli (unpublished). Based on the strong up-regulation of hepatic VAP-1 we have reported in PSC patients (Liaskou, Karikoski *et al.* 2011), we hypothesise that levels of sVAP-1/SSAO will correlate with the severity of fibrosis in PSC and predict patients at risk of progressive disease.

1.1.2 Pharmacology and Toxicology of BTT1023

BTT1023 is a fully human, monoclonal, anti-VAP-1 antibody. It blocks the adhesion function of VAP-1 thereby diminishing leukocyte entry into sites of tissue inflammation, and is thought to alleviate inflammatory and fibrotic conditions. BTT1023 is a monoclonal IgG4 antibody that has been produced in a mammalian cell line and is delivered as concentrate for solution for infusion at a strength of 20 mg/ml. BTT1023 binds human recombinant and soluble VAP-1 with equilibrium dissociation constants of 130-210 pM. *In vitro,* BTT1023 effectively reduces the chemo attractant-evoked migration of lymphocytes through an endothelial cell monolayer. BTT1023 does not affect the semicarbazide-sensitive amine oxidase activity of VAP-1. *In vivo*, blocking VAP-1 function with an anti-mouse VAP-1 antibody significantly alleviates inflammation in mouse models of arthritis and lung inflammation. Treatment with an antibody against VAP-1 also prevents hepatic fibrosis in murine models of liver injury. Based on a tissue cross-reactivity study, BTT1023 recognises human and cynomolgus monkey tissues similarly, with VAP-1 expression detected mainly in vascular endothelia, liver sinusoidal endothelium, smooth muscle, adipose tissue and alveolar wall. Deducing the elimination rate constant for BTT1023 has not so far been possible in the primate studies. Based on the accumulation upon repeated dosing, the elimination half-life appears in the range of 10-20 days in the cynomolgus monkey. Intravenous administration of BTT1023 weekly for four weeks at 10, 30 or 100 mg/kg and biweekly for 14 weeks at 4, 20 or 100 mg/kg was well tolerated in cynomolgus monkeys. No toxicologically relevant effects were observed in in-life recordings or laboratory tests, or in macroscopic or histopathology. Toxicokinetic monitoring showed an adequate exposure throughout the studies. The estimated no observed adverse effect level was 100 mg/kg when administered biweekly for 14 weeks (Biotie Therapies Corp., 2008).

No adverse safety pharmacological effects were detected in conjunction with the toxicity studies when cardiovascular and ophthalmic signs were investigated. BTT1023 was free of irritant effects in both repeat dose toxicity studies. Secondary antibodies were not detected in the 4-week toxicity study. In the 14-week study an immune response against BTT1023 was detected in 7/10 animals in the low 4 mg/kg dose group on day 85. BTT1023 does not stimulate cytokine release in the cynomolgus monkey *in vivo* or from human whole blood *in vitro*. As an IgG molecule, BTT1023 is not anticipated to interfere with chromosomal material. Teratogenicity has not been studied. In the first-in-man trial with BTT1023, infusion-related adverse events (AEs) were seen in two out of five healthy volunteers in the highest dose (2 mg/kg) cohort and included flushing, tingling, numbness and oedema. No cytokine release syndrome was recorded. In subsequent trials up to 8 mg/kg of BTT1023 was dosed to patients with rheumatoid arthritis or psoriasis after a premedication (cetirizine 10 mg, ibuprofen 600 mg orally). The frequency and the intensity of the AEs observed were not increased with dose increments and BTT1023 appeared safe and well-tolerated in repeated intravenous dosing. In a three-month trial in patients with rheumatoid arthritis, 6/10 patients treated with 4 or 8 mg/kg BTT1023 experienced a 50% or greater reduction in disease activity according to American College for Rheumatology response criteria during the treatment period and 4/10 had maintained this

For peer review only - http://bmjopen.bmj.com/site/about/guidelines.xhtml

response when assessed a month after the last dose. No patient on placebo met these criteria Biotie Therapies Corp., 2010).

Similarly to other therapeutic proteins, BTT1023 may be immunogenic, but this has not been confirmed to date. No specific antidote to BTT1023 is available.

1.2 Trial Rationale

1.2.1 Justification for Patient Population

PSC is a progressive immune mediated biliary disease characterised by bile duct inflammation and fibrosis and accompanying hepatic fibrosis. For patients with elevated alkaline phosphatase (ALP) in particular, progressive disease is predicted, which currently results in a need for liver transplantation in the majority of cases. No current medical therapy has as yet been shown to be effective in altering the natural process of the disease. For this reason patients with PSC who have elevated ALP values will be recruited to this trial, in order to evaluate the impact of VAP-1 blockade by BTT1023, in an early phase trial focused on biochemical efficacy and safety.

1.2.2 Justification for Design and Dose Levels

This is an early phase trial of BTT1023 in immune mediated liver disease, with the rationale to identify biochemical efficacy of effect (reduction in ALP) and safety, in an orphan disease for PSC that presently lacks any other medical therapy. The trial design therefore focuses on identifying early biochemical efficacy signals to justify larger scale, randomised controlled trials of a longer duration.

The selection of the proposed dose and dosing interval for the trial is based on information obtained from previous multiple-dose clinical trials with BTT1023. The proposed dose level of 8 mg/kg was well tolerated by RA and psoriasis patients in previous clinical trials (BTT12-CD015and BTT2-CD016 respectively), and is therefore considered to provide appropriate bridging into existing clinical data.

Due to its mechanism of action, it is likely that a therapeutic response with BTT1023 will be best achieved through continuous exposure at therapeutic plasma levels. Consequently, the preferred dosing regimen should ensure saturation of the endogenous VAP-1 antigen pool as rapidly as possible. In the RA trial, patients received five 8 mg/kg doses of BTT1023 administered at biweekly intervals. With this dosing regimen, potentially efficacious trough concentrations of BTT1023 were observed by day 42 (i.e. before the 4th infusion). In an attempt to accelerate saturation of the endogenous VAP-1 antigen pool, a more rapid loading scheme was utilised in the psoriasis trial, in which patients received three doses of BTT1023 on days 1, 8 and 22. Due to the limited amount of pharmacokinetic data, a detailed comparison of exposures reached with the two dosing regimens is not feasible. Most importantly however, both administration schemes were well tolerated, with no infusion-related adverse events reported in either trial. Therefore, it is proposed that the accelerated dosing scheme (i.e. dosing on days 1, 8, and biweekly thereafter) be deployed in the current study.

1.2.3 Choice of Treatment

A key factor in establishing and maintaining chronic inflammation is the excessive accumulation of leukocytes in tissue after they exit the blood and move towards the site of inflammation through a series of processes which include attachment to specific adhesion proteins. VAP-1 is one such adhesion molecule up-regulated principally at the site of inflammation, limiting potential side effects of a product targeting this pathway. Our research in particular implicates VAP-1 in progressive

For peer review only - http://bmjopen.bmj.com/site/about/guidelines.xhtml

fibrosis in liver disease and we would now like to test the hypothesis that inhibiting VAP-1 with a neutralising antibody (BTT1023) will reverse or delay fibrogenesis in patients with PSC. A single arm, rather than placebo controlled design, was chosen to allow efficient enrolment of patients into the study due to its intensive nature in which a significant chance of being allocated into the placebo group may act as a substantial barrier for enrolment. The proposed primary endpoint (ALP) is a biochemical measurement and thus not subject to subjective bias as a clinical assessment would be, rendering the necessity of a concurrent control group less vital. Our data on stability of ALP in PSC suggests that responses, as defined in the protocol, occur very seldom during the natural course of the disease, and we can therefore reliably assess changes from baseline and response rates for this proof of concept study to evaluate the therapeutic potential of the investigational product. In addition we will measure serial ALP levels before starting treatment, at day 1, and exclude patients in whom the levels change significantly (>25%). ALP levels will also be measured at Visit 10 (Day 99) which is 21 days after the last treatment on Visit 9 (Day 78). Toxicology data with BTT1023 is available for up to 14 weeks, limiting dosing of BTT1023 to patients to three months.

We also want to investigate the potential of soluble VAP-1 as a biomarker of liver disease. The rationale for this study is based on our findings that VAP-1 release from the liver into blood is markedly increased in chronic inflammatory liver disease suggesting it may act as an organ-specific marker of liver inflammation (Kurkijärvi, Yegutkin *et al.* 2000). Our recent data shows particularly high levels in patients with PSC. In another study submitted for publication we found a strong correlation between sVAP-1/SSAO activity in serum and histological fibrosis scores in patients with fatty liver disease. This suggests that circulating sVAP-1/SSAO may correlate with hepatic fibrosis stage. Based on the strong up-regulation of hepatic VAP-1 we have previously reported in PSC patients, we hypothesise that levels of sVAP-1/SSAO will correlate with the severity of fibrosis in PSC and predict patients at risk of progressive disease. Reliable biomarkers that correlate with fibrosis stage and progression of liver disease are in demand in order to predict outcome and to stage disease without the need for invasive liver biopsy. This research will allow us to translate laboratory research into a proof of activity clinical trial incorporating experimental medicine studies that will elucidate the role of VAP-1 in liver fibrosis and its potential as a therapeutic target and biomarker.

For peer review only

2. AIMS, OBJECTIVES AND OUTCOME MEASURES

2.1 Aims and Objectives

The overall aims of the study are:

1) To determine the short term activity and safety of an anti-VAP-1 antibody BTT1023 in patients with PSC
2) To confirm the safe and effective dose of BTT1023 in patients with PSC
3) To provide insights into the mechanisms of action of VAP-1/SSAO
4) To create the framework for subsequent larger scale interventions in chronic liver disease with anti-VAP-1 antibody
5) To develop soluble VAP-1/SSAO as a biomarker for liver disease by correlating serum VAP-1 (sVAP-1/SSAO) levels to liver fibrosis, severity of inflammation and clinical outcome

For peer review only - http://bmjopen.bmj.com/site/about/guidelines.xhtml

2.1.1 Primary Objectives

1) To determine the activity of the anti-VAP-1 antibody BTT1023 in patients with PSC as measured by a decrease in ALP levels (primary endpoint) with secondary endpoints to include various measures of liver injury and fibrosis
2) To evaluate the safety, effective dose and tolerability of BTT1023 in patients with PSC

2.1.2 Secondary Objectives

1) To determine the mechanisms of action of BTT1023 through in vivo assessment of VAP-1/ (SSAO) enzyme activity and immune cell function
2) To evaluate the potential of a novel MRI based assessment of liver fibrosis and biliary strictures for assessing therapeutic response in PSC
3) Assess the use of sVAP-1/SSAO as a biomarker to monitor disease progression in PSC

2.2 Outcome Measures

Primary:

1) Response at Visit 10 (Day 99): a reduction in serum ALP by 25% or more from baseline to Visit 10 (Day 99)

Secondary:

1) Safety and tolerability:
   - Treatment Compliance (including patient withdrawal) and Serious Adverse Event (SAE) and Adverse Event (AE) frequency
2) An improvement from baseline to Visit 10 (Day 99) in:
   - Quality of life questionnaires: EQ-5D, Fatigue Severity Scale, Pruritus Visual Analogue Score (VAS), IBD Diaries (if applicable)
   - Tests of liver fibrosis: Enhanced Liver Fibrosis (ELF) and Fibroscan
   - Individual markers of liver biochemistry and function (AST, ALT, ALP, GGT, Bilirubin, Albumin, INR) and composite risk scores (Mayo PSC Risk score and MELD)
   - Change (improvement) in LiverMultiscan® MRI imaging (or equivalent methodology, at sites where this is possible) – Liver MRI is an emerging method for monitoring liver disease and its treatment. We will evaluate changes in MRI imaging pre and post therapy using the LiverMultiscan® protocol

3) Evaluate changes in sVAP-1/SSAO as a biomarker of liver disease activity across the study period

3. TRIAL DESIGN

This is a two-stage single-arm, open-label, multi-centre hybrid trial. Data from previous studies of RA and psoriasis patients supports the use of a therapeutic dose of 8 mg/kg which was well tolerated. The trial is comprised of two components – an initial run-in period to confirm the therapeutic dose, which leads into a Simon's two-stage design. The success criteria for transition from the dose confirmation period to the Simon's two-stage design have been approximated as reaching a trough concentration of 3 µg/ml free circulating BTT1023 at 8 weeks from first infusion, which is about 100 fold the dissociation constant (K_d) of BTT1023 from VAP-1 and should result in target occupancy of

For peer review only - http://bmjopen.bmj.com/site/about/guidelines.xhtml

approximately 90%, with either the starting dose of 8 mg/kg or following dose escalation up to a maximum of 16 mg/kg.

3.1 Dose Limiting Toxicity

A Dose Limiting Toxicity (DLT) is defined as an AE that meets the criteria of grade 4 or grade 5 as defined in the Common Terminology Criteria for Adverse Events (CTCAE V4.0) (Appendix 2). However, if a definite diagnosis of cytokine release syndrome is made, it will be classed as a DLT at grade 3 and above* and must be considered at least possibly related to BTT1023.
Although previous studies have shown no DLTs to be expected with BTT1023, toxicity monitoring will be on going throughout the trial.

During the confirmatory stage the DLT reporting period is defined as the treatment period from treatment Visit 3 up to Visit 10 (Day 99 follow up visit).

The decision process upon notification of a second suspected DLT will be made in accordance to Table 1 below.

**In order to confirm the case of cytokine release syndrome, an additional blood sample will need to be taken for cytokine assay (Tumour necrosis factor-alpha (TNF-α), Inteferon-gamma (IFN-γ), Interleukin-6 (IL-6). This information is to be provided as part of the SAE form.*

For peer review only

Table 1: Suspected DLT Procedure

Stage	Description	Action
1	Notification of second potential DLT received by study team	- All relevant medical data is received and reviewed by clinical coordinator according to standard SAE timelines (7 or 15 days)<br>NB: it is encouraged that the team will meet and report all DLTs to the Data Monitoring Committee (DMC) as and when they occur depend on the timing (interval between DLTs) it may be necessary to have more frequent DMC meetings or discuss both DLTs at the same time.
2	Formal confirmation of 2nd DLT by designated clinical coordinator (member of Trial Management Group [TMG])	- Recruitment is temporarily suspended due to safety concerns<br>- Sites immediately informed / local R&D offices informed<br>NB: any patients currently receiving therapy will continue to receive therapy at the local treating clinician's discretion, in consultation with the clinical coordinator. This decision is to be documented in the patient record.
3	DMC meeting called to review all safety information	- Arranged within 14-21 days ideally (arranged by TMG)<br>- Safety report provided to DMC by TMG<br>- DMC Chair- person to make formal recommendation to TSC/TMG
4	Trial Steering Committee (TSC) meeting to be arranged	- To review DMC recommendations (within 7-21 days of DMC meeting)
5	Inform Medicines and Healthcare products Regulatory Authority (MHRA) and Research Ethics Committee (REC) of decision if trial to close due to safety concerns is reached	- All sites will be informed immediately (within 24 hrs) by the TMG upon notification of the recommendation. Recruitment into the trial will stop with immediate effect.<br>- MHRA and REC will be informed of permanent closure of the study in accordance with UK regulations.<br>- Specific circumstances of patients still receiving therapy will be assessed on an individual basis with the clinical coordinator and the local study team. A decision will be made whether to continue therapy for the individual concerned and documented in the medical records.

For peer review only

3.2 Dose Limiting Reporting Procedure

On becoming aware that a patient has experienced a DLT, the Investigator (or delegate) must complete, date and sign an SAE Form (ensuring the DLT section is completed appropriately). The form should be faxed together with a SAE Fax Cover Sheet to the Trials Office using one of the numbers listed below as soon as possible and no later than 24 hrs after first becoming aware of the event:

To report a DLT, fax the completed SAE Form with an SAE Fax Cover Sheet to:

0121 414 8286 (primary number)

0121 414 3529 (secondary number)

NB: in addition to the above, sites are requested to directly contact the Trials Office, via phone (0121 414 4032 or 0121 414 8284) or e-mail (BUTEO@trials.bham.ac.uk) stating a suspected DLT has occurred.

3.3 Dose Escalation Range

Dose escalation of BTT1023 is dependent upon the levels of BTT1023 in circulating blood serum. Data previously obtained from studies in RA and psoriasis patients indicates that a therapeutic dose of 8 mg/kg should be effective. However, if successful pharmacokinetic criteria are not met following exposure at 8 mg/kg, the doses of BTT1023 will be escalated to 12 or 16 mg/kg as appropriate. In the event that the 16 mg/kg is tested and found not to meet the success criteria then the trial will stop. If, following a dose escalation, there is evidence of concerns regarding toxicity, an intermediate dose may be chosen for clinical evaluation. Any subsequent change to dose levels outside those specified in protocol (8, 12 and 16 mg/kg) will need approval by the DMC. A substantial amendment will also need to be prepared and approved by the national ethics and regulatory authorities before this intermediate dose can be clinically evaluated.

Run-In Dose Confirmatory Period

The run-in component of the trial has the potential to incorporate a conventional 3+3 cohort design with decision guidelines based on toxicity and pharmacokinetic (PK) data. The flow diagram in Figure 1, illustrates the trial's progression during the run-in period, specifying decision stages based on observed patient results. The trial begins with the recruitment of 6 patients all starting on the treatment dose of 8 mg/kg. During Visit 7 (Day 50), blood tests will be carried out to measure the serum BTT1023 to assess circulating PK levels at trough.

Recruitment will be paused after the sixth patient while awaiting PK results (this should take no longer than 7 weeks) and until the DLT reporting period is completed for each patient (up to Visit 10 [Day 99]). Toxicity monitoring will be on-going continually throughout the duration of the trial. An acceptable DLT rate has been established for the trial as a maximum of 1 incidence in 6 patients (~17%).

For peer review only - http://bmjopen.bmj.com/site/about/guidelines.xhtml

If the DLT rate rises to 2 or more, at any stage during the DLT reporting period, (Visit 3 [Day 1] to Visit 10 [Day 99]) the trial will be halted in consultation with the DMC (see Table 1).

The trial will continue into Phase II if results from the initial cohort of 6 patients, on the 8 mg/kg dose, show an acceptable DLT rate and trough levels of BTT1023 meet the stipulated success criteria of 3 µg/ml free circulating antibody. In the event that DLT rate is acceptable but the PK levels do not meet the success criteria then the trial moves into a conventional 3+3 design. In this event, the original 6 patient cohort will no longer be evaluated, but a new cohort of 3 patients will be recruited to receive the newly identified test dose. If there are no DLTs at Visit 10 [Day 99], an additional cohort of 3 patients will be recruited at the new test dose. During Visit 6 [Day 50], blood tests will be carried out to assess the level of serum BTT1023. If the circulating level of BTT1023 is found to be too low there are 2 potential dose escalation increments of 12 and 16 mg/kg (Table 2), and if it is found to be too high (such as resulting in trough levels consistently exceeding 100 µg/ml) then there is potential to de-escalate the dose in agreement with the DMC.

In the escalation phase, if a patient does not complete the Visit 7 (Day 50) visit, they will be replaced.

Once a confirmed dose has been established, the trial will continue into Phase II, continuing to include those patients who have previously been receiving the confirmed dose. Those patients not receiving the confirmed dose will not be included in the Phase II evaluation. If dose escalation occurs and the maximum dose of 16 mg/kg is utilised and found not to result in sufficient blood levels of BTT1023 then the trial will be stopped. The trial will be stopped at any stage where patient safety is compromised.

Table 2: Dose Level

Dose Level	Dose
1	8 mg/kg
2	12 mg/kg
3	16 mg/kg

Phase II – Single-arm Simon's 2-stage design

The trial design incorporates an interim analysis of the accumulating data. The interim analysis (stage-1) takes place once 18 patients have been evaluated for the primary outcome – which is based on the response level of ALP. If three or more responses are observed in stage-1 then the trial will continue into stage-2. Recruitment will not be halted while stage-1 is assessed. Further patients will be recruited during stage-2 in order to obtain the required sample size of 37 patients – allowing for 10% patient drop out during trial duration, this number could reach a total of 41 patients recruited.

If overall there are nine or more responses from 37 evaluable patients then we conclude that the treatment warrants further investigation. If the prescribed patient number is not met then the design parameters will be re-evaluated. Patients treated at the confirmed dose during the run-in period will contribute to the total patient requirement.

For peer review only - http://bmjopen.bmj.com/site/about/guidelines.xhtml

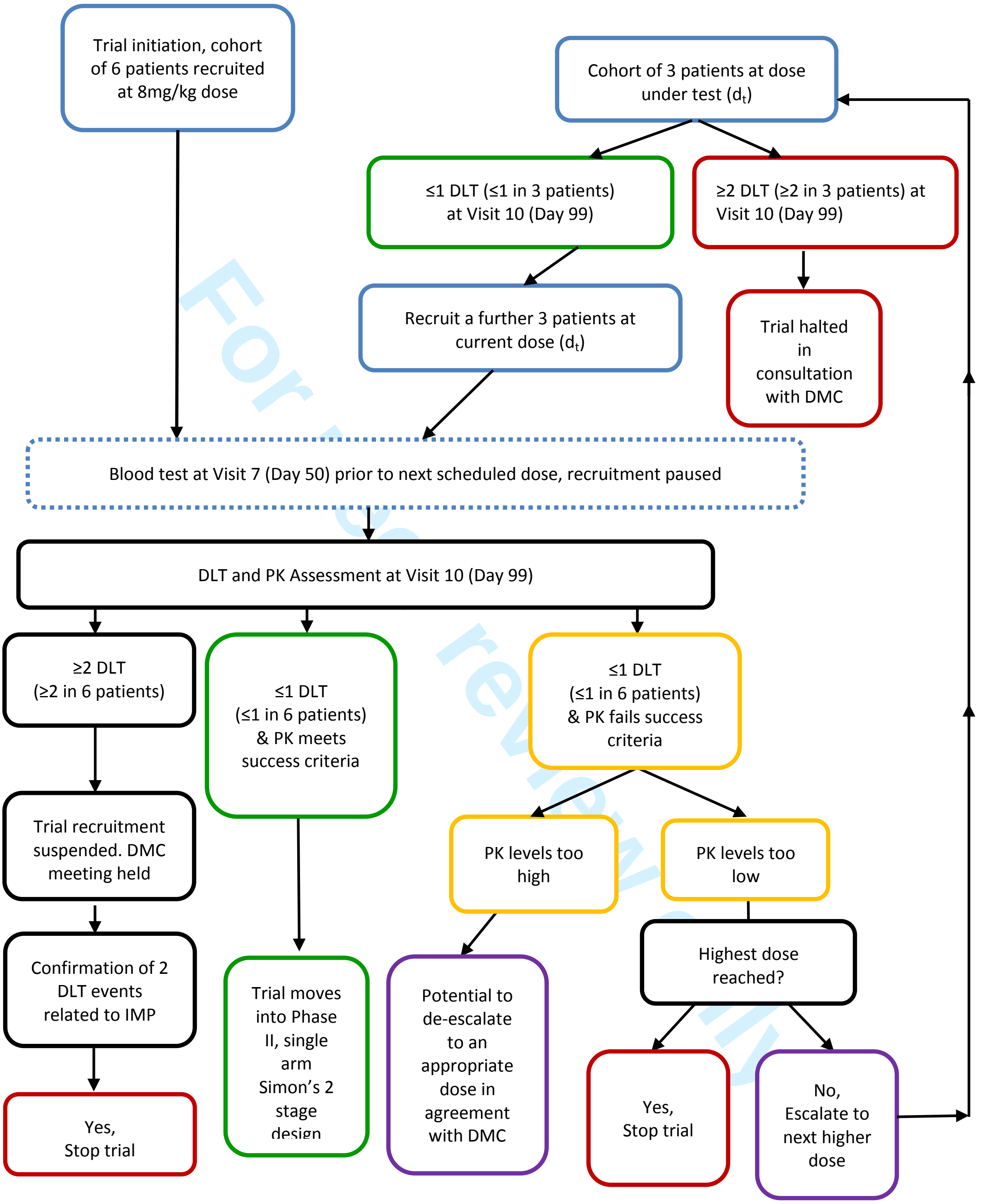

Figure 1: Flow diagram showing the trial decision guidelines for the run-in period of the trial design incorporating a conventional 3+3 cohort design if dose escalation is required.

To enable collection of long-term safety data, if in the opinion of the DMC a sufficiently positive risk-benefit profile has been observed (a clinically meaningful decrease in ALP for individual subjects in the absence of significant safety signals in the study population), subjects may (pending regulatory approval of an appropriate Substantial Amendment) participate in an open label extension treatment phase not to exceed 1 year in duration. During the open label extension phase, dose level, safety monitoring, safety reporting, and treatment stopping criteria will remain the same as during the initial 11-week treatment phase of the study.

4. ELIGIBILITY

4.1 Inclusion Criteria

Subjects must meet ***all*** of the following inclusion criteria to be eligible for participation in the clinical trial:

1. Males and females 18 - 75 years of age who are willing and able to provide informed, written consent and comply with all study requirements
2. Clinical diagnosis of PSC as evident by chronic cholestasis of more than six months duration with either a consistent MRI showing sclerosing cholangitis or a liver biopsy consistent with PSC in the absence of a documented alternative aetiology for sclerosing cholangitis
3. In those with concomitant Inflammatory Bowel Disease, clinical and colonoscopic evidence within the last year of stable disease, without findings of high grade dysplasia
4. In those on treatment with UDCA, therapy must be stable for at least 3 months, and at a dose not greater than 20mg/kg/day. In those not on treatment with UDCA at the time of screening, a minimum of 8 weeks since the last dose of UDCA should be recorded
5. Serum ALP greater than 1.5 x ULN
6. Stable serum ALP levels (levels must not change by more than 25% from Screening Visit 1 and Screening Visit 2)
7. Female subjects of childbearing potential must have a negative pregnancy test prior to starting study treatment. For the purposes of this study, a female subject of childbearing potential is a woman who has not had a hysterectomy, bilateral oophorectomy, or medically-documented ovarian failure. Women ≤ 50 years of age with amenorrhea of any duration will be considered to be of childbearing potential
8. All sexually active women of childbearing potential must agree to use two forms of highly effective method of contraception from the Screening Visit throughout the study period and for 99 days following the last dose of study drug. If using hormonal agents the same method must have been used for at least 1 month before study dosing and subjects must use a barrier method as the other form of contraception. Lactating women must agree to discontinue breast feeding before study investigational medicinal product administration
9. Men, if not vasectomised, must agree to use barrier contraception (condom plus spermicide) during heterosexual intercourse from screening through to study completion and for 99 days from the last dose of study investigational medicinal product

For peer review only - http://bmjopen.bmj.com/site/about/guidelines.xhtml

4.2 Exclusion Criteria

Subjects who meet *any* of the following exclusion criteria are excluded from participating in the BUTEO trial.

1. Presence of documented secondary sclerosing cholangitis on prior clinical investigations
2. Presence of alternative causes of liver disease, that are considered by the Investigator to be the predominant active liver injury at the time of screening, including viral hepatitis, alcoholic liver disease, non-alcoholic steatohepatitis, primary biliary cirrhosis. Patients with possible overlap syndrome with autoimmune hepatitis are excluded if the Investigator considers autoimmune hepatitis as the predominant liver injury
3. AST and ALT >10 x ULN or bilirubin >3 x ULN or INR >1.3 in the absence of anti-coagulants
4. Serum creatinine >130µmol/L or platelet count <50 x 10^9/L
5. Any evidence of hepatic decompensation past or present, including ascites, episodes of hepatic encephalopathy or variceal bleeding
6. Recent cholangitis within last 90 days or ongoing need for prophylactic antibiotics
7. Pregnancy or breast feeding
8. Harmful alcohol consumption as evaluated by the Investigator
9. Flare in colitis activity within last 90 days requiring intensification of therapy beyond baseline maintenance treatment; use of oral prednisolone >10mg/ day, biologics (i.e. monoclonal antibodies) and/or hospitalisation for colitis within 90 days. Prior use of biologics is not a contraindication to screening
10. Diagnosed cholangiocarcinoma or high clinical suspicion cholangiocarcinoma either clinically or by imaging
11. Concurrent malignancies or invasive cancers diagnosed within past 3 years except for adequately treated basal cell and squamous cell carcinoma of the skin and in situ carcinoma of the uterine cervix
12. Presence of a percutaneous drain or bile duct stent
13. Major surgical procedure within 30 days of screening
14. Prior organ transplantation
15. Known hypersensitivity to the investigation product or any of its formulation excipients
16. Unavailable for follow-up assessment or concern for subject's compliance
17. Participation in an investigational trial of a drug or device within 60 days of screening or 5 half-lives of the last dose of investigational drug, where the study drug half-life is greater than 12 days
18. Any other condition that in the opinion of the investigator renders the subject a poor risk for inclusion into the study
19. Positive screening test for tuberculosis (including T-SPOT.TB TB test), unless respiratory review confirms false positive test results

22. Receipt of live vaccination within 6 weeks prior to baseline visit

20. Known HIV positive status

For peer review only

5. CONSENT AND SCREENING

5.1 Informed Consent

It is the responsibility of the Principal Investigator (PI) or Co-investigators (detailed in the site delegation log) to obtain written informed consent for each patient prior to performing any trial related procedure. A Patient Information Sheet (PIS) is provided to facilitate this process. Investigators must ensure that they adequately explain the aim, trial treatment, anticipated benefits and potential hazards of taking part in the trial to the patient. The investigator should also stress that the patient is completely free to refuse to take part or withdraw from the trial at any time without any detrimental effect on their standard care. The patient should be given ample time (e.g. 24 hrs) to read the PIS and to discuss their participation with others outside of the site research team. The patient must be given an opportunity to ask questions which should be answered to their satisfaction. The right of the patient to refuse to participate in the trial without giving a reason must be respected.

If the patient expresses an interest in participating in the trial they should be asked to sign and date the latest version of the Informed Consent Form (ICF). The PI or Co-investigator (detailed in the site delegation log) must then sign and date the form. A copy of the ICF should be given to the patient, a copy should be filed in the hospital notes, and the original placed in the Investigator Site File (ISF). Once the patient is entered into the trial the patient's trial number should be entered on the ICF maintained in the ISF. In addition, if the patient has given explicit consent a copy of the signed Informed Consent Form must be sent in the post to the Trials Office for review.

Details of the informed consent discussions should be recorded in the patient's medical notes, this should include date of, and information regarding, the initial discussion, the date consent was given, with the name of the trial and the version number of the PIS and ICF. Throughout the trial the patient should have the opportunity to ask questions about the trial and any new information that may be relevant to the patient's continued participation should be shared with them in a timely manner. On occasion it may be necessary to re-consent the patient in which case the process above should be followed and the patient's right to withdraw from the trial respected.

Electronic copies of the PIS and ICF are available from the BUTEO Trials Office and should be printed or photocopied onto the headed paper of the local institution.

Details of all patients approached about the trial should be recorded on the Patient Screening/Enrolment Log and with the patient's prior consent their General Practitioner (GP) should also be informed that they are taking part in the trial. A GP Letter is provided electronically for this purpose.

5.2 Screening

Prior to formal screening process patients will be required to provide written informed consent **BEFORE** any non-standard of care assessment can be performed, as detailed in section 5.1. It is anticipated that the majority of the screening process (except MRI imaging) will be completed at the same visit (i.e. all blood tests performed on the same day).

Each patient who has consented, and enters the screening process, will be allocated a screening number. Each site will be provided with a Patient Screening Enrolment log, which is pre-populated with site specific screening numbers. Please use this number in any correspondence with the BUTEO

For peer review only - http://bmjopen.bmj.com/site/about/guidelines.xhtml

Trial Office. If the patient is eligible for the trial, the screening number should be completed on the Registration Form. A Trial Number will be allocated once the patient is registered.

The screening process consists of two visits which are detailed below:

Screening Visit 1 (within 5-7 weeks of Visit 3):

- Vital signs (heart rate, blood pressure, temperature, respiratory rate)
- Physical exam to be performed by clinician
- 12-lead electrocardiogram (ECG)
- Bloods for Full Blood Count (FBC), International Normalised Ratio (INR), Urea and Electrolytes (UEs), Liver Function Tests (LFTs)
- Blood sample (whole blood and serum) for biomarker and research blood analysis
- Medical history including any current symptoms any pre-existing conditions
- Record of patient concomitant medication (only record con-meds that are currently being taken by the patient)
- Evaluation of inclusion and exclusion criteria
- MRI scan (to be performed between consent and registration)
- TB screening - T-SPOT.TB test (see Appendix 3)
- Pregnancy test (for females patients of childbearing potential only)

Screening Visit 2 (within 1 week of Visit 3):

- Bloods for FBC, INR, UEs, LFTs
- Blood sample (whole blood and serum) for biomarker and research blood analysis
- Record of patient concomitant medication
- Patient medical history (including current symptom and any conditions since Visit 1)
- Pregnancy test (for females patients of childbearing potential only)
- Mayo PSC Risk Score
- MELD score
- Fibroscan
- Give IBD Diary (if applicable)
- Evaluation of the inclusion/exclusion criteria

If the repeat ALP measurement: is within +/- 25% of previous sample (Visit 1) and the patient continues to be eligible for the study, proceed to patient registration (see section 6 of the study protocol for procedure)

6. TRIAL ENTRY

6.1 Pre-registration

After the results of the screening process (Visits 1 and 2) are available, the following will be verified by the local site PI and/or Co-Investigators before registering the patient onto the trial:

- Patient's ICF
- Confirmation of all the inclusion criteria
- Review of all the exclusion criteria

For peer review only - http://bmjopen.bmj.com/site/about/guidelines.xhtml

- Patient's Screening Number
- Completion of Registration Form

All the above must be completed before proceeding onto patient registration.

6.2 Patient Registration

All patients must be registered with the CRCTU at the University of Birmingham and their Screening Number provided during the registration process.

6.2.1 Patient Registration During Dose Confirmation Phase

During the dose confirmation phase of the clinical trial all participating centres are required to contact the study office directly **before** approaching any potential patient. This is important to confirm whether a slot is available with the BUTEO Trial Office. In addition during this phase of the study the site office will be in contact on a weekly basis with sites, when any cohort is near to completion. This will ensure that patients are only approached by study sites, if there is a likelihood of them being recruited into the study. Site investigators are also required to keep the BUTEO office informed of any circumstances (patient refusal, screen failure, previously recruited patients fail to reach Visit 7 [Day 50] etc.) that may mean that previously allocated slots become available.

All patients that are eligible for inclusion in the trial and have signed an ICF will be registered via the BUTEO Trials Office. Informed consent must be obtained prior to registration. An Eligibility Checklist and Registration Form must be completed and sent to the BUTEO Trial Office.

The patient's eligibility will be confirmed at registration by the BUTEO Trial Office. If eligible for the study, the patient will be allocated a unique Trial Number. Study drug prescriptions must include the patient's Trial Number and dose. The registration line will be open during office hours (09:00 – 17:00, Monday-Friday).

Registration line: TEL: 0121 414 4032 or 0121 414 8284

FAX: 0121 414 8286

(09:00 – 17:00, Monday-Friday)

At this time point, patients will be allocated a unique trial number to preserve patient confidentiality. The schedule for investigations and follow up visits is summarised in section 7.3.

6.2.2 Patient Registration During Dose Expansion Phase (Via Electronic Remote Data Capture System)

All sites will be informed and provided with full instructions when the online electronic Remote Data Capture system (eRDC) has been implemented.

All patients that are eligible for inclusion in the trial and have signed an ICF will be registered via the online electronic remote data capture system (eRDC). Informed consent must be obtained prior to registration. An Eligibility Checklist must also be completed and faxed to the Trials Office before the online Registration Form is completed.

A copy of the patient's ICF must be sent in the post to the BUTEO Trial Office, if the patient has given explicit consent for this.

For peer review only - http://bmjopen.bmj.com/site/about/guidelines.xhtml

In the expansion phase of the trial, registration of patients can be achieved by logging on to:

https://www.cancertrials.bham.ac.uk

Full details will be provided to sites when this is in use.

At this time point, patients will be allocated a unique trial number to preserve patient confidentiality. The schedule for investigations and follow up visits is summarised in section 7.3.

NB. Once the eRDC system has been implemented, patient's will NOT be registered via the method in section 6.2.1.

6.3 Emergency Registration

Once the eRDC has been implemented, in case of any problems with online registration, a paper eligibility checklist and registration form should be completed. These details should then be phoned through to the Registration Office at CRCTU, University of Birmingham using the below numbers:

Contact details for emergency registration

Cancer Research UK Clinical Trials Unit (CRCTU)
Vincent Drive
University of Birmingham
B15 2TT
Tel: 0121 414 3366
(9am-5pm, excluding weekends)
Fax: 0121 414 7989

After an emergency registration, patients will be given a unique trial number which will be allocated in sequence by the CRCTU as detailed above.

7. TREATMENT DETAILS

7.1 Investigational Medicinal Product Preparation

The Investigational Medicinal Product (IMP) is BTT1023. This is a fully-human IgG4 immunoglobulin with antigen-binding specificity for human VAP-1. It consists of two κ light chains and two heavy chains with a total molecular mass of approximately 146 kDa. BTT1023 is produced in a Chinese hamster ovary cell culture and purified with appropriate methods including specific viral inactivation and removal procedures. Additional information is contained within the BTT1023 Investigator Brochure.

7.1.1 Dosage Form and Composition

The dosage form of BTT1023 is concentrate for solution for infusion (see latest version of Biotie Therapies Corp. Investigator's Brochure BTT12-CD011-S04 BTT1023 for further information).

For peer review only - http://bmjopen.bmj.com/site/about/guidelines.xhtml

BTT1023 is formulated at 20 mg/ml in a buffer containing 20 mM sodium citrate, 100 mM sodium chloride, 1.5% D-mannitol, 20 µM diethylenetriaminepentaacetic acid and 0.02% polysorbate 80 in water for injection; the pH is 7.0 ± 0.5.

BTT1023 is contained in single-use 10 mL clear type I glass vials with a white-coloured cap. Each vial is filled to the nominal volume of 5.0 mL of the sterile concentrate solution, thus each vial contains a nominal total of 100.0 mg of BTT1023.

BTT1023 requires dilution prior to administration by infusion. The amount of diluent to be added to the BTT1023 concentrate is calculated to consistently provide a total diluted drug product infusion volume of 50 ml. The amount of diluent required is based on the calculated dosage for each patient according to instructions provided to the administering clinicians.

The diluent product is designated as BTT1023 IV Infusion Diluent, 10 ml. It contains 0.9% sodium chloride and 0.02% polysorbate 80 in water for injection; the osmolality is 310 ± 30 mOsmol/kg, within the range for typical physiological value. It is contained in single-use 20 mL clear type I glass vials with a blue-coloured cap. Each vial is filled to the nominal volume of 10.0 mL of the sterile diluent solution.

7.1.2 Instructions for Storage

BTT1023 and its diluent are to be stored at 2-8°C.

7.1.3 Instructions for Handling the Investigational Medicinal Product

Since no preservative is present, it is recommended that the administration of the diluted solution for infusion is started as soon as possible after dilution. Due to a risk of microbial contamination the maximum shelf life for the diluted infusion solution is 24 hrs when stored at 2 to 8°C.

A 0.5-1 mL aliquot of the BTT1023 infusion solution will be taken at the end of the infusion period and stored at 2-8°C prior to dispatch to the bioanalytical laboratory. These aliquots will be labelled with the trial number, patient number and date of intravenous infusion. The samples will be stored at the bioanalytical laboratory (Envigo) and may be used for analysis of BTT1023 concentration if anomalies in PK data are observed that could be due to errors in IMP preparation. The samples will be discarded after the Clinical Study Report has been signed.

Further IMP details can be found in the current version of the BUTEO Pharmacy Manual.

7.2 Trial Treatment

This clinical trial is a single-arm clinical trial therefore all patients registered on to the study will receive the same treatment as detailed below in Table 3.

Treatment group: patients will receive multiple doses of BTT1023 (maximum of 7 doses) at 8, 12 or 16 mg/kg body weight.

For peer review only

Table 3: BTT1023 Drug Infusion Table

Visit number	Infusion number	Dose	Infusion time *
**3**	**1**	**8, 12 or 16 mg/kg**	120 minutes
**4-9**	**2-7**	**8, 12 or 16 mg/kg**	60 minutes

* Infusion rate for subsequent visits will only be increased if the individual patient did not experience a break in infusion due to AEs in the previous visit.

Patients will only receive one-dose level and no dose reductions will be allowed. If a patient experiences any events that require; either a SINGLE infusion to be interrupted, or result in an incomplete dose being given at any drug infusion visit then the patient will be assessed and the treating clinician will determine continuation of therapy based on an evaluation of the clinical reasons for interruption to therapy. If appropriate after discussion with the clinical coordinator and/or chief investigator a joint decision will be made if the patient should remain on the study and receive additional infusions at protocol defined scheduled visits. This decision will be fully documented in the individual patient medical record and site files as appropriate.

7.3 Treatment Schedule (Visit 3 – Visit 9)

Treatment Visit 3 (Day 1)

Pre-medication cetirizine 10 mg, ibuprofen 400 mg orally (in the absence of any contra-indication to non-steroidal anti-inflammatory drug [NSAIDs]), and 100mg hydrocortisone 1-2 hrs pre infusion

- Vital signs (heart rate, blood pressure, temperature, respiratory rate) pre-infusion
- Physical exam to be performed by clinician
- 12-lead ECG pre-infusion and 60 mins post infusion
- Clinical assessment to document clinical events and AEs
- Record of patient concomitant medication
- Quality of Life Questionnaires- EQ-5D, Fatigue Severity Scale, Pruritus Visual Analogue Score (VAS), collect IBD Diaries (if applicable) **(pre infusion)**
- BTT1023 infusion
- Vital signs (heart rate, blood pressure, temperature, respiratory rate) repeated at 30 and 60 mins after start of infusion, and 30, 60, 90, 120, 180, 240 mins post end of drug infusion
- Bloods for full blood count (FBC), international normalised ratio (INR), urea and electrolytes (UEs), liver function tests (LFTs), pre-infusion. See Table 4
- Blood samples (whole blood and serum) for biomarker and research blood analysis pre-infusion
- Bloods for FBC, INR, UE, LFT 2hrs post end of infusion period. See Table 4

For peer review only - http://bmjopen.bmj.com/site/about/guidelines.xhtml

- Blood serum for (PK (pre-infusion and then 15, 30, 60, 90, end of infusion, 180, 360 mins post infusion start. Some patients at the Birmingham site may have a 24 hr (post end of infusion) PK if they agree to this
- Blood serum for ADA (pre-infusion)
- Blood serum for PD (pre-infusion and then 180 and 360 minutes post infusion start). Some patients at the Birmingham site may have a 24 hr (post end of infusion) PD if they agree to this

Treatment Visit 4 (Day 8 +/- 3 days)

Pre-medication cetirizine 10 mg, ibuprofen 400 mg orally (in the absence of any contraindication to NSAIDs) and 100mg hydrocortisone 1-2 hrs pre infusion (See Section 7.9)

- Vital signs (heart rate, blood pressure, temperature, respiratory rate) pre-infusion
- 12-lead ECG pre-infusion and 60 mins post infusion
- Clinical assessment to document clinical events and AEs
- Record of patient concomitant medication
- Bloods for FBC, INR, UEs, LFTs pre-infusion. See Table 4
- Blood samples (whole blood and serum) for biomarker and research blood analysis pre-infusion
- Blood serum for PK (pre-infusion, end of infusion and then 60, 120 and 180 mins **post end of drug infusion**)
- Blood serum for PD (pre-infusion)
- BTT1023 infusion
- Vital signs (heart rate, blood pressure, temperature, respiratory rate) repeated at 30 and 60 mins after start of infusion, and 30, 60,120,180 mins post end of drug infusion
- Bloods for FBC, INR, UEs, LFTs 2hrs post end of infusion period. See Table 4

Treatment Visit 5 (Day 22+/- 3 days)

Pre-medication cetirizine 10 mg, ibuprofen 400 mg orally (in the absence of any contra-indication to NSAIDs) and 100mg hydrocortisone 1-2 hrs pre infusion (See Section 7.9)

- Pregnancy test (for females patients of childbearing potential only)
- Vital signs (heart rate, blood pressure, temperature, respiratory rate) pre-infusion
- Physical exam to be performed by clinician
- 12-lead ECG pre-infusion and 60 min post infusion
- Clinical assessment to document clinical events and AEs
- Record of patient concomitant medication
- Blood samples (whole blood and serum) for biomarker and research blood analysis pre-infusion
- Bloods for FBC, INR, UEs, LFTs pre-infusion. See Table 4
- BTT1023 infusion
- Vital signs (heart rate, blood pressure, temperature, respiratory rate) repeated at 30 and 60 mins after start of infusion, and 30, 90,120 mins post end of drug infusion
- Bloods for FBC, INR, UEs, LFTs 2hrs post end of infusion period. See Table 4
- Blood serum for PK (pre-infusion, end of infusion and 60 mins post end of drug infusion)

For peer review only - http://bmjopen.bmj.com/site/about/guidelines.xhtml

- Blood serum for PD (pre-infusion)

Treatment Visit 6 (Day 36 +/- 3 days)

Pre-medication cetirizine 10 mg + ibuprofen 400 mg orally (in the absence of any contra-indication to NSAIDs) 1-2 hrs pre infusion (See Section 7.9). 100mg hydrocortisone can be administered at the clinicians' discretion.

- Vital signs (heart rate, blood pressure, temperature, respiratory rate) pre-infusion
- 12-lead ECG pre-infusion and 60 mins post infusion
- Clinical assessment to document clinical events and AEs
- Record of patient concomitant medication
- Bloods for FBC, INR, UEs, LFTs pre-infusion. See Table 4
- Blood samples (whole blood and serum) for biomarker and research blood analysis pre-infusion
- Bloods for FBC, INR, UEs, LFTs 2hrs post end of infusion period. See Table 4
- Blood serum for PK (pre-infusion, end of infusion and 60 mins post end of drug infusion)
- Blood serum for PD (pre-infusion)
- BTT1023 infusion
- Vital signs (heart rate, blood pressure, temperature, respiratory rate) repeated at 30 and 60 mins after start of infusion, and 30, 90, 120 mins post end of drug infusion
- Quality of Life Questionnaires- EQ-5D, Fatigue Severity Scale, Pruritus Visual Analogue Score (VAS), IBD Diaries (if applicable)

Treatment Visit 7 (Day 50+/- 3 days)

Pre-medication cetirizine 10 mg + ibuprofen 400 mg orally (in the absence of any contra-indication to NSAIDs) 1-2 hrs pre infusion (See Section 7.9). 100mg hydrocortisone can be administered at the clinicians' discretion.

- Pregnancy test (for females patients of childbearing potential only)
- Vital signs (heart rate, blood pressure, temperature, respiratory rate) pre-infusion
- Physical exam to be performed by clinician
- 12-lead ECG pre-infusion and 60 mins post infusion
- Clinical assessment to document clinical events and AEs
- Record of patient concomitant medication
- Bloods for FBC, INR, UEs, LFTs pre-infusion. See Table 4
- Blood samples (whole blood and serum) for biomarker and research blood analysis pre-infusion
- Blood serum for PK (pre-infusion, end of infusion and 60 mins post end of drug infusion)
- Blood serum for PD (pre-infusion)
- Blood serum for ADA (pre-infusion)
- BTT1023 infusion
- Vital signs (heart rate, blood pressure, temperature, respiratory rate) repeated at 30 and 60 mins after start of infusion, and 30, 90,120 mins post end of drug infusion
- Bloods for FBC, INR, UEs, LFTs 2hrs post end of infusion period. See Table 4

For peer review only - http://bmjopen.bmj.com/site/about/guidelines.xhtml

Treatment Visit 8 (Day 64 +/- 3 days)

Pre-medication cetirizine 10 mg + ibuprofen 400 mg orally (in the absence of any contra-indication to NSAIDs) 1-2 hrs pre infusion (See Section 7.9). 100mg hydrocortisone can be administered at the clinicians' discretion.

- Vital signs (heart rate, blood pressure, temperature, respiratory rate) pre-infusion
- 12-lead ECG pre-infusion and 60 mins post infusion
- Clinical assessment to document clinical events and AEs
- Record of patient concomitant medication
- Bloods for FBC, INR, UEs, LFTs pre-infusion. See Table 4
- Blood samples (whole blood and serum) for biomarker and research blood analysis pre-infusion
- Blood serum for PK (pre-infusion, end of infusion and 60 mins post end of drug infusion)
- Blood serum for PD (pre-infusion)
- BTT1023 infusion
- Vital signs (heart rate, blood pressure, temperature, respiratory rate) repeated at 30 and 60 mins after start of infusion, and 30, 90,120 mins post end of drug infusion
- Bloods for FBC, INR, UEs, LFTs 2 hrs post end of infusion period. See Table 4

Treatment Visit 9 (Day 78 +/- 3 days)

Pre-medication cetirizine 10 mg + ibuprofen 400 mg orally (in the absence of any contra-indication to NSAIDs) 1-2 hrs pre infusion (See Section 7.9). 100mg hydrocortisone can be administered at the clinicians discretion.

- Pregnancy test (for females patients of childbearing potential only)
- Vital signs (heart rate, blood pressure, temperature, respiratory rate) pre-infusion
- Physical exam to be performed by clinician
- 12-lead ECG pre-infusion and 60 mins post infusion
- Clinical assessment to document clinical events and AEs
- Record of patient concomitant medication
- Bloods for FBC, INR, UEs, LFTs pre-infusion. See Table 4
- Blood samples (whole blood and serum) for biomarker and research blood analysis pre-infusion
- Blood serum for PK (pre-infusion and then 15, 30, end of infusion 120 mins post infusion start, and end of infusion). Some patients at the Birmingham site may have a 24 hr PK if they agree to this
- Blood serum for ADA (pre-infusion)
- Blood serum for PD (pre-infusion and then 120 minutes post infusion start). Some patients at the Birmingham site may have a 24 hr (post end of drug infusion) PD if they agree to this
- BTT1023 infusion
- Vital signs (heart rate, blood pressure, temperature, respiratory rate) repeated at 30 and 60 mins after start of infusion, and 30, 90,120 mins post end of drug infusion
- Bloods for FBC, INR, UEs, LFTs 2 hrs post end of infusion period. See Table 4

For peer review only - http://bmjopen.bmj.com/site/about/guidelines.xhtml

7.4 Trial Schema

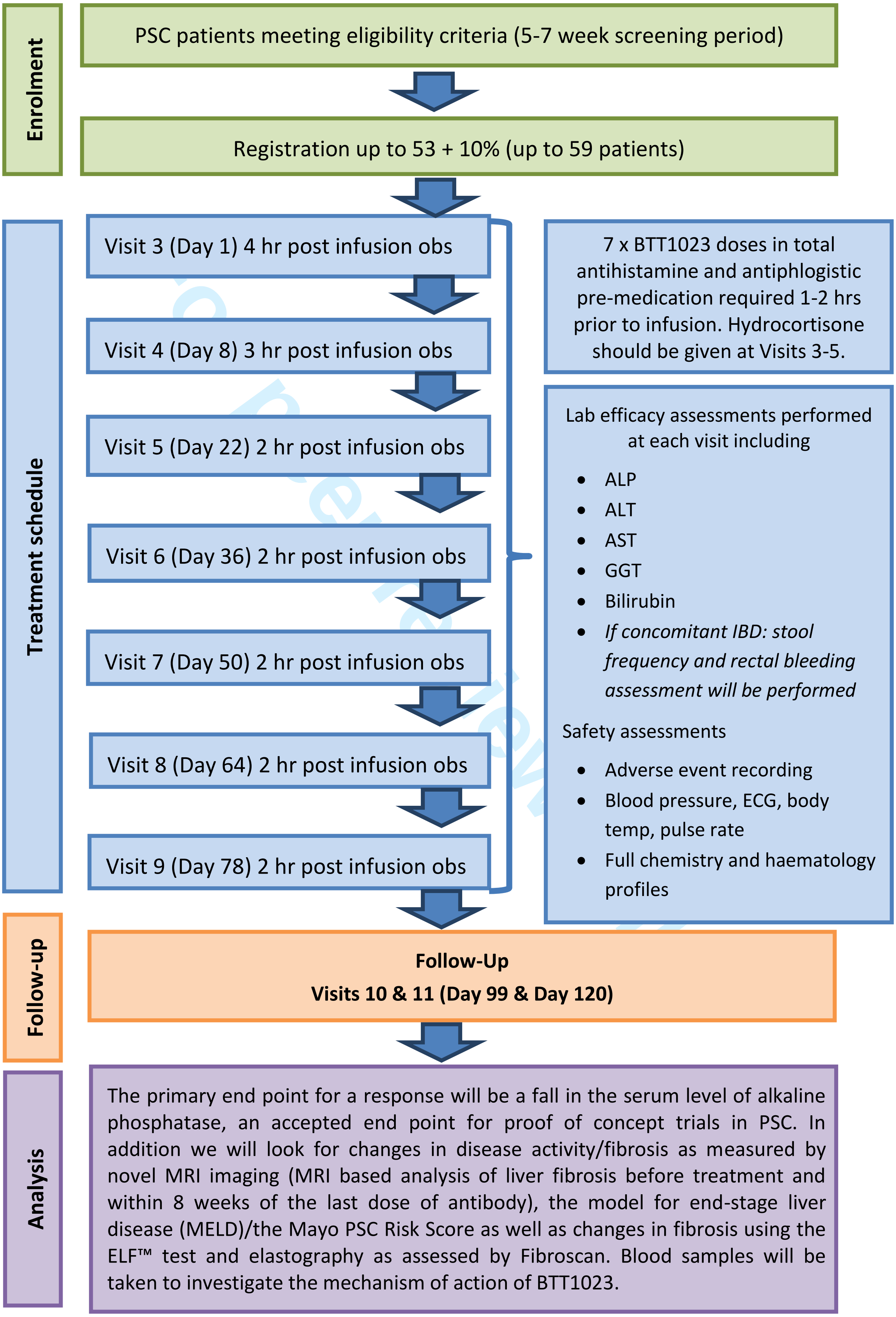

Figure 2: Study/Patient Flow Diagram

For peer review only - http://bmjopen.bmj.com/site/about/guidelines.xhtml

7.5 Assessments

7.5.1 Blood Chemistry and Haematology

The following blood tests will be performed at each visit. Tests used for activity assessment will be performed in a central laboratory, whereas safety laboratory assessments will be done in local laboratories at investigative sites.

Table 4: Blood Tests

	Blood test	Primary reason for assessment
**Biochemistry**	Alkaline Phosphatase (ALP) - central analysis	Activity
	Alkaline Phosphatase (ALP) - local analysis	Safety
	Alanine Transaminase (ALT)	
	Aspartate Transaminase (AST)	
	Albumin	
	Bilirubin (direct and indirect if total is ≥ 30)	
	Gamma Glutamyl Transferase (GGT)	
	Sodium	Safety
	Potassium	
	Urea	
	Creatinine	
	Calcium	
	Total Protein	
	*Estimated Glomerular Filtration Rate (eGFR)	
**Haematology**	Haemoglobin	Safety
	Platelets	
	Red Blood Cells	
	White Blood Cells	
	Haematocrit	
	Mean Cell Volume	
	Mean Cell Haemoglobin	
	Neutrophils	
	Lymphocytes	
	Monocytes	

For peer review only

	Eosinophils	
	Basophils	
	INR	
	APTT Ratio	

*- calculated by Cockcroft-Gault equation or alternative as per institutional practice – Appendix 1

Only in the event of suspected adverse event: Cytokine release syndrome the following blood tests will need to be performed to confirm adverse event. These tests are considered as part of standard care when investigating this specific syndrome. Please see the Laboratory Manual for further details.

Table 5: Immunology Blood Tests

**Immunology**	TNF- α	Safety
	IFN-ϒ	
	IL-6	

7.5.2 Pharmacokinetics Scheduling

Table 6: Visit 3+9 Pharmacokinetics Scheduling

Visit 3 (Day 1) + Visit 9 (Day 78 +/- 3 Days)

**Time point**	**Blood sample type**
Pre-infusion	Blood serum
15 minutes post infusion start	Blood serum
30 minutes post infusion start	Blood serum
60 minutes post infusion start*	Blood serum
90 minutes post infusion start*	Blood serum
End of infusion	Blood serum
180 minutes** (120 minutes) post infusion start	Blood serum
360 minutes** post infusion start	Blood serum
24 hrs post end of infusion time #	Blood serum

will only be performed on a sub-set of patients (treated at the Queen Elizabeth Hospital Birmingham) and only if the individual patients agree.

*due to the length of the infusion (60 minute) at infusion Visit 9 this time point will not be required.

**at infusion Visit 9 patients only remain for a maximum of 2 hrs post infusion so these PK time points will be ended at 120 minutes on the day of infusion.

For peer review only

Table 7: Visit 4 Pharmacokinetics Scheduling

Visit 4 (Day 8 +/- 3 Days)

Time point	Blood sample type
Pre-infusion	Blood serum
End of infusion	Blood serum
60 minutes post end of infusion	Blood serum
120 minutes post end of infusion	Blood serum
180 minutes post end of infusion	Blood serum

Table 8: Visit 5-8 Pharmacokinetic Scheduling

Visit 5 (Day 22 +/- 3 Days), Visit 6 (Day 36 +/- 3 Days), Visit7 (Day 50 +/- 3 Days), Visit 8 (Day 64 +/- 3 Days)

Time point	Blood sample type
Pre-infusion	Blood serum
End of infusion	Blood serum
60 minutes post end of infusion	Blood serum

7.5.3 Physical Examination/Symptom Assessment

Physical assessments will be performed by the trial clinician and include height (to be performed at Visit 1 only) and weight. A physical examination (including general appearance, respiratory, cardiovascular, skin, head and neck, lymph nodes, thyroid, abdomen, musculo-skeletal) should be performed at Screening Visit 1, prior to infusions during Visits 3, 5, 7 and 9 and during follow up Visits 10 and 11.

7.5.4 Mayo Risk Assessment

The patient's age, bilirubin, albumin, AST and history of variceal bleeding will be used to calculate the Mayo PSC Risk score at Visit 2 [-1 week screening visit] and Visit 10 [Day 99]. The following link can be used:

http://www.mayoclinic.org/medical-professionals/model-end-stage-liver-disease/revised-natural-history-model-for-primary-sclerosing-chonalgitis

For peer review only - http://bmjopen.bmj.com/site/about/guidelines.xhtml

7.6 Sample Collection

7.6.1 Blood Samples

The following samples will be collected during the trial:

1. Standard blood tests - FBC, INR, UEs, LFTs
2. Blood and serum sample for biomarker and research blood analysis
3. Blood serum for PK
4. Blood serum for ADA
5. Blood serum for PD (sVAP-1)
6. Blood for T-SPOT.TB test *[screening visit 1 only]*

7.6.2 Pharmacokinetic, Anti-Drug Antibodies and Pharmacodynamic Samples

For all patients, blood samples for the determination of BTT1023, ADA and sVAP-1 will be collected as is scheduled in the Schedule of Events table. Please see the latest version of the laboratory manual for further information.

7.7 Dose Modifications

No patient dose modifications or dose reductions will be allowed during the dose confirmation phase of the trial. In the phase II component of the trial, dose reduction or modifications may be considered at the discretion of the Chief Investigator. Please contact the BUTEO Trials Office to discuss further. The dose should not be reduced or modified until written permission from the Chief Investigator has been received.

7.8 Treatment Compliance

The local hospital will keep drug accountability records that can be reviewed by sponsor representatives.

Please refer to the current BUTEO Pharmacy Manual for further details.

7.9 Pre-Medication

The pre-medication is cetirizine 10 mg + ibuprofen 400 mg orally (in the absence of any contra-indication to NSAIDs) about 1-2 hrs before the start of every infusion of BTT1023. Additionally, all patients should be given 100mg hydrocortisone 1-2 hours prior to the infusions at Visits 3-5. Hydrocortisone can be administered at subsequent infusions at the clinicians' discretion. In case of intolerance/allergy to hydrocortisone or Cetirizine, please contact the BUTEO Trials office as confirmation with the Clinical Coordinator or Medical monitor prior to infusion is required. If there is a delay in administering the BTT1023 infusion consider whether this is delay is sufficient enough to warrant further pre-medication.

7.10 Concomitant Medication

All medication that each participant is taking at the time, or within 3 months, of enrolment will be recorded. New medications or changes to current medications during the trial will also be recorded.

The pharmaceutical/trade name, dose, route of administration, indication, start/stop date of each new medication within the trial will be recorded. Any drug that is licensed within the United Kingdom

For peer review only - http://bmjopen.bmj.com/site/about/guidelines.xhtml

and Europe, that is deemed necessary for the participant's health-care, will be permitted at the discretion of the Chief Investigator. The exceptions to this include:

- Biological drugs (i.e. monoclonal antibodies) other than the investigational product
- Initiation during the study period of any therapy that has the potential to change serum alkaline phosphatase, e.g. high dose steroids, methotrexate, fenofibrate, ursodeoxycholic acid (UDCA) or related agents
- In those patients receiving UDCA in compliance with the inclusion criteria, changes in UDCA dosage are not permitted during the study period

7.11 Contraception and Pregnancy

All sexually active women of childbearing potential must agree to use a highly effective method of contraception from the Screening Visit throughout the study period and for 99 days following the last dose of study drug. If using hormonal agents the same method must have been used for at least 1 month before study dosing and subjects must use a barrier method as the other form of contraception. Lactating women must agree to discontinue breast feeding before study investigational medicinal product administration.

For the purposes of this study, a female subject of childbearing potential is a woman who has not had a hysterectomy, bilateral oophorectomy, or medically-documented ovarian failure. Women ≤ 50 years of age with amenorrhea of any duration will be considered to be of childbearing potential.

Men, if not vasectomised, must agree to use barrier contraception (condom plus spermicide) during heterosexual intercourse from screening through to study completion and for 99 days from the last dose of study investigational medicinal product.

Refer to section 8 if a pregnancy is reported, either that of a female participant, or the partner of a male participant.

7.12 Patient Follow Up

Visit 10*: End of Treatment Visit (Day 99 +/- 3 days)

- Vital signs (heart rate, blood pressure, temperature, respiratory rate)
- Physical exam (to be performed by clinician)
- Clinical assessment to document clinical events and AEs
- Record of patient concomitant medication
- Bloods for FBC, INR, UEs, LFTs
- Blood serum (PK, ADA and PD)
- Blood sample (whole blood and serum) for biomarker and research blood analysis
- Fibroscan
- ECG
- Quality of Life Questionnaires- EQ-5D, Fatigue Severity Scale, Pruritus Visual Analogue Score (VAS), IBD Diaries (if applicable)

Visit 11: End of Treatment Visit (Day 120 +/- 3 days)

- Vital signs (heart rate, blood pressure, temperature, respiratory rate)

For peer review only - http://bmjopen.bmj.com/site/about/guidelines.xhtml

- Physical exam (to be performed by clinician)
- Clinical assessment to document clinical events and adverse events
- Record of patient concomitant medication. Bloods for FBC, INR, UEs, LFTs
- Blood serum (PK, ADA and PD)
- Blood sample (whole blood and serum) for biomarker and research blood analysis
- MRI within 4-6 weeks after the last treatment (Visit 9) or last dose of antibody*

* If a patients stops treatment early this visit will be 21 days (+/-3 days) after the last dose of BTT11023 with the MRI within 8 weeks of last dose of BTT11023

NB: All visit dates / time are related to Visit 3 (Day 1). If a visit is delayed for any reason the next visit date will be scheduled in relation to Visit 3. There are no planned treatment delays. If a drug infusion visit cannot be performed within the required timeframe, every attempt should be made to administer the infusion as close as possible to the permitted time window. In extreme situations, protocol-scheduled visits may be cancelled and declared as missed visits at the clinical discretion of the Investigator.

7.13 Patient Withdrawal

The Investigator will make every reasonable effort to keep each patient on treatment. However, if the Investigator removes a patient from the study treatment or if (s)he declines further participation final assessments will be performed, if possible. All the results of the evaluations and observations, together with a description of the reasons for study withdrawal, must be recorded in the CRF.

In the event of a patient's decision to withdraw from the trial, the Investigator must ascertain from which aspects of the trial the patient wishes to withdraw, and record the details on the appropriate CRF. All patients will continue to be followed-up (if they agree), and all information and tissue samples collected up until the point of retraction, will be retained and analysed.

Patients who are removed from treatment due to adverse events (clinical or laboratory) will be treated and followed according to accepted medical practice. All pertinent information concerning the outcome of such treatment must be recorded in the CRF.

The following are justifiable reasons for the Investigator to withdraw a patient from study:

- Unacceptable toxicity
- Unforeseen events - any event which in the judgement of the Investigator makes further treatment inadvisable
- SAE requiring discontinuation of treatment
- Withdrawal of consent
- Serious violation of the study protocol (including persistent patient attendance failure and persistent non-compliance)
- Withdrawal by the Investigator for clinical reasons not related to the study drug treatment

The maximum potential sample size, 59, has been calculated to cover patient withdrawals.

For peer review only - http://bmjopen.bmj.com/site/about/guidelines.xhtml

8. ADVERSE EVENT REPORTING

The collection and reporting of AEs will be in accordance with the Medicines for Human Use Clinical Trials Regulations 2004 and its subsequent amendments. Definitions of different types of AE are listed in Appendix 4. The Investigator should assess the seriousness and causality (relatedness) of all AEs experienced by the patient (this should be documented in the source data) with reference to the current BTT1023 Investigator Brochure.

8.1 Reporting Requirements

All AEs, whether serious or not, will be recorded throughout the study. At each contact with the site, the patient will be asked about AEs. All AEs, either observed by the Investigator or reported by the subject, will be recorded by the Investigator and evaluated.

Collection, Recording and Reporting of Adverse Events:

In line with the Medicines for Human Use (Clinical Trials) Regulations 2004, an accurate and up to date record of all AEs reported by investigators will be maintained throughout the trial. This record will include details of nature, onset, duration, severity, outcome and any relationship to the investigational product. The Sponsor, appropriate regulatory authority (e.g. Medicines and Healthcare Products Regulatory Agency (MHRA)) and Research Ethics Committee will be informed as required by current regulations.

The NCI Common Terminology Criteria for Adverse Events (CTCAE) Version 4.0 will be used to grade each AE. In the event of the specific AE term not being available in the CTCAE the associated body system 'AE term –other' will be used and the event graded in accordance with guidelines i.e.: Grade 1: mild, Grade 2: Moderate, Grade 3: severe, Grade 4: life-threatening and grade 5: Death. As determined by the treating clinician.

A non-leading, open question will be asked initially to evaluate for possible AEs. Examples include:

At screening (Visit 1-2) - 'Are you experiencing any symptoms?'

At subsequent visits (Visits 3 – 11) – 'How have you been since your last visit?'

As a minimum requirement, all Serious Adverse Events (SAEs) will be reported to Biotie Therapies. Events such as medication errors and suspected transmission of an infectious agent via a trial product will always be considered as medical events of special interest and will be reported to Biotie Therapies irrespective of seriousness. These events will need to reported as an SAE.

Pre-existing conditions

Any pre-existing conditions should be reported as medical history, and should not be reported as an AE unless the condition worsens by at least one CTC grade during the trial. The condition, however, must be reported in the CRF.

8.1.1 Adverse Events

All medical occurrences which meet the definition of an AE (see Appendix 4 for definition) should be reported. Please note this includes all abnormal laboratory findings, including those that are not clinically significant.

For peer review only - http://bmjopen.bmj.com/site/about/guidelines.xhtml

8.1.2 Hypersensitivity, Infusion Reactions and Infusion Related Reactions

8.1.2.1 Hypersensitivity and Infusion Reactions

Hypersensitivity reactions are defined according to the NCI-CTCAE Version 4.0 definition of allergic reaction / hypersensitivity, as follows:

Grade 1: transient flushing or rash, drug fever <38°C

Grade 2: rash, flushing, urticaria, dyspnoea, drug fever ≥38°C

Grade 3: symptomatic bronchospasm, requiring parenteral medication(s), with or without urticaria; allergy-related oedema/angioedema, hypotension

Grade 4: anaphylaxis (a life-threatening event characterized by the rapid onset [often within minutes] of airway obstruction [bronchospasm, stridor, hoarseness], urticaria, and/or hypotension)

8.1.2.2 Infusion Related Reactions

Infusion-related reactions are defined according to the NCI-CTCAE Version 4.0 definition, as follows:

Grade 1: mild, transient reaction, infusion interruption not indicated; intervention not indicated

Grade 2: therapy or infusion interruption indicated but responds promptly to symptomatic treatment (for example, antihistamines, non-steroidal anti-inflammatory drugs [NSAIDS], narcotics, I.V. fluids); prophylactic medications indicated for ≤24 hours

Grade 3: Prolonged (for example, not rapidly responsive to symptomatic medication and/or brief interruption of infusion); recurrence of symptoms following initial improvement; hospitalisation indicated for clinical sequelae

Grade 4: Life-threatening consequences; urgent intervention indicated consistent with usual medical practice, selected parenteral medications may be utilised as detailed below. Additional treatments, chosen according to clinical symptoms and local standards, may be utilised at investigator discretion.

8.1.3 Serious Adverse Events

Investigators should report AEs that meet the definition of an SAE (see Appendix 4 for definition).

8.1.3.1 Monitoring Pregnancies for Potential Serious Adverse Events

It is important to monitor the outcome of pregnancies of patients in order to provide SAE data on congenital anomalies or birth defects.

In the event that a patient or their partner becomes pregnant during the SAE reporting period please complete a Pregnancy Notification Form (providing the patient's details) and return to the BUTEO Trials Office as soon as possible. If it is the patient who is pregnant provide outcome data on a follow-up Pregnancy Notification Form. Where the patient's partner is pregnant consent must first be obtained and the patient should be given a Pregnancy Release of Information Form to give to their partner. If the partner is happy to provide information on the outcome of their pregnancy they should sign the Pregnancy Release of Information Form. Once consent has been obtained provide details of the outcome of the pregnancy on a follow-up Pregnancy Notification Form. If appropriate also complete an SAE Form as detailed below.

8.1.4 Reporting Period

The reporting period for AEs will commence from the date of consent, (typically Visit 1: screening visit) and will continue until the follow-up visit (Visit 11: Day 120), or alternatively up to 45 days post

For peer review only - http://bmjopen.bmj.com/site/about/guidelines.xhtml

last study drug infusion if the patient withdraws from the study prior to completion of all 7 study drug infusions.

The reporting period for SAE will commence from the date of consent, (typically Visit 1: screening visit) and will continue until the follow-up visit (Visit 11: Day 120), or alternatively up to 45 days post last study drug infusion if the patient withdraws from the study prior to completion of all 7 study drug infusions.

SAEs that are judged to be at least possibly related to the IMP must still be reported in an expedited manner irrespective of how long after IMP administration the reaction occurred.

8.2 Reporting Procedure

8.2.1 Site

8.2.1.1 Adverse Events

AEs should be reported on an AE Form (and where applicable on an SAE Form). AEs should be completed at each visit and the information recorded on the case report form (eRDC) /or paper form as appropriate.

In the event of the specific AE term not being available in the CTCAE the associated body system 'AE term –other' will be used and the event graded in accordance with guidelines i.e.: Grade 1: mild, Grade 2: Moderate, Grade 3: severe, Grade 4: life-threatening and grade 5: Death. As determined by the treating clinician.

8.2.1.2 Serious Adverse Events

For more detailed instructions on SAE reporting refer to the SAE Form Completion Guidelines section of the ISF.

AEs defined as serious and which require reporting as an SAE should be reported on an SAE Form. When completing the form, the Investigator will be asked to define the causality and the severity of the AE which should be documented using the CTCAE version 4.0.

On becoming aware that a patient has experienced an SAE, the Investigator (or delegate) must complete, date and sign an SAE Form. The form should be faxed together with a SAE Fax Cover Sheet to the Trials Office using one of the numbers listed below as soon as possible and no later than 24 hrs after first becoming aware of the event:

To report an SAE, fax the SAE Form with an SAE Fax Cover Sheet to:

0121 414 8286 (primary number)

0121 414 3529 (secondary number)

On receipt the BUTEO Trials Office will allocate each SAE a unique reference number. This number will be transcribed onto the SAE Fax Cover Sheet which will then be faxed back to the site as proof of receipt. If confirmation of receipt is not received within 1 working day please contact the Trials Office. The SAE reference number should be quoted on all correspondence and follow-up reports

For peer review only - http://bmjopen.bmj.com/site/about/guidelines.xhtml

regarding the SAE. The SAE Fax Cover Sheet completed by the BUTEO Trials Office should be filed with the SAE Form in the ISF.

For SAE Forms completed by someone other than the Investigator, the Investigator will be required to countersign the original SAE Form to confirm agreement with the causality and severity assessments. The form should then be returned to the BUTEO Trials Office in the post and a copy kept in the ISF.

Investigators should also report SAEs to their own Trust in accordance with local practice.

8.2.1.3 Provision of Follow-up Information

Patients should be followed up until resolution or stabilisation of the event. Follow-up information should be provided on a new SAE Form (refer to the SAE Form Completion Guidelines for further information).

8.2.2 Trials Office

On receipt of an SAE Form seriousness and causality will be determined independently by a Clinical Coordinator. An SAE judged by the Investigator or Clinical Coordinator to have a reasonable causal relationship with the trial medication will be regarded as a Serious Adverse Reaction (SAR). The Clinical Coordinator will also assess all SARs for expectedness. If the event meets the definition of a SAR that is unexpected (i.e. is not defined in the Investigator Brochure) it will be classified as a Suspected Unexpected Serious Adverse Reaction (SUSAR).

8.2.3 Reporting to the Competent Authority and Research Ethics Committee

8.2.3.1 Suspected Unexpected Serious Adverse Reactions

The BUTEO Trials Office will report a minimal data set of all individual events categorised as a fatal or life threatening SUSAR to the Medicines and Healthcare products Regulatory Agency (MHRA) and Research Ethics Committee (REC) within 7 days. Detailed follow-up information will be provided within an additional 8 days.

All other events categorised as SUSARs will be reported within 15 days.

Concurrently with the above, the trials office will report all such events to Biotie Therapies nominated safety representative (IMP provider).

8.2.3.2 Serious Adverse Reactions

The BUTEO Trials Office will report details of all SARs (including SUSARs) to the MHRA and REC annually from the date of the Clinical Trial Authorisation, in the form of a Developmental Safety Update Report Adverse Events.

Details of all AEs will be reported to the MHRA on request.

8.2.3.3 Other Safety Issues Identified During the Course of the Trial

The MHRA and REC will be notified immediately if a significant safety issue is identified during the course of the trial.

For peer review only

8.2.4 Investigators

Details of all SUSARs and any other safety issue which arises during the course of the trial will be reported to Principal Investigators. A copy of any such correspondence should be filed in the ISF.

8.2.5 Data Monitoring Committee

The independent Data Monitoring Committee (DMC) will review all SAEs.

8.2.6 Provider of Investigational Medicinal Product

All SAEs will be reported to the provider (Biotie Therapies) of the Investigational Medicinal Product (BTT1023) within 24 hrs by email.

8.2.7 Reporting to the Research Ethics Committee

8.2.7.1 Unexpected and Related Serious Adverse Events

The BUTEO Trials Office will report all events categorised as Unexpected and Related SAEs to the Research Ethics Committee (REC) within 15 days.

8.2.7.2 Other Safety Issues Identified During the Course of the Trial

The REC will be notified immediately if a significant safety issue is identified during the course of the trial.

9. DATA HANDLING AND RECORD KEEPING

9.1 Data Collection

Data will be collected during this clinical trial. Data will be captured on case report forms (CRF), initially this will be on paper forms. Once the electronic remote data capture (eRDC) systems is implemented sites will be informed and further details provided. The Case Report Form (paper CRF/eRDC) is listed in Table 9 below.

Table 9: Description of Case Report Forms

Form	Summary of data recorded	Schedule for submission
Eligibility Checklist	Confirmation of eligibility and satisfactory staging investigations where necessary	Faxed at point of registration.
Registration	Patient details and Registration Number	Faxed at point of registration.
**Dosing Forms**		
BTT1023 Dosing	Dose level, Visit, Date, Dosing details	Within 2 weeks
**Sample Analysis**		

For peer review only - http://bmjopen.bmj.com/site/about/guidelines.xhtml

Forms		
Pharmacokinetics	Date, Visit, Sample collection, time taken	Within 2 weeks
Pharmacodynamic	Date, Visit, Sample collection, time taken	Within 2 weeks
Biomarker Sample	Date, Visit, Sample collection, time taken	Within 2 weeks
ADA	Date, Visit, Sample collection, time taken	Within 2 weeks
Research Sample	Date, Visit, Sample collection, time taken	Within 2 weeks
**Assessment Forms**		
Adverse Event	Start and stop dates, grade according to CTCAE version 4.0, causality	Every 4 weeks and as requested
Concomitant Medication	List of concomitant medication	Every 4 weeks and as requested
Biochemistry (Screening, Pre Dose & Post Dose Treatment, Follow Up)	Date, Visit, results, clinical significance	Within 2 weeks
ECG	Date, Visit, results	Within 2 weeks
Haematology	Date, Visit, results, clinical significance	Within 2 weeks
Medical History	Prior conditions, diagnosis, grade	Within 2 weeks
Previous Treatment	Previous Treatment start/ end date, treatment type, dose and schedule if applicable	Within 2 weeks
Ethnicity	Date, Visit, Ethnicity	Within 2 weeks
Physical Examination	Date, Visit, investigations	Within 2 weeks
Pregnancy Test	Date, Visit, result	Within 2 weeks
Vital Signs	Date, Visit, pre- post dose time, results	Within 2 weeks
Mayo Risk Score	Date, Visit, results required for Mayo Risk Score Calculation, Mayo	Within 2 weeks

For peer review only

	Risk Score	
MRI	Date, Visit, Results	Within 2 weeks
Meld Score	Date, Visit, Results	Within 2 weeks
T-Spot TB Test	Date, Visit, Results	Within 2 weeks
Fibroscan	Date, Visit, Results	Within 2 weeks
**Ad Hoc Forms**		
Treatment Discontinuation	As required	Within 4 weeks of study visit date.
Serious Adverse Event Form	Details of the SAE	Within 24 hrs of being made aware of the SAE
Pregnancy Notification Form	Patient details and details of pregnancy	Within 24 hrs of being made aware of the pregnancy
Death Form	Date and cause of death	Immediately upon notification of death
Deviation Form	Completed in the event of a deviation from the protocol	Immediately upon discovering deviation.
Withdrawal Form	Used to notify the Trials Office of patient withdrawal from the trial	Immediately upon patient withdrawal
Quality of Life Forms	Patient response to QOL questions	Within 4 weeks of study visit date

The CRF must be completed, signed/dated (for paper CRFs only) and returned to the BUTEO Trials Office by the Investigator or an authorised member of the site research team (as delegated on the Site Signature and Delegation Log) within the timeframe listed above entries on the CRF should be made in ballpoint pen, in blue or black ink, and must be legible. Any errors should be crossed out with a single stroke, the correction inserted and the change initialled and dated. If it is not obvious why a change has been made, an explanation should be written next to the change.

Data reported on each form should be consistent with the source data or the discrepancies should be explained. If information is not known, this must be clearly indicated on the form. All missing and ambiguous data will be queried. All sections are to be completed before returning.

Data provided on the Quality of Life Forms will not be captured on source data. This data will be written directly onto the CRF's.

In all cases it remains the responsibility of the Investigator to ensure that the CRF has been completed correctly and that the data are accurate.

The completed originals should be sent to the BUTEO Trials Office and a copy filed in the ISF.

Trial forms may be amended by the BUTEO Trials Office, as appropriate, throughout the duration of the trial. Whilst this will not constitute a protocol amendment, new versions of the form must be implemented by participating sites immediately on receipt.

For peer review only

9.2 Archiving

It is the responsibility of the Principal Investigator to ensure all essential trial documentation and source records (e.g. signed ICFs, ISFs, Pharmacy Files, patients' hospital notes, copies of CRFs etc.) at their site are securely retained for at least 15 years after the end of the trial or following the processing of all biological material collected for research, whichever is the later. Do not destroy any documents without prior approval from the CRCTU Document Storage Manager.

10. QUALITY MANAGEMENT

The BUTEO clinical trial in patients with PSC is being conducted under the auspices of the Cancer Research UK Clinical Trials Unit (CRCTU) according to the current guidelines for Good Clinical Practice (GCP). Participating sites will be monitored by CRCTU staff to confirm compliance with the protocol and the protection of patients' rights as detailed in the Declaration of Helsinki: October 1996 (Appendix 5).

10.1 Site Set-up and Initiation

All sites will be required to sign a Clinical Study Site Agreement prior to participation. In addition all participating Investigators will be asked to sign the necessary agreements, Investigator Registration Forms and supply a current CV and GCP to the BUTEO Trials Office. All members of the site research team will also be required to sign the Site Signature and Delegation Log, which should be returned to the BUTEO Trials Office. Prior to commencing recruitment all sites will undergo a process of initiation. Key members of the site research team will be required to attend either a meeting or a teleconference covering aspects of the trial design, protocol procedures, AE reporting, collection and reporting of data and record keeping. Sites will be provided with an ISF and a Pharmacy File containing essential documentation, instructions, and other documentation required for the conduct of the trial. The BUTEO Trials Office must be informed immediately of any change in the site research team.

10.2 On-site Monitoring

Monitoring will be carried out as required following a risk assessment and as documented in the BUTEO Quality Management Plan. Additional on-site monitoring visits may be triggered for example by poor CRF return, poor data quality, low SAE reporting rates, excessive number of patient withdrawals or deviations. If a monitoring visit is required the Trials Office will contact the site to arrange a date for the proposed visit and will provide the site with written confirmation. Investigators will allow the BUTEO trial staff access to source documents as requested.

10.3 Central Monitoring

Trials staff will be in regular contact with the site research team to check on progress and address any queries that they may have. Trials staff will check incoming CRFs for compliance with the protocol, data consistency, missing data and timing. Sites will be sent Data Clarification Forms (DCFs) requesting missing data or clarification of inconsistencies or discrepancies.

Sites may be suspended from further recruitment in the event of serious and persistent non-compliance with the protocol and/or GCP, and/or poor recruitment. Any major problems identified during monitoring may be reported to the Trial Management Group and the Trial Steering Committee and the relevant regulatory bodies. This includes reporting serious breaches of GCP

For peer review only - http://bmjopen.bmj.com/site/about/guidelines.xhtml

and/or the trial protocol to the main Research Ethics Committee (REC) and the Medicines for Healthcare products Regulatory Agency (MHRA).

10.4 Audit and Inspection

The Investigator will permit trial-related monitoring, audits, ethical review, and regulatory inspection(s) at their site, providing direct access to source data/documents.

Sites are also requested to notify the BUTEO Trials Office of any MHRA inspections.

10.5 Notification of Serious Breaches

In accordance with Regulation 29A of the Medicines for Human Use (Clinical Trials) Regulations 2004 and its amendments, the Sponsor of the trial is responsible for notifying the licensing authority in writing of any serious breach of:

- The conditions and principles of GCP in connection with that trial or;
- The protocol relating to that trial, within 7 days of becoming aware of that breach

For the purposes of this regulation, a “serious breach” is a breach which is likely to effect to a significant degree:

- The safety or physical or mental integrity of the subjects of the trial; or
- The scientific value of the trial

Sites are therefore requested to notify the Trials Office of a suspected trial-related serious breach of GCP and/or the trial protocol. Where the Trials Office is investigating whether or not a serious breach has occurred sites are also requested to cooperate with the Trials Office in providing sufficient information to report the breach to the MHRA where required and in undertaking any corrective and/or preventive action.

11. END OF TRIAL DEFINITION

The end of trial will be the date of Last Patient Last Visit (LPLV). The BUTEO Trials Office will notify the MHRA and REC that the trial has ended and will provide them with a summary of the clinical trial report within 12 months of the end of trial.

After closure of the trial with the MHRA, the Sponsor is no longer required to notify the MHRA and REC of changes of Principal Investigator. However, sites should continue to notify the BUTEO Trials Office of changes in Principal Investigator by completing and returning (where required) an Investigator Registration Form together with a current signed and dated CV.

The trial may close early if stage-1 continuation criterion (detailed in Section 12) is not met.

12. STATISTICAL CONSIDERATIONS

No specific toxicities are expected with the BTT1023 treatment however, a Dose Limiting Toxicity is defined as an AE that meets the criteria of grade 4 or grade 5 as defined in the Common terminology criteria for adverse events (CTCAE) (Appendix 2). However, if a definite diagnosis of cytokine release syndrome is made, it will be classed as a DLT at grade 3 and above* and must be considered at least possibly related to BTT1023.

For peer review only - http://bmjopen.bmj.com/site/about/guidelines.xhtml

If the trough levels of circulating BTT1023 are found to be <3 µg/ml, this indicates that a therapeutic level has not been reached and either the dose will escalate or the trial will stop. Details of the possible dose-pathways have been described in Section 3.

12.1 Definition of Outcome Measures

12.1.1 Primary Outcome Measure

Response at Day 99: a reduction in serum ALP by 25% from baseline to Day 99. This is a binary indicator and is based on the change being -25% (a negative value indicates a reduction). The calculation method and formula can be found in the Statistical Analysis Plan.

12.1.2 Secondary Outcome Measures

Secondary outcome measures listed in Section 2.2 will be analysed with regard to safety and tolerability, improvement from baseline to Day 99 and evaluation of changes in sVAP-1/SSAO as a biomarker of liver disease. Analysis methods for the secondary outcome measures will be detailed further in the Statistical Analysis Plan.

12.2 Analysis of Outcome Measures

This single arm Simon's design (Simon, R., 1989) includes an interim assessment of the accumulating data. The first assessment (stage-1) takes place once 18 patients have been evaluated for the primary outcome (response). If 3 or more responses are observed in stage-1 then the trial will continue into stage-2. Recruitment will not be halted while stage-1 is assessed. If the stage-1 criterion is not met then the trial will cease.

A further 19 patients will be recruited during stage-2. As before, given uncertainty around patient dropout, a set of possible scenarios and the respective stop/go criteria and associated error rates are provided below. The final success criterion chosen maintains power, and in doing so can incur an increased type-1 error rates (note that the stage-1 criterion is fixed since the final sample size is unknown at stage-1).

Table 10: Possible Stop/Go Guidelines and Associated Error Rates

Patients (stage-1)	Responses (stage-1)	Patients (total)	Responses (total)	Type-1 error (α)	Power ($1-\beta$)
18	3	34	8	0.120	0.826
18	3	35	8	0.134	0.846
18	3	36	8	0.149	0.863
**18**	**3**	**37**	**9**	**0.087**	**0.806**
18	3	38	9	0.099	0.827
18	3	39	9	0.111	0.846
18	3	40	9	0.124	0.861
18	3	41	10	0.073	0.809

For peer review only

Not all of the alternative stop/go criteria detailed above are published in the referenced paper. These have been calculated by the trial Biostatistician and independently verified by a CRCTU Biostatistician.

Patients that cannot be evaluated for the primary outcome (e.g. due to withdrawal or lost to follow-up) will be treated as non-responders. Extreme values will be queried by the trials management team, and if present at the final analysis stage may be excluded as part on a sensitivity analysis.

Deviation(s) from the original statistical plan will be documented in version controlled updates to the statistical analysis plan.

12.3 Planned Sub Group Analyses

No subgroup analyses are planned.

12.4 Planned Interim Analysis

Stopping guidelines detailed in section 12.2.

12.5 Planned Final Analyses

Final analyses of the primary outcome will be performed when all patients have been followed to Day 99 and once the database has locked. Stopping guidelines detailed in section 12.2.

12.6 Power Calculations

The Phase II, single arm trial follows a Simon's 2-stage minimax design (Simon, R., 1989) with lower and upper acceptability bounds of 15 and 30% respectively and error rates $\alpha=0.10$ and $\beta=0.20$. Thirty-seven patients are required, however, to account for patient dropout, estimated at approximately 10%, the sample size is extended by a further 4 patients. As such the total target recruitment is 41 patients recruited from 3 UK centres, and we expect to recruit 7 patients per centre per year over a 2-year period.

In this setting the interpretation of α is the probability satisfying stage-1 and observing 9 or more responses in 37 patients overall when the true response rate is 15%; a false positive result (Type I error). Beta (β) is the probability of failing to acknowledge activity when the true response rate is 30% (Type II error). As such the power, 1- β, is the probability of taking an effective treatment forward.

13. TRIAL ORGANISATIONAL STRUCTURE

13.1 Sponsor

The trial sponsor is the University of Birmingham.

13.2 Coordinating Centre

The trial is being conducted under the auspices of the Cancer Research UK Clinical Trials Unit (CRCTU), University of Birmingham according to their local procedures.

13.3 Trial Management Group

The Trial Management Group (TMG) is comprised of the Chief Investigator and other collaborators as detailed elsewhere in this document. The TMG will be responsible for the day-to-day running and management of the trial and will meet by teleconference or in person as required. Members of the TMG include the CI, CRCTU Trial Management Team Leader, Senior Trial Coordinator, Trial

For peer review only - http://bmjopen.bmj.com/site/about/guidelines.xhtml

Coordinator, Lead Trial Statistician, Trial Statistician and a Pharmacy Representative. The TMG will meet or hold a teleconference every 2 months during recruitment, or as required.

13.4 Trial Steering Committee

A Trial Steering Committee (TSC) will provide overall supervision and representation for both the funders and sponsor of the study, whilst providing advice through its independent chair. Membership includes members of the TMG, CRCTU Trial Management Team Leader, and selected Principal Investigators. Other members/observers may be invited upon request. The ultimate decision for the continuation of the study lies with the TSC. The TSC will meet at least once a year or more often if required.

13.5 Data Monitoring Committee

Data analyses will be supplied in confidence to an independent DMC (DMC), which will be asked to give advice on whether the accumulated data from the trial, together with the results from other relevant research, justifies the continuing recruitment of further patients. The DMC will operate in accordance with a trial specific charter based upon the template created by the Damocles Group.

During the run-in period results will be provided to the DMC and discussed via teleconference at a minimum. The DMC will meet after the first 6 patients have completed treatment and the DLT reporting timeline has been completed for these 6 patients. This will be to review the run in phase of the trial. After this phase, the DMC will be scheduled to meet one month prior to the due date of the Annual Safety Report and annually thereafter. Meetings will be required in the second phase of the trial if the DLT rate rises above that specified in the Trial Design section of the protocol. The DMC may halt recruitment if this DLT rate is unacceptable.

During the recruitment phase of the Simon's 2-stage component the DMC will meet at the end of stage 1, after eighteen patients have been enrolled in the trial and yearly thereafter. A meeting will be arranged to coincide with the reporting of Stage-1 stop/go guidelines (see Section 12).

Additional meetings may be called if recruitment is much slower/ faster than anticipated and the DMC may, at their discretion, request to meet more frequently or continue to meet following completion of recruitment. An emergency meeting may also be convened if a safety issue is identified. The DMC will report directly to the Trial Management Group (TMG) who will convey the findings of the DMC to the Trial Steering Committee and funders. The DMC may consider recommending the discontinuation of the trial if the recruitment rate or data quality are unacceptable or if any issues are identified which may compromise patient safety.

The DMC will report directly to the BUTEO Trial Management Group (TMG) who will convey (when applicable) the findings of the DMC to the sponsors, Biotie Therapies, MHRA and the Ethics Committee. The DMC have the right to recommend closure of the trial if any issues are identified which may compromise patient safety.

13.6 Finance

This is a clinician-initiated and clinician-led trial funded by the National Institute for Health Research (NIHR) with receives funds directly from the Department of Health (DoH). Biotie Therapies will be supplying BTT1023, the investigational medicinal product (IMP), free of charge to all individual NHS trusts that will be directly treating patients as part of this clinical trial.

For peer review only - http://bmjopen.bmj.com/site/about/guidelines.xhtml

Payments to individual NHS trusts (Per-patient payment)

Refer to the Clinical Study Site Agreement for details.

14. ETHICAL CONSIDERATIONS

The trial will be performed in accordance with the recommendations guiding physicians in biomedical research involving human subjects, adopted by the 18th World Medical Association General Assembly, Helsinki, Finland, June 1964, amended at the 48th World Medical Association General Assembly, Somerset West, Republic of South Africa, October 1996 (website: http://www.wma.net/en/30publications/10policies/b3/index.html).

The trial will be conducted in accordance with the Research Governance Framework for Health and Social Care, the applicable UK Statutory Instruments, (which include the Medicines for Human Use Clinical Trials 2004 and subsequent amendments and the Data Protection Act 1998) and the Principles of Good Clinical Practice (GCP). This trial will be carried out under a Clinical Trial Authorisation in accordance with the Medicines for Human Use Clinical Trials regulations. The protocol will be submitted to and approved by the main Research Ethics Committee (REC) prior to circulation.

Before any patients are enrolled into the trial, the Principal Investigator at each site is required to obtain local R&D approval. Sites will not be permitted to enrol patients until written confirmation of R&D approval is received by the Trials Office.

It is the responsibility of the Principal Investigator to ensure that all subsequent amendments gain the necessary local approval. This does not affect the individual clinicians' responsibility to take immediate action if thought necessary to protect the health and interest of individual patients.

15. CONFIDENTIALITY AND DATA PROTECTION

Personal data recorded on all documents will be regarded as strictly confidential and will be handled and stored in accordance with the Data Protection Act 1998. With the patient's consent, their full name (on consent form only), initials, date of birth and hospital number, will be collected at trial entry Patients will be identified using only their unique trial number, initials, hospital number and date of birth on the Case Report Form and correspondence between the Trials Office and the participating site. However patients are asked to give permission for the Trials Office to be sent a copy of their signed Informed Consent Form which will not be anonymised. This will be used to perform in-house monitoring of the consent process.

The Investigator must maintain documents not for submission to the Trials Office (e.g. Patient Identification Logs) in strict confidence. In the case of specific issues and/or queries from the regulatory authorities, it will be necessary to have access to the complete trial records, provided that patient confidentiality is protected.

The Trials Office will maintain the confidentiality of all patients' data and will not disclose information by which patients may be identified to any third party other than those directly involved in the treatment of the patient and organisations for which the patient has given explicit consent for data transfer. Representatives of the BUTEO trial team may be required to have access to patient's notes for quality assurance purposes but patients should be reassured that their confidentiality will be respected at all times.

For peer review only

16. INSURANCE AND INDEMNITY

University of Birmingham employees are indemnified by the University insurers for negligent harm caused by the design or co-ordination of the clinical trials they undertake whilst in the University's employment.

In terms of liability at a site, NHS Trust and non-Trust hospitals have a duty to care for patients treated, whether or not the patient is taking part in a clinical trial. Compensation is therefore available via NHS indemnity in the event of clinical negligence having been proven.

The University of Birmingham cannot offer indemnity for non-negligent harm. The University of Birmingham is independent of any pharmaceutical company, and as such it is not covered by the Association of the British Pharmaceutical Industry guideline's for patient compensation.

17. PUBLICATION POLICY

Results of this trial will be submitted for publication in a peer reviewed journal. The manuscript will be prepared by the Trial Management Group (TMG) and authorship will be determined by mutual agreement.

Any secondary publications and presentations prepared by Investigators must be reviewed by the TMG. Manuscripts must be submitted to the TMG in a timely fashion and in advance of being submitted for publication, to allow time for review and resolution of any outstanding issues. Authors must acknowledge that the trial was performed with the support of the University of Birmingham. Intellectual property rights will be addressed in the Clinical Study Site Agreement between Sponsor and site.

For peer review only

For peer review only - http://bmjopen.bmj.com/site/about/guidelines.xhtml

18. REFERENCE LIST

Biotie Therapies Corp., (2008). BTT-1023 anti-VAP-1 monoclonal antibody toxicity study by intravenous (bolus) administration to cynomolgus monkeys for 14 weeks followed by an 8 week recovery period.

Biotie Therapies Corp., (2010). BTT12-CD015: A multiple ascending dose study to assess the safety and pharmacokinetics of repeated intravenous doses of BTT1023 in patients with rheumatoid arthritis - a double-blind randomized placebo-controlled sequential group trial.

Hirschfield, G. M., T. H. Karlsen, K. D. Lindor and D. H. Adams (2013). "Primary sclerosing cholangitis." The Lancet **382**(9904): 1587-1599.

Jalkanen, S. and M. Salmi (2001). "Cell surface monoamine oxidases: enzymes in search of a function." The EMBO Journal **20**(15): 3893-3901.

Kurkijärvi, R., D. H. Adams, R. Leino, T. Möttönen, S. Jalkanen and M. Salmi (1998). "Circulating Form of Human Vascular Adhesion Protein-1 (VAP-1): Increased Serum Levels in Inflammatory Liver Diseases." The Journal of Immunology **161**(3): 1549-1557.

Kurkijärvi, R., G. G. Yegutkin, B. K. Gunson, S. Jalkanen, M. Salmi and D. H. Adams (2000). "Circulating soluble vascular adhesion protein 1 accounts for the increased serum monoamine oxidase activity in chronic liver disease." Gastroenterology **119**(4): 1096-1103.

Lalor, P. F., S. Edwards, G. McNab, M. Salmi, S. Jalkanen and D. H. Adams (2002). "Vascular Adhesion Protein-1 Mediates Adhesion and Transmigration of Lymphocytes on Human Hepatic Endothelial Cells." The Journal of Immunology **169**(2): 983-992.

Liaskou, E., M. Karikoski, G. M. Reynolds, P. F. Lalor, C. J. Weston, N. Pullen, M. Salmi, S. Jalkanen and D. H. Adams (2011). "Regulation of mucosal addressin cell adhesion molecule 1 expression in human and mice by vascular adhesion protein 1 amine oxidase activity." Hepatology **53**(2): 661-672.

Simon, R (1989). "Optimal Two-Stage Designs for Phase II Clinical Trials". Controlled Clinical Trials **10**:1-10

Weston, C. J., E. L. Shepherd, L. C. Claridge, P. Rantakari, S. M. Curbishley, J. W. Tomlinson, S. G. Hubscher, G. M. Reynolds, K. Aalto, Q. M. Anstee, S. Jalkanen, M. Salmi, D. J. Smith, C. P. Day and D. H. Adams (2015). "Vascular adhesion protein-1 promotes liver inflammation and drives hepatic fibrosis." The Journal of Clinical Investigation **125**(2): 501-520.

For peer review only - http://bmjopen.bmj.com/site/about/guidelines.xhtml

APPENDIX 1 – EQUATION FOR ESTIMATED GLOMERULAR FILTRATION RATE

COCKCROFT-GAULT

$$\text{Creatinine clearance (estimated GFR)} = \frac{[(140 - \text{age in years}) \times (\text{wt in kg})] \times 1.23}{\text{serum creatinine in micromol/l}}$$

For women multiply the result of calculation by 0.85.

For peer review only

APPENDIX 2 - COMMON TOXICITY CRITERIA GRADINGS

Toxicities will be recorded according to the Common Terminology Criteria for Adverse Events (CTCAE), version 4.0. The full CTCAE document is available on the National Cancer Institute (NCI) website, the following address was correct when this version of the protocol was approved:

http://ctep.cancer.gov/protocolDevelopment/electronic_applications/ctc.htm

For peer review only

APPENDIX 3 – T-SPOT.TB

Oxford Diagnostics Laboratories

SERVICE DESCRIPTION

Oxford Diagnostic Laboratories (ODL®) offers a national TB testing service to laboratories and clinicians using the T-SPOT®.*TB* test.

Further information on the T-SPOT.*TB* test, including its limitations, is set out in our published documentation.

Specimen Acceptance Criteria

We require 6ml of blood collected in one standard heparin (green-topped) vacutainer tube. Paediatric samples require less blood (please call the laboratory to seek advice, as blood volumes depend on age). Samples must be accompanied with a completed ODL test Request Form and must reach ODL within 32 hrs of venepuncture. Blood samples should be stored at 18-25°C; do not refrigerate samples. Please ensure that you include your Customer Account Number and Quote/Agreement Number on all test requests. Specimens must arrive by 2.00pm Monday to Saturday. ODL is closed on public holidays and over the Christmas and New Year period each year (please call us, or consult our website, to find out the exact dates for this year).

We place no restriction on the numbers of samples that can be sent at one time, however, if sending large numbers of samples we would appreciate advance notification to help us better plan our workflow. We endeavour to provide results two working days after the

sample is received by ODL.

What will Oxford Diagnostic Laboratories do?

1. We will carry out the Service, as described here and in the Terms and Conditions of Service, and following the instructions given on the test Request Form.

2. The service results will be sent by email (and fax or post if requested) to the requesting laboratory within 2 working days of receipt of sample. For incidents, results are reported within three working days.

3. The invoice for the Service will be sent by post within 3 further working days, unless otherwise agreed.

4. ODL will be responsible for disposal of any samples provided.

Results Interpretation and Quality Control

A typical result would be expected to have few or no spots in the Nil Control and _20 spots in the Positive Control. Each spot represents the footprint of an individual cytokine-secreting T cell, and evaluating the number of spots obtained provides a measurement of the abundance of *M. tuberculosis* complex sensitive effector T cells in the peripheral blood.

A Nil Control spot count in excess of 10 spots should be considered as 'Indeterminate'. Refer to the T-SPOT.*TB* Technical Handbook for possible causes (download from www.oxfordimmunotec.com). If this occurs, another sample should be collected from the individual and sent to ODL for testing.

Typically, the cell functionality Positive Control spot count should be _ 20 or show saturation (where spots are too numerous to count).

For peer review only - http://bmjopen.bmj.com/site/about/guidelines.xhtml

A small proportion of patients may have T cells which show only a limited response to PHA. Where the Positive Control spot count is< 20 spots, it should be considered as 'Indeterminate', unless either Panel A or Panel B is 'Positive' as described below, in which case the result is valid.

The T-SPOT.*TB* test results are interpreted by subtracting the spot count in the Nil Control well from the spot count in each of the Panel wells, according to the following algorithm:

- The test result is 'Positive' if (Panel A minus Nil Control) and / or (Panel B minus Nil Control) _ 6 spots.
- The test result is 'Negative' if both (Panel A minus Nil Control) and (Panel B minus Nil Control) _ 5 spots. This includes values less than zero.

Due to potential biological and systematic variations, where the higher of (Panel A minus Nil Control) and (Panel B minus Nil Control) is5, 6 or 7 spots, the result may be considered as Borderline (equivocal). Borderline (equivocal) results, although valid, are less reliable than results where the spot count is further from the cut-off. Retesting of the patient, using a new sample, is therefore recommended.

A 'Positive' result indicates that Tuberculosis infection is likely.

A 'Negative' result indicates that Tuberculosis infection is unlikely.

The results should be used and interpreted only in the context of the overall clinical picture. A negative test result does not exclude the possibility of exposure to or infection with *M. tuberculosis.*

For peer review only

APPENDIX 4 - DEFINITION OF ADVERSE EVENTS

Adverse Event

Any untoward medical occurrence in a patient or clinical trial subject administered a medicinal product and which does not necessarily have a causal relationship with this treatment.

Comment:

An AE can therefore be any unfavourable and unintended sign (including abnormal laboratory findings), symptom or disease temporally associated with the use of an investigational medicinal product, whether or not related to the investigational medicinal product.

Adverse Reaction

All untoward and unintended responses to an IMP related to any dose administered.

Comment:

An AE judged by either the reporting Investigator or Sponsor as having causal relationship to the IMP qualifies as an AR. The expression reasonable causal relationship means to convey in general that there is evidence or argument to suggest a causal relationship.

Serious Adverse Event

Any untoward medical occurrence or effect that at any dose:

- Results in death
- Is life-threatening*
- Requires hospitalisation** or prolongation of existing inpatients' hospitalisation
- Results in persistent or significant disability or incapacity
- Is a congenital anomaly/birth defect
- Or is otherwise considered medically significant by the Investigator***
- Any suspected or definite diagnosis of cytokine release syndrome grade 3 – 5. *In order to confirm the case of cytokine release syndrome, an additional blood sample will need to be taken for cytokine assay (e.g. TNF-α, IFNγ, IL-6). This information is to be provided as part of the SAE form.*

Comments:

The term severe is often used to describe the intensity (severity) of a specific event. This is not the same as serious, which is based on patients/event outcome or action criteria.

* Life threatening in the definition of an SAE refers to an event in which the patient was at risk of death at the time of the event; it does not refer to an event that hypothetically might have caused death if it were more severe.

**Hospitalisation is defined as an unplanned, formal inpatient admission, even if the hospitalisation is a precautionary measure for continued observation. Thus hospitalisation for protocol treatment (e.g. line insertion), elective procedures (unless brought forward because of worsening symptoms) or for social reasons (e.g. respite care) are not regarded as an SAE.

*** Medical judgment should be exercised in deciding whether an AE is serious in other situations. Important AEs that are not immediately life threatening or do not result in death or hospitalisation

For peer review only - http://bmjopen.bmj.com/site/about/guidelines.xhtml

but may jeopardise the subject or may require intervention to prevent one of the other outcomes listed in the definition above, should be considered serious.

Serious Adverse Reaction

An Adverse Reaction which also meets the definition of a Serious Adverse Event.

Suspected Unexpected Serious Adverse Reaction

A SAR that is unexpected i.e. the nature, or severity of the event is not consistent with the applicable product information.

A SUSAR should meet the definition of an AR, UAR and SAR.

Unexpected Adverse Reaction

An AR, the nature or severity of which is not consistent with the applicable product information (e.g. Investigator Brochure for an unapproved IMP or (compendium of) Summary of Product Characteristics (SPC) for a licensed product).

When the outcome of an AR is not consistent with the applicable product information the AR should be considered unexpected.

For peer review only

APPENDIX 5 - WMA DECLARATION OF HELSINKI

WORLD MEDICAL ASSOCIATION DECLARATION OF HELSINKI

Recommendations guiding physicians in biomedical research involving human subjects

Adopted by the 18th World Medical Assembly Helsinki, Finland, June 1964and amended by the29th World Medical Assembly, Tokyo, Japan, October 1975, 35th World Medical Assembly, Venice, Italy, October 1983, 41st World Medical Assembly, Hong Kong, September 1989 and the 48th General Assembly, Somerset West, Republic of South Africa, October 1996

INTRODUCTION

It is the mission of the physician to safeguard the health of the people. His or her knowledge and conscience are dedicated to the fulfilment of this mission.

The Declaration of Geneva of the World Medical Association binds the physician with the words, "The Health of my patient will be my first consideration," and the International Code of Medical Ethics declares that, "A physician shall act only in the patient's interest when providing medical care which might have the effect of weakening the physical and mental condition of the patient."

The purpose of biomedical research involving human subjects must be to improve diagnostic, therapeutic and prophylactic procedures and the understanding of the aetiology and pathogenesis of disease.

In current medical practice most diagnostic, therapeutic or prophylactic procedures involve hazards. This applies especially to biomedical research.

Medical progress is based on research which ultimately must rest in part on experimentation involving human subjects.

In the field of biomedical research a fundamental distinction must be recognized between medical research in which the aim is essentially diagnostic or therapeutic for a patient, and medical research, the essential object of which is purely scientific and without implying direct diagnostic or therapeutic value to the person subjected to the research.

Special caution must be exercised in the conduct of research which may affect the environment, and the welfare of animals used for research must be respected.

Because it is essential that the results of laboratory experiments be applied to human beings to further scientific knowledge and to help suffering humanity, the World Medical Association has prepared the following recommendations as a guide to every physician in biomedical research involving human subjects. They should be kept under review in the future. It must be stressed that the standards as drafted are only a guide to physicians all over the world. Physicians are not relieved from criminal, civil and ethical responsibilities under the laws of their own countries.

I. BASIC PRINCIPLES

1. Biomedical research involving human subjects must conform to generally accepted scientific principles and should be based on adequately performed laboratory and animal experimentation and on a thorough knowledge of the scientific literature.

For peer review only

2. The design and performance of each experimental procedure involving human subjects should be clearly formulated in an experimental protocol which should be transmitted for consideration, comment and guidance to a specially appointed committee independent of the investigator and the sponsor provided that this independent committee is in conformity with the laws and regulations of the country in which the research experiment is performed.

3. Biomedical research involving human subjects should be conducted only by scientifically qualified persons and under the supervision of a clinically competent medical person. The responsibility for the human subject must always rest with a medically qualified person and never rest on the subject of the research, even though the subject has given his or her consent.

4. 4. Biomedical research involving human subjects cannot legitimately be carried out unless the importance of the objective is in proportion to the inherent risk to the subject.

5. Every biomedical research project involving human subjects should be preceded by careful assessment of predictable risks in comparison with foreseeable benefits to the subject or to others. Concern for the interests of the subject must always prevail over the interests of science and society.

6. The right of the research subject to safeguard his or her integrity must always be respected. Every precaution should be taken to respect the privacy of the subject and to minimize the impact of the study on the subject's physical and mental integrity and on the personality of the subject.

7. Physicians should abstain from engaging in research projects involving human subjects unless they are satisfied that the hazards involved are believed to be predictable. Physicians should cease any investigation if the hazards are found to outweigh the potential benefits.

8. In publication of the results of his or her research, the physician is obliged to preserve the accuracy of the results. Reports of experimentation not in accordance with the principles laid down in this Declaration should not be accepted for publication.

9. In any research on human beings, each potential subject must be adequately informed of the aims, methods, anticipated benefits and potential hazards of the study and the discomfort it may entail. He or she should be informed that he or she is at liberty to abstain from participation in the study and that he or she is free to withdraw his or her consent to participation at any time. The physician should then obtain the subject's freely-given informed consent, preferably in writing.

10. When obtaining informed consent for the research project the physician should be particularly cautious if the subject is in a dependent relationship to him or her or may consent under duress. In that case the informed consent should be obtained by a physician who is not engaged in the investigation and who is completely independent of this official relationship.

11. In case of legal incompetence, informed consent should be obtained from the legal guardian in accordance with national legislation. Where physical or mental incapacity makes it impossible to obtain informed consent, or when the subject is a minor, permission from the responsible relative replaces that of the subject in accordance with national legislation. Whenever the minor

For peer review only

child is in fact able to give a consent, the minor's consent must be obtained in addition to the consent of the minor's legal guardian.

12. The research protocol should always contain a statement of the ethical considerations involved and should indicate that the principles enunciated in the present Declaration are complied with.

II. MEDICAL RESEARCH COMBINED WITH PROFESSIONAL CARE

(Clinical Research)

1. In the treatment of the sick person, the physician must be free to use a new diagnostic and therapeutic measure, if in his or her judgement it offers hope of saving life, re-establishing health or alleviating suffering.

2. The potential benefits, hazards and discomfort of a new method should be weighed against the advantages of the best current diagnostic and therapeutic methods.

3. In any medical study, every patient - including those of a control group, if any - should be assured of the best proven diagnostic and therapeutic method. This does not exclude the use of inert placebo in studies where no proven diagnostic or therapeutic method exists.

4. The refusal of the patient to participate in a study must never interfere with the physician-patient relationship.

5. If the physician considers it essential not to obtain informed consent, the specific reasons for this proposal should be stated in the experimental protocol for transmission to the independent committee (I, 2).

6. The physician can combine medical research with professional care, the objective being the acquisition of new medical knowledge, only to the extent that medical research is justified by its potential diagnostic or therapeutic value for the patient.

III. NON-THERAPEUTIC BIOMEDICAL RESEARCH INVOLVING HUMAN SUBJECTS (Non-Clinical Biomedical Research)

1. In the purely scientific application of medical research carried out on a human being, it is the duty of the physician to remain the protector of the life and health of that person on whom biomedical research is being carried out.

2. The subject should be volunteers - either healthy persons or patients for whom the experimental design is not related to the patient's illness.

3. The investigator or the investigating team should discontinue the research if in his/her or their judgement it may, if continued, be harmful to the individual.

4. In research on man, the interest of science and society should never take precedence over considerations related to the wellbeing of the subject.

For peer review only

BMJ Open

Investigating the safety and activity of the use of BTT1023 (Timolumab), in the treatment of patients with primary sclerosing cholangitis (BUTEO): A single arm, two-stage, open-label, multi-centre, phase II clinical trial protocol.

Journal:	*BMJ Open*
Manuscript ID	bmjopen-2016-015081.R1
Article Type:	Protocol
Date Submitted by the Author:	29-Mar-2017

Complete List of Authors:	Arndtz, Katherine; University of Birmingham, Institute of Immunology & Immunotherapy<br>Corrigan, Margaret; Centre for Liver Research, University of Birmingham and NIHR Birmingham Liver Biomedical Research Unit<br>Rowe, Anna; NIHR Birmingham Liver Biomedical Research Unit Clinical Trials Group (D3B team), CRUK Clinical Trials Unit, University of Birmingham, Birmingham, UK<br>Kirkham, Amanda; Statistics, CRUK Clinical Trials Unit, University of Birmingham, Birmingham, UK<br>Barton, Darren; University of Birmingham, NIHR Liver BRU Clinical trials group (EDD), CRUK clinical trials unit<br>Fox, Richard; Cancer Research Clinical Trials Unit, Biostatistics<br>Llewellyn, Laura; Early Drug Development Team, CRUK Clinical Trials Unit, University of Birmingham, Birmingham, UK<br>Athwal, Amrita; NIHR Birmingham Liver Biomedical Research Unit Clinical Trials Group (D3B team), CRUK Clinical Trials Unit, University of Birmingham, Birmingham, UK<br>Wilkhu, Manpreet; NIHR Birmingham Liver Biomedical Research Unit Clinical Trials Group (D3B team), CRUK Clinical Trials Unit, University of Birmingham, Birmingham, UK<br>Chen, Yung-Yi; NIHR Liver Biomedical Research Unit, Centre for Liver Research, University of Birmingham, Birmingham, UK<br>Weston, Chris; NIHR Liver Biomedical Research Unit, Centre for Liver Research, University of Birmingham, Birmingham, UK<br>Desai, Amisha<br>Adams, David; NIHR Liver Biomedical Research Unit, Centre for Liver Research, University of Birmingham, Birmingham, UK<br>Hirschfield, Gideon; University of Birmingham, School of Immunity and Infection

**Primary Subject Heading**:	Gastroenterology and hepatology
Secondary Subject Heading:	Immunology (including allergy)
Keywords:	Immunology < BASIC SCIENCES, Hepatobiliary disease < GASTROENTEROLOGY, IMMUNOLOGY, Hepatology < INTERNAL MEDICINE, Clinical trials < THERAPEUTICS

SCHOLARONE™
Manuscripts

For peer review only

1

Investigating the safety and activity of the use of BTT1023 (Timolumab), in the treatment of patients with primary sclerosing cholangitis (BUTEO): A single arm, two-stage, open-label, multi-centre, phase II clinical trial protocol.

Katherine Arndtz[1], Margaret Corrigan[1], Anna Rowe[2], Amanda Kirkham[3], Darren Barton[2], Richard Fox[3], Laura Llewellyn[4], Amrita Athwal[2], Manpreet Wilkhu[2], Yung Yi Chen[1], Chris Weston[1], Amisha Desai[5], David H Adams[1], Gideon M Hirschfield[1], on behalf of the BUTEO trial team.

[1]NIHR Liver Biomedical Research Unit, Centre for Liver Research, University of Birmingham, Birmingham, and Birmingham Health Partners, Institute of Translational Medicine, University Hospitals Birmingham, Birmingham, UK

[2]NIHR Birmingham Liver Biomedical Research Unit Clinical Trials Group (D^3B team), CRUK Clinical Trials Unit, University of Birmingham, Birmingham, UK

[3]Statistics, CRUK Clinical Trials Unit, University of Birmingham, Birmingham, UK

[4]Early Drug Development Team, CRUK Clinical Trials Unit, University of Birmingham, Birmingham, UK

[5]Pharmacy, University Hospitals Birmingham NHS Foundation Trust, Queen Elizabeth Hospital Birmingham

Corresponding author: Professor Gideon Hirschfield, Centre for Liver Research, Institute of Immunology & Immunotherapy, University of Birmingham, Edgbaston, Birmingham, UK, B15 2TT

Email: g.hirschfield@bham.ac.uk

For peer review only

Keywords (5 max): Autoimmune liver disease; Early phase clinical trials; Anti-fibrotic; Serum amine oxidase

Word count excluding abstract/references: 4952

For peer review only

Abstract

Introduction:

Primary sclerosing cholangitis (PSC) is a progressive inflammatory liver disease characterised by relentless liver fibrosis and a high unmet need for new therapies. Preventing fibrosis represents an important area of interest in the development of vital new drugs. Vascular adhesion protein-1 (VAP-1) drives inflammation in liver disease, and provision of an antibody against VAP-1 blunts fibrosis in murine models of liver injury.

Methods & Analysis:

BUTEO is a single arm, two-stage, open-label, multi-centre, phase II clinical trial. Up to 59 patients will receive treatment with anti-VAP monoclonal antibody, BTT1023, over a 78 day treatment period. Adults with PSC and a serum alkaline phosphatase (ALP) of at least 1.5 times the upper limit of normal will be included. Our primary outcome measure is a reduction in ALP by >25% from baseline to Day 99. Secondary outcome measures include safety and tolerability, changes pre/post therapy in circulating serum VAP-1 as well as imaging findings. The first patient participant was recruited on 08/09/2015.

Ethics & Dissemination:

This protocol has been approved by the Research Ethics Committee (REC, reference 14/EM/1272). The first REC approval date was 06/01/2015 with three subsequent approved amendments. This article refers to protocol version 3.0, dated 16/03/2016. Results will be disseminated via peer reviewed publication and presentation at international conferences.

Trial registration:

The trial is registered with the European Medicines agency (EudraCT: 2014-002393-37), the NIHR (PortfolioID:18051) and ISRCTN: 11233255. The clinicaltrials.gov identifier is NCT02239211.

For peer review only

4

Strengths:-

- Unique, tailor-made clinical trial design incorporating a dose confirmatory and safety stage (based on the traditional 3+3 design), then followed by a Phase II Simon's two-stage design.
- Brings the translation of laboratory research into a proof of activity clinical trial.
- An early phase experimental medicine study of a novel first-in-class drug, in a chronic disease cohort with a large unmet need for new therapies.
- Aims to address not just the need for new therapies but also the need for reliable clinical trial endpoints as well as biomarkers for staging and predicting clinical outcomes.

Limitations:-

- Small cohort due to PSC being a rare orphan disease as well as unpredictability of the disease making stability for clinical trial inclusion difficult.
- Short duration of the treatment period in which goal is to demonstrate collective markers of efficacy to justify longer and placebo-controlled trials.
- Limited evidence base for the primary endpoint of a reduction in ALP in the context of anti-fibrotic agents, however accepting there is no alternative surrogate currently available.
- Translational study from mice into human subject and the unknown differences this may entail.

For peer review only

INTRODUCTION

End-stage liver disease, regardless of aetiology, is characterised by progressive hepatic fibrosis culminating in liver cirrhosis and accompanying increased risks of liver cancer, liver failure, portal hypertension and death. Preventing progressive liver fibrosis represents an important area of interest in the development of new drugs suitable for all patients with liver disease. Primary sclerosing cholangitis (PSC) is a prime example of a progressive inflammatory liver disease which is characterised by persistent liver fibrosis and a high unmet need for new therapies. PSC has a population incidence of 1.3/100,000 annually, with a prevalence of 16.2 per 100,000[1,2,3]. It affects both men and women, with a median age of 41[4] and in 80% of cases is associated with Inflammatory Bowel Disease (IBD)[5]. More than 50% of patients require liver transplantation within 10-15 years of symptomatic presentation[6,7], reflecting the failure of medical therapies to have any impact on the clinical outcome: in the UK for example, PSC is now the leading autoimmune liver disease indication for transplant, despite being the rarest of the autoimmune liver diseases. One barrier to the development of efficacious new medical therapies is the lack of clinically relevant end-points and there is an urgent need to develop appropriate non-invasive surrogate endpoints to improve clinical trial design[8].

Vascular Adhesion Protein-1 (VAP-1)

Vascular adhesion protein-1 (VAP-1) is a 170-kDa homodimeric type 2 transmembrane sialoglycoprotein with a short cytoplasmic tail of no known signal sequence, a single transmembrane segment, and a large extracellular domain. VAP-1 is constitutively expressed on human hepatic endothelium and supports lymphocyte adhesion and

For peer review only

transendothelial migration. Cloning of VAP-1 revealed it to be a copper-dependent semicarbazide-sensitive amine oxidase (SSAO) which catalyzes the oxidative deamination of exogenous and endogenous primary amines resulting in the generation of aldehyde, ammonia, and H2O2. These products activate NFκB-dependent chemokine secretion and adhesion molecule expression in liver endothelium and may initiate and propagate oxidative stress following the conversion of H2O2 to hydroxyl free radicals. A soluble form of VAP-1 (sVAP-1) accounts for nearly all of the circulating amine oxidase activity in humans.[9]

The progression of PSC to scarring, cirrhosis and hepatobiliary cancer is driven by a chronic inflammatory response and immune cell mediated destruction of bile ducts[10]. Our research implicates VAP-1 in the inflammation that drives fibrogenesis in liver disease[11]. VAP-1 also acts as an adhesion receptor to support leucocyte recruitment in liver inflammation, a function that is critical in animal models in the formation of fibrosis[9,12]. Thus, inhibition of VAP-1 is expected to impact both inflammation and fibrosis, indeed treatment with an antibody against VAP-1 prevents fibrosis in murine models of liver injury[9]. Data also shows particularly high levels of circulating serum VAP-1 (sVAP-1) in patients with PSC as well as a strong correlation between sVAP-1/semicarbazide-sensitive amine oxidase (SSAO) activity in serum and histological fibrosis scores in patients with fatty liver disease[9]. Based on the strong up-regulation of hepatic VAP-1 reported in PSC patients[9], we hypothesise that levels of sVAP-1/SSAO will correlate with the severity of fibrosis in PSC and predict patients at risk of progressive disease.

These observations underpin our proposal that VAP-1 has an important role in the progression of liver fibrosis. We now plan to test the hypothesis that inhibiting VAP-1 with a

For peer review only

neutralising antibody (BTT1023) will reverse or delay fibrogenesis in patients with PSC. Additionally, reliable biomarkers that correlate with fibrosis stage and progression of liver disease are in demand in order to predict outcome and to stage disease, without the need for invasive liver biopsy. This research will allow us to translate laboratory research into a proof of activity clinical trial that will elucidate the role of VAP-1 in liver fibrosis and its potential as a therapeutic target and biomarker.

BTT1023 (now known as Timolumab)

BTT1023 is a fully human, monoclonal, anti-VAP-1 antibody which blocks the adhesion function of VAP-1, thereby diminishing leukocyte entry into sites of tissue inflammation. Going forward BTT1023 will be known as Timolumab, however the original numeric name is used in this manuscript in order to remain in keeping with the trial protocol. In vivo, blocking VAP-1 function with an anti-mouse anti-VAP-1 antibody significantly alleviates inflammation in mouse models of arthritis and liver fibrosis[9,13,]. BTT1023 appears to be safe and well tolerated in humans: BTT1023 has been given in doses up to 8 mg/kg in patients with rheumatoid arthritis and psoriasis after oral premedication (cetirizine & ibuprofen) and also appears safe and well-tolerated in repeated intravenous dosing[14]. No cytokine release syndrome was seen. This dose therefore appears reasonable as a starting point and is considered to provide appropriate bridging into existing clinical data.

In the well tolerated arthritis study, patients received 5 doses at fortnightly intervals with potentially efficacious trough levels being achieved by Day 42, after 3 doses. The psoriasis study used a more rapid loading of 3 doses at days 1, 8 and 22, which was also well

For peer review only

tolerated. Therefore, we have decided to deploy the accelerated dosing scheme (i.e. dosing on days 1, 8, and biweekly thereafter) in the current study. [14]

BTT1023 is produced in a Chinese hamster ovary cell culture and purified with appropriate methods including specific viral inactivation and removal procedures. BTT1023 is contained in single-use 100 mg/10 mL glass vials and requires dilution with BTT1023 IV Infusion Diluent (0.9% sodium chloride and 0.02% polysorbate 80 in water for injection) prior to administration by intravenous infusion. The amount of diluent to be added to the BTT1023 concentrate is calculated to consistently provide a total diluted drug product infusion volume of 50 mL. BTT1023 and its diluent are stored at 2-8°C and the maximum shelf life for the diluted infusion solution is 24 hrs when stored at this temperature.

Objectives

The trial primary objective is to determine the activity of the anti-VAP-1 antibody BTT1023 in patients with PSC as measured by a decrease in alkaline phosphatase levels (ALP) (primary endpoint) with secondary endpoints to include various measures of liver injury and fibrosis and evaluation of the safety, effective dose, and tolerability of BTT1023 in patients with PSC. Our secondary objectives include; determining the mechanisms of action of BTT1023 through in vitro assessment of sVAP-1 concentration, SSAO enzyme activity and immune cell function; evaluating the potential of a novel MRI based assessment of liver fibrosis and biliary strictures for assessing therapeutic response in PSC; and assessment of the use of sVAP-1/SSAO as a biomarker to monitor disease progression in PSC.

For peer review only

METHODS

Study design overview

BUTEO is a two-stage single-arm, open-label, multi-centre hybrid trial of treatment with monoclonal anti-VAP-1 antibody, BTT1023 in adult patients with PSC. The sample size will be a maximum of 59 patients, who will each have up to 7 intravenous infusions of BTT1023 over a 78 day treatment period. All patients will be followed up until Day 120 (42 days after the last administration of treatment). At specified time points during each visit, serum will be taken for circulating levels of BTT1023 as well as for anti-drug antibodies, VAP-1 activity, and additional exploratory research samples. This includes pre, during and post-dose, with some patients also attending 24 hours later for further blood testing.

The trial is comprised of two components. The run-in component of the trial incorporates a conventional 3+3 cohort design to confirm the therapeutic dose, with decisions regarding continuation based on toxicity and pharmacokinetic (PK) data, Figure 1. The trial begins with the recruitment of 6 patients all receiving the starting dose of 8 mg/kg. Recruitment will be paused while awaiting the results of trough blood serum levels of circulating BTT1023 at Visit 7 (Day 50) from all 6 patients and until the Dose Limiting Toxicity (DLT) reporting period is completed for each patient (Visit 10 [Day 99]).

If results from the first cohorts show an acceptable DLT rate (see later) and trough levels of BTT1023 meet the stipulated success criteria, the trial will continue into the expansion component of the trial. Acceptable trough levels have been set a trough concentration of 3 µg/mL free circulating BTT1023 at 8 weeks from first infusion, which is approximately 100 fold the dissociation constant (Kd) of BTT1023 from VAP-1 and will result in target

For peer review only

occupancy of approximately 90%. In the event that DLT rate is acceptable but the PK levels do not meet the success criteria then the trial moves into a conventional 3+3 cohort design, using escalating doses of BTT1023. In this event, the original 6 patient cohort will no longer be evaluated, but a new cohort of 3 patients will be recruited to receive the newly identified test dose of 12 mg/kg. If there are no DLTs at Visit 10 [Day 99], an additional cohort of 3 patients will be recruited at the new test dose. If the DLT rate remains acceptable but the PK levels still do not meet the success criteria, a further 3+3 patients will be recruited at the highest dose of 16mg/kg. If this is found not to result in sufficient blood levels of BTT1023 then the trial will be stopped. If the PK values are found to be too high (such as resulting in trough levels consistently exceeding 100 µg/mL) then there is potential to de-escalate the dose in agreement with the DMC and after regulatory approvals. The trial will be stopped at any stage where patient safety is compromised. Individual patients will only receive one dose level. Once a confirmed dose has been established, the trial will expand until a total of 37 patients have received treatment with the confirmed dose, including those patients who have previously received this dose during the confirmatory period. Those patients not receiving the confirmed dose will not be included in the final evaluation.

Patient selection

Patients with particularly elevated ALP levels are predicted to be more at risk of progressive disease; thus these patients will be selected. Currently four UK academic hospital centres are involved (based in Birmingham, Nottingham, Oxford and Newcastle) with further centres potentially coming on board shortly. Informed consent will be obtained by appropriately trained members of the research team at each site.

For peer review only

The main inclusion criteria are patients aged 18 to 75 with a clinical diagnosis of PSC, as evidenced by chronic cholestasis of more than six months duration with either an MRI or liver biopsy consistent with PSC and in the absence of a documented alternative aetiology. Patients must have an ALP of at least 1.5 times the upper limit of normal. Those with concomitant inflammatory bowel disease (IBD) must have evidence to show clinically and colonoscopically stable disease within the past 12 months, without findings of high grade dysplasia and without the need for biological therapies. In those on treatment with ursodeoxycholic acid (UDCA), therapy must be stable for at least 8 weeks prior to screening and at a dose not greater than 20 mg/kg/day. In those not on treatment with UDCA at the time of screening, a minimum of 8 weeks since the last dose of UDCA should be recorded. As an IgG molecule, BTT1023 is not anticipated to interfere with chromosomal material however teratogenicity has not been studied and so rigorous steps will be taken to exclude pregnancy pre-treatment and with regards to need for effective contraception throughout the trial and for 99 days after. Some of the main exclusion criteria can be seen in Box 1.

TREATMENT

A single arm, rather than placebo controlled design, was chosen to allow efficient enrolment of patients into the study due to its intensive nature, in which a significant chance of being allocated into the placebo group may act as a substantial barrier for enrolment. The proposed primary endpoint (ALP) is a biochemical measurement and thus not open to subjective bias as a clinical assessment would be, rendering the necessity of a concurrent control group less vital.

For peer review only

TRIAL SCHEMA

Given the unpredictable nature of PSC and natural variation of ALP levels, we have built into the trial a two stage screening process over 4-7 weeks, whereby the ALP levels must not vary by more than 25% in order to continue to enrolment. During screening, patients have routine blood screens and other non-invasive markers of liver fibrosis including Mayo PSC Risk Score, MELD, Enhanced Liver Fibrosis (ELF), Fibroscan and MRCP with additional LiverMultiscan® imaging. These will then be repeated during treatment and in the follow-up period to assess for any change.

During all 7 treatment visits, patients will receive pre-medication with cetirizine 10 mg + ibuprofen 400 mg orally (in the absence of any contra-indications) plus intravenous hydrocortisone 100 mg, 1-2 hrs pre infusion (the latter for the first 3 doses only). The first infusion will be given over 2 hours, with a 4 hour monitoring period post-infusion. Provided no adverse reactions are seen, the infusion time will drop to 1 hour with initially a 3 hour observation period (for the second dose) and then down to 2 hours monitoring post-infusion (for all subsequent doses). Safety investigations completed pre and post-infusion include haematology/biochemistry sampling, along with ECG, clinical assessment and physical examination. The full trial schema can be seen as Figure 2.

A 0.5-1.0 mL aliquot of the BTT1023 infusion solution will be taken at the end of every infusion and refrigerated. These samples may be used for analysis of BTT1023 concentration if anomalies in PK data are observed that could be due to errors in IMP preparation.

For peer review only

Data analysis plan:

Power calculations & Sample size justification

The dose confirmatory stage requires a maximum of 18 patients. The sample size has been based on the classic 3+3 design, investigating three fixed dose increments (8, 12 and 16 mg/kg), with no dose skipping.

The expansion phase follows a Simon's 2-stage minimax design with lower and upper acceptability bounds of 15 and 30% respectively and error rates α=0.10 and β=0.20. Thirty-seven patients are required in this stage of the trial, however, to account for patient dropout, estimated at approximately 10%, the sample size is extended by a further 4 patients. As such the target recruitment is up to 41 patients for the expansion phase, and up to 59 participants in total. An interim assessment will examine the primary outcome once 18 evaluable patients have been recruited at the confirmed dose. If 3 or more responses are observed in stage 1, then the trial will continue into stage 2. Recruitment will not be halted while stage 1 is assessed. If the stage 1 criterion is not met then the trial will cease. However if the criterion is met, then further patient recruitment continues until 37 evaluable patients are recruited. If overall there are 9 or more responses from 37 evaluable patients then we conclude that the treatment warrants further investigation.

In this setting the interpretation of alpha (α) is the probability satisfying stage-1 and observing 9 or more responses in 37 patients overall when the true response rate is 15%; a false positive result (Type I error). Beta (β) is the probability of failing to acknowledge activity when the true response rate is 30% (Type II error). As such the power, 1- β, is the probability of taking an effective treatment forward.

For peer review only

OUTCOME MEASURES & ANALYSIS

This is an early phase trial of BTT1023 in immune mediated liver disease, with the rationale to identify biochemical efficacy (reduction in ALP) and safety, in an orphan disease that presently lacks any other effective medical therapy. The trial design therefore focuses on identifying early biochemical efficacy signals to justify larger scale, randomised controlled trials of a longer duration.

Our primary outcome measure is patient response to treatment at Day 99, and is a reduction in serum ALP by 25% or more from baseline to Day 99. Our data on stability of ALP in PSC suggests that such responses occur very seldom during the natural course of the disease, and we can therefore reliably assess changes from baseline and response rates for this proof of concept study to evaluate the therapeutic potential of BTT1023. In addition we are excluding patients in whom the levels change significantly naturally by >25% in the 5-7 week screening period.

Secondary outcome measures include safety and tolerability (via treatment compliance, patient withdrawal, frequency of SAE/AEs, change in quality of life questionnaires: EQ-5D, Fatigue Severity Scale, Pruritus Visual Analogue Score, inflammatory bowel disease diaries), and of BTT1023 efficacy (via tests of liver fibrosis including ELF, Fibroscan and liver biochemistry). Additionally liver MRI is an emerging method for monitoring liver disease and its treatment. We will evaluate changes in MRI imaging pre and post therapy using the LiverMultiscan® protocol (or equivalent methodology, at sites where this is possible). Finally we will evaluate changes in sVAP-1/SSAO as a biomarker of liver disease activity across the study period.

For peer review only

INTERIM ANALYSIS

An interim analysis will be completed once 18 patients have been evaluated for the primary outcome (ALP response). If 3 or more responses are observed then the trial will continue. If this is not met then the trial will cease. If adequate response is seen, a further 19 patients will be recruited in order to obtain the required sample size of 37 patients – allowing for 10% patient drop out during trial duration, this number could reach a total of 41 patients recruited. The final success criterion chosen maintains power, and in doing so can incur an increased type-1 error rates (note that the stage-1 criterion is fixed since the final sample size is unknown at stage-1). These have been calculated by the trial Biostatistician and independently verified by a CRCTU Biostatistician.

Patients that cannot be evaluated for the primary outcome (e.g. due to withdrawal or lost to follow-up) will be treated as non-responders.

Final analyses

Final analyses of the primary outcome will be performed when all patients have been followed to Day 120 and once the database has been locked. If overall there are nine or more responses from 37 evaluable patients then we conclude that the treatment warrants further investigation. Only patients treated at the confirmed dose will contribute to the total patient requirement.

For peer review only

CONDUCT OF THE TRIAL

This is a clinician-initiated and clinician-led trial funded by the National Institute for Health Research (NIHR) which receives funds directly from the Department of Health (DoH). Biotie Therapies will be supplying BTT1023 free of charge to all individual NHS trusts that will be directly treating patients as part of this clinical trial. There are payments to individual NHS trusts on a per-patient basis, to cover trial running costs. The University of Birmingham are acting as trial sponsor and as such remains responsible for the study conduct.

ADVERSE EVENT REPORTING

Dose Limiting Toxicity

Dose Limiting Toxicity (DLT) is defined as an Adverse Event (AE) that meets the criteria of grade 3 cytokine release syndrome or grade 4/5 for any criteria, as defined in the Common Terminology Criteria for Adverse Events (CTCAE V4.0). Although previous studies have shown no DLTs with BTT1023, toxicity monitoring will be ongoing throughout the trial and any concerns with be reported to the Trials office within 24 hours of the investigator becoming aware of the event. The DLT reporting period is from Visit 3 (first infusion) to Visit 10 (21 days after last infusion). An acceptable DLT rate has been established for the trial as a maximum of 1 incidence in 6 patients (~17%). If the DLT rate rises to 2 or more, at any stage during the DLT reporting period, (Visit 3 [Day 1] to Visit 10 [Day 99]) the trial will be halted in consultation with the DMC.

AEs/SAEs

The collection and reporting of Adverse Events (AEs) will be in accordance with the Medicines for Human Use Clinical Trials Regulations 2004 and its subsequent amendments.

For peer review only - http://bmjopen.bmj.com/site/about/guidelines.xhtml

The CTCAE version 4.0 criteria will be used to grade each AE. Any pre-existing conditions will be reported in the medical history, and will not be reported as an AE unless the condition worsens by at least one CTC grade during the trial. The reporting period for AEs will commence from the date of consent (Visit 1) and will continue until the final follow-up visit (Visit 11: Day 120), or alternatively up to 45 days post last infusion if the patient withdraws from the study prior to completion of all 7 study drug infusions. All trial patients will continue to receive standard concomitant clinical care throughout the study.

The Sponsor, appropriate regulatory authority (e.g. Medicines and Healthcare Products Regulatory Agency (MHRA)) and the Research Ethics Committee (REC) will be informed of all SAEs as required by current regulations. An SAE judging to have a reasonable causal relationship to the drug will be recorded as a Serious Adverse Reaction (SAR) or Suspected Unexpected Serious Adverse Reaction (SUSAR), as appropriate and will be reported to the MHRA and REC within 7 days. The independent Data Monitoring Committee (DMC) will also review all SAEs.

In the event that a patient or their partner becomes pregnant during the SAE reporting period this will be recorded and reported and followed up, subject to required patient/partner approvals.

MONITORING

Independent onsite monitoring will be carried out as required following an initial site-specific risk assessment. Additional on-site monitoring visits will be triggered for example by poor CRF return, poor data quality, low SAE reporting rates, excessive number of patient

For peer review only

withdrawals or deviations. Any major problems identified during monitoring may be reported to the Trial Management Group and the Trial Steering Committee and the relevant regulatory bodies.

DISCUSSION

PSC is a prime example of a progressive inflammatory liver disease characterised by relentless liver fibrosis. There is a high unmet need for new therapies as no currently licensed therapy has been shown to alter the natural course of the disease. The progression of PSC to scarring, cirrhosis and hepatobiliary cancer is driven by a chronic inflammatory response and immune cell mediated destruction of bile ducts. Our research suggests that VAP-1 is heavily implicated as a key driver for fibrogenesis and as such, provides a target for the possible slowing or even reversal of the liver damage seen in PSC.

The unpredictability of PSC along with its designation as a rare orphan disease pose particular challenges in trial design. ALP fluctuates during the natural course of the disease which limits its usefulness as a primary endpoint, however it is commonly used as a standard marker of PSC disease activity, in the absence of a viable alternative. Thus, reliable biomarkers that correlate with fibrosis stage and progression of liver disease are in demand in order to predict outcome and to stage disease without the need for invasive liver biopsy, and this trial will aid in investigating the role of VAP-1 in liver fibrosis and its potential as a therapeutic target and biomarker.

This unique trial design, incorporating a dose confirmatory and safety stage (based on the traditional 3+3 design), then followed by a Phase II Simon's two-stage design is aimed to

For peer review only

determine not only a safe and well tolerated dose of BTT1023, but also the efficacy of this treatment in a new disease group.

Screening for this study commenced on 01/02/2015 with the first patient registered on 10/09/2015. Recruitment is ongoing.

Ethics & Dissemination:

The trial is being performed in accordance with the recommendations guiding physicians in biomedical research involving human subjects, adopted by the 48th World Medical Association General Assembly as well as the Research Governance Framework for Health and Social Care, the applicable UK Statutory Instruments, (which include the Medicines for Human Use Clinical Trials 2004 and subsequent amendments and the Data Protection Act 1998) and Guidelines for Good Clinical Practice. The protocol has been approved by the Research Ethics Committee (REC) with the reference of; 14/EM/1272. The first REC approval date was 06/01/2015 with subsequent amendments on 18/3/2015 (non-substantial), 27/11/2015 (substantial) and 16/3/2016 (substantial) also now approved. All active sites have obtained local Research & Development department approval and are up-to-date with the latest protocol amendment.

Standard regulations for data handling and patient confidentiality are being followed, in accordance with the 1998 UK Data Protection Act. During the trial, patients will be identified using only their unique trial number, initials and date of birth on the Case Report Form (CRF) and in correspondence between the Trials Office and the participating site. Data quality will be maintained according to recognised guidance and will be consistent to the source data.

For peer review only

All essential trial documentation and source records will be securely retained for at least 15 years after the end of the trial or following the processing of all biological material collected for research, whichever is the latter. Results will be disseminated via peer reviewed publication and presentation at international conferences; additional summaries will be provided to patients and patient support groups.

For peer review only

Acknowledgements:

This project is funded by the Efficacy and Mechanism Evaluation (EME) Programme*, an MRC and NIHR partnership. The authors would like to thank the NIHR, the NIHR Liver BRU in Birmingham, Biotie Therapies Corp, the Queen Elizabeth Hospital Liver PPI group and the patients involved. *The EME Programme is funded by the MRC and NIHR, with contributions from the CSO in Scotland and NISCHR in Wales and the HSC R&D Division, Public Health Agency in Northern Ireland.

Authors' contributions: All authors have read and approved the final manuscript. KA is lead sub-investigator at the Birmingham BUTEO site and wrote this manuscript in conjunction with GMH. CW, MC, AR, MW, RF, AK, DHA, DB and GMH were involved in the protocol development. RF & AK also contribute statistical analysis. DHA was responsible for the original concept.

Funding & NIHR disclaimer: This work is supported by the NIHR Efficacy and Mechanism Evaluation (EME) Programme, grant number 12/165/31. The full title is: "Targeting vascular adhesion protein-1 (VAP-1) for the treatment of liver fibrosis: a study of efficacy and mechanisms in patients with primary sclerosing cholangitis (PSC)". This article represents independent research funded by the National Institute for Health Research (NIHR) Birmingham Liver Biomedical Research Unit (BRU). The views expressed in this publication are those of the author(s) and not necessarily those of the MRC, NHS, NIHR or the Department of Health.

For peer review only

Competing interests: CW and DHA report grants from NIHR MRC, during the conduct of the study; grants and non-financial support from Pharmaxis Inc, Australia, outside the submitted work; and have a patent "The use of VAP-1 inhibitors for treating fibrotic conditions (WO 2011029996 A1)" issued. Other authors have no competing interests declared.

For peer review only

**Box 1: Main Exclusion criteria (not a complete list)**
Subjects must **NOT** meet any of the following:-
- AST and ALT >10 x ULN or bilirubin >3xULN or INR >1.3 in the absence of anti-coagulants.<br>- Serum creatinine >130 µmol/L or platelet count <50 x 10^9/L.<br>- Any evidence of hepatic decompensation past or present, including ascites, hepatic encephalopathy or variceal bleeding.<br>- Recent cholangitis within last 90 days or ongoing need for prophylactic antibiotics.<br>- Pregnancy or breast feeding.<br>- Flare in colitis activity within last 90 days requiring intensification of therapy beyond baseline maintenance treatment; use of oral prednisolone >10 mg/day, biologics (i.e. monoclonal antibodies) and or hospitalisation for colitis within 90 days. Prior use of biologics is not a contraindication to screening.<br>- Diagnosed cholangiocarcinoma or high clinical suspicion of cholangiocarcinoma.<br>- Concurrent malignancies or invasive cancers diagnosed within past 3 years except for adequately treated basal cell and squamous cell carcinoma of the skin and in situ carcinoma of the uterine cervix.<br>- Presence of a percutaneous drain, bile duct stent or prior organ transplantation.<br>- Participation in an investigational trial of a drug or device within 60 days of screening or 5 half-lives of the last dose of investigational drug.<br>- Positive screening test for tuberculosis (TB) (including T-SPOT.TB TB test), unless respiratory review confirms false positive test results.<br>- Receipt of live vaccination within 6 weeks prior to baseline visit.

For peer review only

Figure 1.

Flow diagram showing the trial decision guidelines for the run-in period of the trial design incorporating a conventional 3+3 cohort design if dose escalation is required

For peer review only

25

Figure 2. BUTEO Trial Schema

For peer review only

26

References:

[1] Lindkvist B, Benito de Valle M, Gullberg B, Bjornsson E. Incidence and prevalence of primary sclerosing cholangitis in a defined adult population in Sweden. Hepatology. 2010 Aug;52(2):571–7.

[2] Bambha K, Kim WR, Talwalkar J, Torgerson H et al. Incidence, clinical spectrum, and outcomes of primary sclerosing cholangitis in a United States community. Gastroenterology. 2003 Nov;125(5):1364–9.

[3] Molodecky NA, Kareemi H, Parab R, Barkema HW et al. Incidence of primary sclerosing cholangitis: a systematic review and meta-analysis. Hepatology. 2011 May;53(5):1590–9. doi: 10.1002/hep.24247.

[4]Bergquist A, Said K, Broomé U. Changes over a 20-year period in the clinical presentation of primary sclerosing cholangitis in Sweden. Scand J Gastroenterol. 2007 Jan;42(1):88-93.

[5] Loftus EV, Sandborn WJ, Lindor KD, LaRusso NF. Interactions between chronic liver disease and inflammatory bowel disease. Inflamm Bowel Dis 1997;3:288–302.

[6] Boonstra K, Weersma RK, van Erpecum KJ, Rauws EA et al. Population-based epidemiology, malignancy risk, and outcome of primary sclerosing cholangitis. Hepatology 2013 Dec;58(6):2045-2055.

[7] Broome U, Olsson R, Loof L, Bodemar G et al. Natural history and prognostic factors in 305 Swedish patients with primary sclerosing cholangitis. Gut 1996 Apr;38(4):610-615.

[8] Ponsioen CY, Chapman RW, Chazouillères O, Hirschfield GM et al. Surrogate endpoints for clinical trials in primary sclerosing cholangitis: Review and results from an International PSC Study Group consensus process. Hepatology. 2016 Apr;63(4):1357-67.

For peer review only

27

[9] Weston CJ, Shepherd EK, Claridge LC, Rantakari P et al. Vascular adhesion protein-1 promotes liver inflammation and drives hepatic fibrosis. J Clin Invest. 2015 Feb 2; 125(2): 501–520.

[10] Hirschfield GM, Karlsen TH, Lindor KD, Adams DH. Primary sclerosing cholangitis. Lancet 2013 Nov 9 382(9904): 1587-1599.

[11] Lalor PF, Edwards S, McNab G, Salmi M et al. Vascular Adhesion Protein-1 mediates adhesion and transmigration of lymphocytes on human hepatic endothelial cells. J Immunol. 2002 Jul 15;169(2): 983-992.

[12] Hsia LT, Ashley N, Ouaret D, Wang LM et al. Myofibroblasts are distinguished from activated skin fibroblasts by the expression of AOC3 and other associated markers. Proc Natl Acad Sci U S A. 2016 Apr 12;113(15):E2162-71.

[13] Marttila-Ichihara F, Smith DJ, Stolen C, Yegutkin GG et al. Vascular amine oxidases are needed for leukocyte extravasation into inflamed joints in vivo. Arthritis Rheum. 2006 Sep;54(9):2852-62.

[14] Biotie Therapies Corp, (2010). BTT12-CD015: A multiple ascending dose study to assess the safety and pharmacokinetics of repeated intravenous doses of BTT1023 in patients with rheumatoid arthritis - a double-blind randomized placebo-controlled sequential group trial

For peer review only

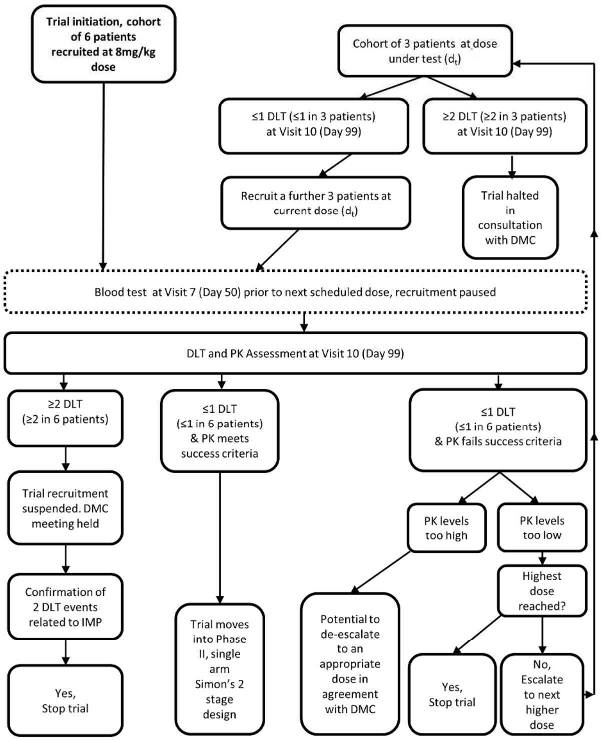

Flow diagram showing the trial decision guidelines for the run-in period of the trial design incorporating a conventional 3+3 cohort design if dose escalation is required

254x190mm (300 x 300 DPI)

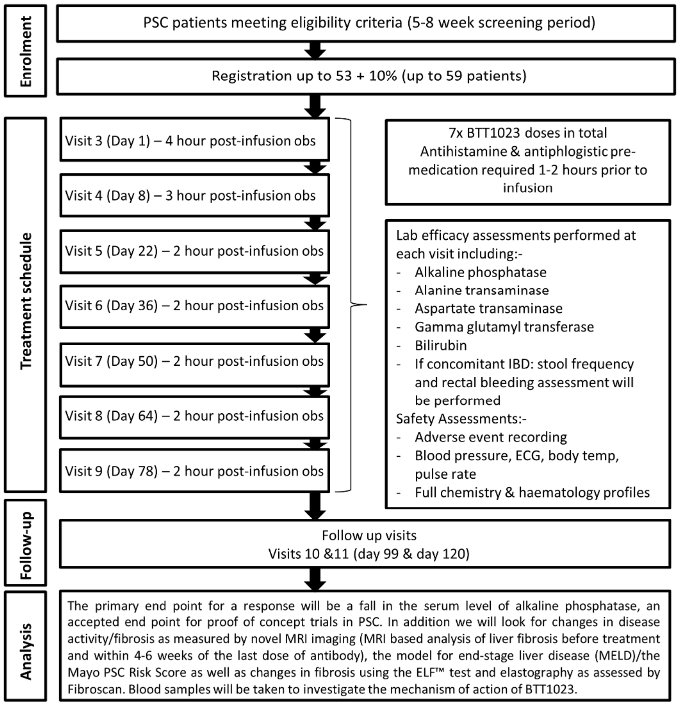

BUTEO Trial Schema

254x190mm (300 x 300 DPI)

BUTEO

SPIRIT

STANDARD PROTOCOL ITEMS: RECOMMENDATIONS FOR INTERVENTIONAL TRIALS

SPIRIT 2013 Checklist: Recommended items to address in a clinical trial protocol and related documents*

Section/item	Item No	Description	PAGE
**Administrative information**			
Title	1	Descriptive title identifying the study design, population, interventions, and, if applicable, trial acronym	2
Trial registration	2a	Trial identifier and registry name. If not yet registered, name of intended registry	4
	2b	All items from the World Health Organization Trial Registration Data Set	2,4,9-12, 18,22-25
Protocol version	3	Date and version identifier	4
Funding	4	Sources and types of financial, material, and other support	23
Roles and responsibilities	5a	Names, affiliations, and roles of protocol contributors	2,23
	5b	Name and contact information for the trial sponsor	2
	5c	Role of study sponsor and funders, if any, in study design; collection, management, analysis, and interpretation of data; writing of the report; and the decision to submit the report for publication, including whether they will have ultimate authority over any of these activities	23
	5d	Composition, roles, and responsibilities of the coordinating centre, steering committee, endpoint adjudication committee, data management team, and other individuals or groups overseeing the trial, if applicable (see Item 21a for data monitoring committee)	20
**Introduction**			
Background and rationale	6a	Description of research question and justification for undertaking the trial, including summary of relevant studies (published and unpublished) examining benefits and harms for each intervention	6-8
	6b	Explanation for choice of comparators	12
Objectives	7	Specific objectives or hypotheses	9
Trial design	8	Description of trial design including type of trial (eg, parallel group, crossover, factorial, single group), allocation ratio, and framework (eg, superiority, equivalence, noninferiority, exploratory)	10

Methods: Participants, interventions, and outcomes

			PAGE
Study setting	9	Description of study settings (eg, community clinic, academic hospital) and list of countries where data will be collected. Reference to where list of study sites can be obtained	11
Eligibility criteria	10	Inclusion and exclusion criteria for participants. If applicable, eligibility criteria for study centres and individuals who will perform the interventions (eg, surgeons, psychotherapists)	12, 25
Interventions	11a	Interventions for each group with sufficient detail to allow replication, including how and when they will be administered	10, 13
	11b	Criteria for discontinuing or modifying allocated interventions for a given trial participant (eg, drug dose change in response to harms, participant request, or improving/worsening disease)	10, 15, 18-19, 28
	11c	Strategies to improve adherence to intervention protocols, and any procedures for monitoring adherence (eg, drug tablet return, laboratory tests)	20
	11d	Relevant concomitant care and interventions that are permitted or prohibited during the trial	19
Outcomes	12	Primary, secondary, and other outcomes, including the specific measurement variable (eg, systolic blood pressure), analysis metric (eg, change from baseline, final value, time to event), method of aggregation (eg, median, proportion), and time point for each outcome. Explanation of the clinical relevance of chosen efficacy and harm outcomes is strongly recommended	15
Participant timeline	13	Time schedule of enrolment, interventions (including any run-ins and washouts), assessments, and visits for participants. A schematic diagram is highly recommended (see Figure)	10, 13, 29
Sample size	14	Estimated number of participants needed to achieve study objectives and how it was determined, including clinical and statistical assumptions supporting any sample size calculations	14, 16
Recruitment	15	Strategies for achieving adequate participant enrolment to reach target sample size	11

Methods: Assignment of interventions (for controlled trials)

Allocation:

Sequence generation	16a	Method of generating the allocation sequence (eg, computer-generated random numbers), and list of any factors for stratification. To reduce predictability of a random sequence, details of any planned restriction (eg, blocking) should be provided in a separate document that is unavailable to those who enrol participants or assign interventions	12

			PAGE
Allocation concealment mechanism	16b	Mechanism of implementing the allocation sequence (eg, central telephone; sequentially numbered, opaque, sealed envelopes), describing any steps to conceal the sequence until interventions are assigned	12
Implementation	16c	Who will generate the allocation sequence, who will enrol participants, and who will assign participants to interventions	12
Blinding (masking)	17a	Who will be blinded after assignment to interventions (eg, trial participants, care providers, outcome assessors, data analysts), and how	12
	17b	If blinded, circumstances under which unblinding is permissible, and procedure for revealing a participant's allocated intervention during the trial	12
**Methods: Data collection, management, and analysis**			
Data collection methods	18a	Plans for assessment and collection of outcome, baseline, and other trial data, including any related processes to promote data quality (eg, duplicate measurements, training of assessors) and a description of study instruments (eg, questionnaires, laboratory tests) along with their reliability and validity, if known. Reference to where data collection forms can be found, if not in the protocol	17, 20
	18b	Plans to promote participant retention and complete follow-up, including list of any outcome data to be collected for participants who discontinue or deviate from intervention protocols	16, 19
Data management	19	Plans for data entry, coding, security, and storage, including any related processes to promote data quality (eg, double data entry; range checks for data values). Reference to where details of data management procedures can be found, if not in the protocol	17
Statistical methods	20a	Statistical methods for analysing primary and secondary outcomes. Reference to where other details of the statistical analysis plan can be found, if not in the protocol	14
	20b	Methods for any additional analyses (eg, subgroup and adjusted analyses)	16
	20c	Definition of analysis population relating to protocol non-adherence (eg, as randomised analysis), and any statistical methods to handle missing data (eg, multiple imputation)	16, 19
**Methods: Monitoring**			
Data monitoring	21a	Composition of data monitoring committee (DMC); summary of its role and reporting structure; statement of whether it is independent from the sponsor and competing interests; and reference to where further details about its charter can be found, if not in the protocol. Alternatively, an explanation of why a DMC is not needed	19

			PAGE
	21b	Description of any interim analyses and stopping guidelines, including who will have access to these interim results and make the final decision to terminate the trial	16
Harms	22	Plans for collecting, assessing, reporting, and managing solicited and spontaneously reported adverse events and other unintended effects of trial interventions or trial conduct	19
Auditing	23	Frequency and procedures for auditing trial conduct, if any, and whether the process will be independent from investigators and the sponsor	20
**Ethics and dissemination**			
Research ethics approval	24	Plans for seeking research ethics committee/institutional review board (REC/IRB) approval	4,17
Protocol amendments	25	Plans for communicating important protocol modifications (eg, changes to eligibility criteria, outcomes, analyses) to relevant parties (eg, investigators, REC/IRBs, trial participants, trial registries, journals, regulators)	17
Consent or assent	26a	Who will obtain informed consent or assent from potential trial participants or authorised surrogates, and how (see Item 32)	11
	26b	Additional consent provisions for collection and use of participant data and biological specimens in ancillary studies, if applicable	11
Confidentiality	27	How personal information about potential and enrolled participants will be collected, shared, and maintained in order to protect confidentiality before, during, and after the trial	17
Declaration of interests	28	Financial and other competing interests for principal investigators for the overall trial and each study site	24
Access to data	29	Statement of who will have access to the final trial dataset, and disclosure of contractual agreements that limit such access for investigators	16
Ancillary and post-trial care	30	Provisions, if any, for ancillary and post-trial care, and for compensation to those who suffer harm from trial participation	18
Dissemination policy	31a	Plans for investigators and sponsor to communicate trial results to participants, healthcare professionals, the public, and other relevant groups (eg, via publication, reporting in results databases, or other data sharing arrangements), including any publication restrictions	4
	31b	Authorship eligibility guidelines and any intended use of professional writers	24
	31c	Plans, if any, for granting public access to the full protocol, participant-level dataset, and statistical code	24
